# Disome-seq reveals widespread ribosome collisions that promote cotranslational protein folding

**DOI:** 10.1186/s13059-020-02256-0

**Published:** 2021-01-05

**Authors:** Taolan Zhao, Yan-Ming Chen, Yu Li, Jia Wang, Siyu Chen, Ning Gao, Wenfeng Qian

**Affiliations:** 1 State Key Laboratory of Plant Genomics, Institute of Genetics and Developmental Biology, Innovation Academy for Seed Design, Chinese Academy of Sciences, Beijing, 100101 China; 2 Key Laboratory of Genetic Network Biology, Institute of Genetics and Developmental Biology, Innovation Academy for Seed Design, Chinese Academy of Sciences, Beijing, 100101 China; 3 University of Chinese Academy of Sciences, Beijing, 100049 China; 4 Peking University-Tsinghua University-National Institute of Biological Sciences Joint Graduate Program, School of Life Science, Tsinghua University, Beijing, 100084 China; 5 State Key Laboratory of Membrane Biology, Peking-Tsinghua Center for Life Sciences, School of Life Sciences, Peking University, Beijing, 100871 China

**Keywords:** Translation elongation, Disome-seq, Ribosome collision, Translational pause, Ribosome release, Ribosome-associated chaperones, Cotranslational protein folding, Protein homeostasis, Disome structure

## Abstract

**Background:**

The folding of proteins is challenging in the highly crowded and sticky environment of a cell. Regulation of translation elongation may play a crucial role in ensuring the correct folding of proteins. Much of our knowledge regarding translation elongation comes from the sequencing of mRNA fragments protected by single ribosomes by ribo-seq. However, larger protected mRNA fragments have been observed, suggesting the existence of an alternative and previously hidden layer of regulation.

**Results:**

In this study, we performed disome-seq to sequence mRNA fragments protected by two stacked ribosomes, a product of translational pauses during which the 5′-elongating ribosome collides with the 3′-paused one. We detected widespread ribosome collisions that are related to slow ribosome release when stop codons are at the A-site, slow peptide bond formation from proline, glycine, asparagine, and cysteine when they are at the P-site, and slow leaving of polylysine from the exit tunnel of ribosomes. The structure of disomes obtained by cryo-electron microscopy suggests a different conformation from the substrate of the ribosome-associated protein quality control pathway. Collisions occurred more frequently in the gap regions between α-helices, where a translational pause can prevent the folding interference from the downstream peptides. Paused or collided ribosomes are associated with specific chaperones, which can aid in the cotranslational folding of the nascent peptides.

**Conclusions:**

Therefore, cells use regulated ribosome collisions to ensure protein homeostasis.

**Supplementary Information:**

The online version contains supplementary material available at 10.1186/s13059-020-02256-0.

## Background

Translation elongation is a crucial process through which the genetic information in a transcript is sequentially decoded into a peptide chain by ribosomes. Yet, the mRNA sequence of coding regions can harbor more information than the amino-acid sequence [1]; the local rate of translation elongation is non-uniform and fine-tuned [2, 3]. Programmed variation in elongation rate may participate in the regulation of protein folding [2, 4, 5], which is challenging within the crowded and sticky cellular environments [6, 7]. A change in the rate of translation elongation can result in protein misfolding [2, 5], which further leads to developmental abnormalities, neurologic diseases, and cancers [8].

Despite the importance of translation elongation, it has been mainly studied with heterologous reporter genes [9–11]. A strong ribosomal stalling signal was often placed in these reporter genes; the 5′-elongating ribosome collides with the stalled ribosome, leading to a di-ribosome (we hereafter refer such stacked ribosomes induced by a strong ribosomal stalling signal in heterologous reporters as to di-ribosomes) or even tri-ribosome. Structure analyses indicated that di-ribosomes and tri-ribosomes were often unable to resume translation and can trigger the ribosome-associated protein quality control (RQC) pathway [9, 12–16].

The knowledge of the causes for endogenous translational pausing remains highly limited, mainly because the detection of translational pauses is technically challenging in endogenous genes. The development of ribo-seq, an approach that sequences ribosome-protected mRNA fragments at codon resolution, significantly increased our knowledge on translation elongation [17, 18]. Accumulation of ribosome footprints at a site indicates slow translation elongation (i.e., a translational pause); based on this idea, sequence determinants of translation elongation have been discovered, such as synonymous codon usage [19–21], positively charged nascent peptides [22], and mRNA secondary structures [23]. However, traditional ribo-seq misses the information of ribosome collisions [24, 25]. Instead, ribosome collisions can be studied by sequencing the mRNA fragments protected by disomes, which refer to endogenous stacked ribosomes in this study. Disomes were detectable by sucrose gradient centrifugation [26] and were observed in faulty mRNAs or 3′-untranslated regions [24]. However, the genomic landscape and the sequence determinants of endogenous ribosome collisions remain largely unknown in the coding sequences of faithfully transcribed mRNAs.

The consequence of endogenous translational pausing also remains unclear. It has been suggested that translation elongation can regulate cotranslational protein folding [2, 4, 5, 27, 28]. For example, accumulating evidence supported that the CAG expansion in Huntington’s disease led to incorrect translational pausing and thereby improper folding of the signal peptide for the subcellular localization of the Htt protein [29]. Non-optimal codons formed clusters during evolution [30, 31]; they may create slow-translation regions and participate in protein folding [32]. However, the mechanisms by which translational pauses regulate cotranslational protein folding remain understudied.

In this study, we captured the mRNA footprints protected by two stacked ribosomes in fast proliferating yeast cells. Such data provide a chance to reveal the translational dynamics that are undetectable by the traditional ribo-seq (i.e., monosome-seq). We identify the sequence features that are associated with ribosome collisions and validate some features with reporter genes. Cryo-electron microscopy (cryo-EM) analyses indicate that the majority of endogenous ribosome collisions form a different structure from the RQC-inducing di-ribosomes. With bioinformatics analyses, we show that ribosome pauses or collisions tend to take place in the gap regions between α-helices. In fact, paused or collided ribosomes are often associated with specific chaperones that can assist protein folding, as indicated by mass spectrometry analyses. As a consequence, a nascent peptide is ready to be correctly folded during translation.

## Results

### Translational pauses generate disomes from collisions of ribosomes

**Fig. 1:**
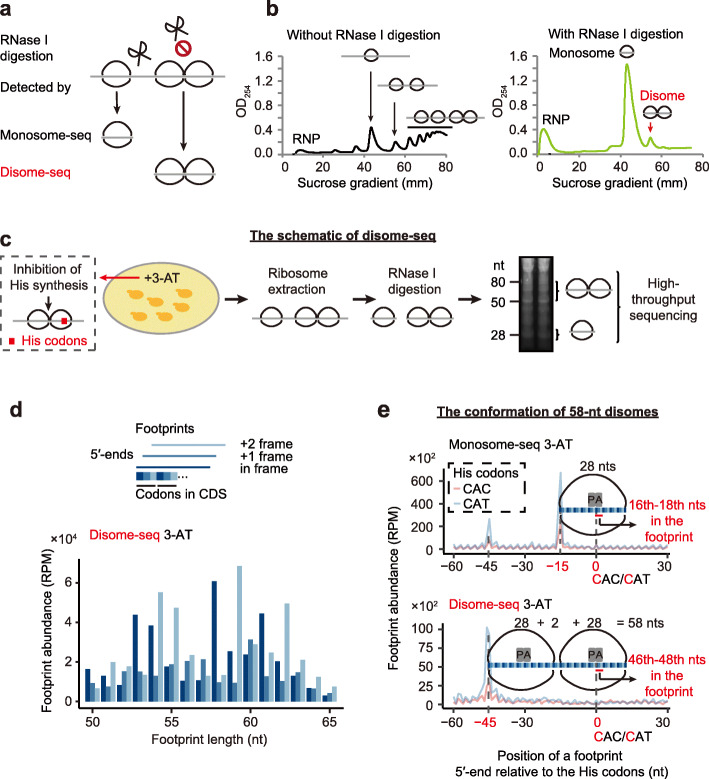
Disome-seq detects ribosome collisions. **a** A schematic explaining why traditional ribo-seq could miss the information on ribosome collisions and why the investigation of disome footprints may provide unique information on translation. **b** The disome persisted after RNase I digestion. Sucrose gradient profiles of the ribosome-mRNA complexes without (black) and with (green) RNase I digestion are shown. The *x*-axis displays the positions in the 5–50% sucrose gradient. The *y*-axis indicates the RNA abundance inferred from UV absorption (OD_254_). RNP, free ribonucleoprotein. **c** The schematic of disome-seq for 3-AT treated yeast cells. Briefly, ribosomes were extracted in a lysis buffer and digested with RNase I. Extracted RNA was separated on a polyacrylamide gel. RNA fragments with the length of approximate 20–30 nts or 50–80 nts were subjected to high-throughput sequencing. **d** The length distribution of disome footprints obtained from 3-AT treated yeast cells. Footprints are shown in different colors according to the coding frame of its 5′-end (the top panel). The average footprint abundance of two replicates is shown, in the unit of reads per million (RPM). **e** Determination of the conformation for the 58-nt disome footprints. Aggregated abundance profiles of the 5′-end of monosome (top) and disome footprints (bottom) are plotted around histidine (His) codons. Footprints were aligned by the first nucleotide of His codons (set at position 0). P and A represent the P-site and A-site of a ribosome, respectively. The 5′-end of the monosome (disome) footprints exhibited the main peak at 15-nt (45-nt) upstream of the histidine codons in the yeast genome, indicating that the A-site of the ribosome (the leading ribosome in a disome) locates at the 16th–18th (46th–48th) nts in the 28-nt monosome (58-nt disome) footprints. The average footprint abundance of two replicates is shown

Most mRNAs are associated with multiple ribosomes [33], and the speed of translational elongation varies, with some events such as tRNA depletion, known to cause ribosomes to slow down and even to pause translation [8]. We therefore hypothesized that a slowdown or pause of one leading ribosome might generate a collision between the paused ribosome and the 5′-elongating ribosome. These collisions would result in a single RNase I resistant fragment with approximately twice the footprint length of a single ribosome (Fig. 1a).

To test this idea, we extracted ribosome-bound mRNA from exponentially dividing yeast cells, digested the unprotected mRNA using RNase I, and performed sucrose gradient ultracentrifugation to separate particles of different densities. In addition to the abundant monosome particles, we observed a significant amount of particles whose density was the same as that of pre-digestion transcripts bound by two ribosomes, suggesting that the particle contains two ribosomes (Fig. 1b). These disomes persisted with increased RNase I concentration, indicating that they are not the result of incomplete digestion (Additional file 1: Fig. S1). We estimated the relative abundance of monosomes and disomes from the area ratio of the monosome and disome fractions in the profile. The ratio was approximately 16.3:1 (Fig. 1b, right panel), suggesting that the population ratio of monosomes and disomes is 32.6:1. In other words, (1/(16.3 + 1) =) 5.8% ribosomes are trapped in disomes in fast-proliferating yeast cells.

To determine if disomes were caused by paused ribosomes, we induced translational pauses at histidine codons by growing yeast in a low dose of 3-amino-1,2,4-triazole (3-AT), an inhibitor of histidine biosynthesis [34]. We performed high-throughput sequencing on monosome (monosome-seq) and disome (disome-seq) fragments (Fig. 1c, Additional file 1: Fig. S2a and Table S1–3) developed in previous studies [17, 24]. Consistent with previous observations [17], in-frame 28-nucleotide (nt) footprints were most abundant in the monosome library (Additional file 1: Fig. S3a). In contrast, the most abundant footprints in disome-seq were 58 and 59-nt when cells were treated with 3-AT, with the 58-nt footprints being in-frame (Fig. 1d). As a negative control, randomly fragmented ~ 28-nt mRNA-seq reads did not display any 3-nt periodicity (Additional file 1: Fig. S3b).

During histidine starvation, ribosomes should be paused when the histidine codon CAC or CAT is at the A-site, where decoding takes place. Consistently, the main peak in the 28-nt monosome footprints (the 5′-end) was 15-nt upstream of the histidine codons (Fig. 1e). The main peak of the 58-nt disome footprints was 45-nt upstream of the histidine codons, 30-nt upstream of the 15-nt peak from the monosome footprints (Fig. 1e). This 30-nt spacing between the two peaks perfectly fits one in-frame ribosome, suggesting that the 58-nt disome footprints were composed of two collided ribosomes of which the 3′-leading one was paused. The sharp peak in Fig. 1e also indicates that disome-seq detects ribosome collisions at codon resolution.

### Disome-seq enables detection of widespread translational pauses which cannot be identified via monosome sequencing

**Fig. 2:**
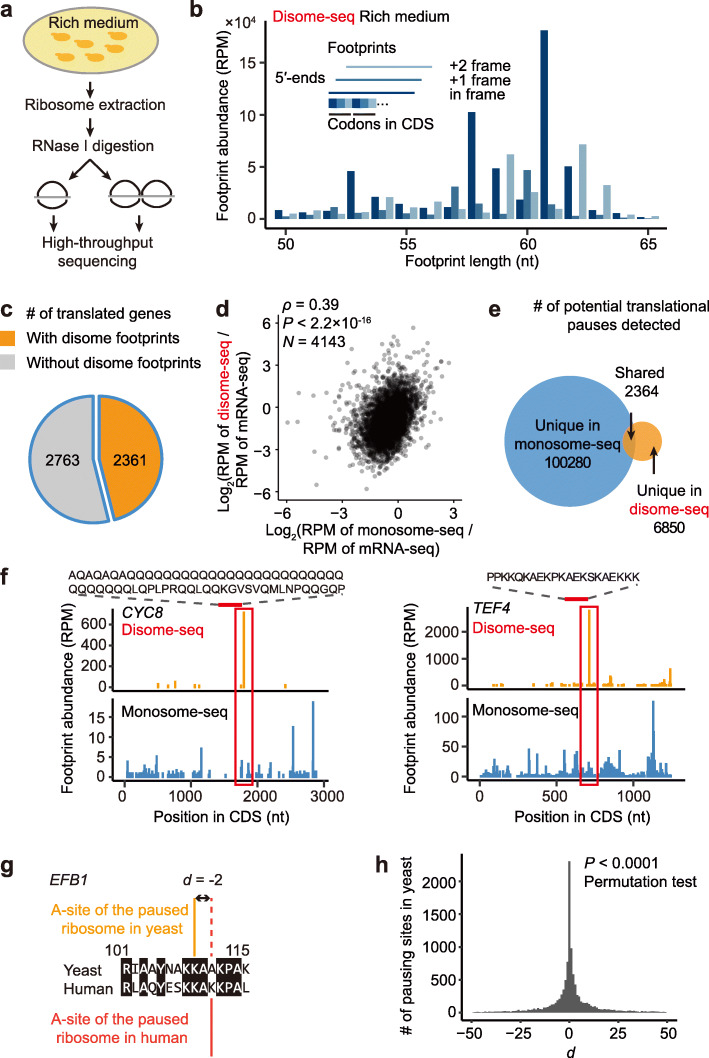
Disome-seq detects the translational pauses missed in monosome-seq. **a** The schematic of disome-seq for yeast cells cultivated in the rich medium. **b** The length distribution of disome footprints obtained from yeast cells cultivated in the rich medium. Similar to Fig. 1d. **c** Widespread ribosome collisions were detected in the yeast genome. Numbers of translated genes (genes with at least one monosome footprint) with and without disome footprints are shown in orange and gray, respectively. Footprints in two biological replicates were combined. 589,461 monosome footprints were used to define translated genes, and the disome-seq footprints were down-sampled to 18,082 footprints in order to match the population ratio between monosomes and disomes (32.6:1). **d** More frequent ribosome collision was observed in the gene with higher monosome density. **e** The translational pauses detected from monosome footprints (blue) and disome footprints (orange) rarely overlapped. Only the codon site with the footprint abundance greater than the mean of the corresponding gene was considered as a translational pause. If the A-site of a monosome footprint overlaps the A-site of the leading ribosome of a disome footprint, the translational pause is considered as “shared.” The disome-seq reads were down-sampled as in Fig. 2c. **f** Two genes exemplify the unique information of translational pauses obtained by disome-seq. The A-site of a monosome footprint (blue) or that of the leading ribosome of a disome footprint (orange) is shown along the coding sequence (CDS) of *CYC8* and *TEF4*. **g** The schematic of estimating the distance (*d*) to the closest human pause for a yeast pause. **h** The distribution of *d* between yeast and humans. The *P* value was given by the permutation test

To determine the genomic locations of ribosome collisions in fast-proliferating cells, we performed disome-seq, monosome-seq, and mRNA-seq for yeast cells growing in the mid-log phase in the rich medium (Fig. 2a, b, Additional file 1: Fig. S2b, S3c–f, and Table S1-S3). Ribosome collisions were observed in 2361 out of the 5124 translated genes (46%, Fig. 2c). This proportion remained substantial (24%, 1156/4742) when we applied a more stringent criterion—a gene with collision was called when it was supported by at least three unique molecular identifiers (UMIs, used to exclude PCR duplicates) in each biological replicate of disome-seq. In general, genes harboring more ribosomes per mRNA per unit length (i.e., ribosome density, inferred from monosome-seq) tended to exhibit higher frequency of ribosome collisions (per mRNA per unit length, *ρ* = 0.39, *P* < 2.2 × 10^−16^, *N* = 4143, Spearman’s correlation, Fig. 2d). These observations indicate widespread ribosome collisions in unstressed cells.

There are two possibilities regarding ribosome collisions inside open reading frames and the relation between translational pauses identified in monosome-seq vs. disome-seq. The first is that sites with high monosome footprint abundance identify all paused ribosomes in the cell and that the collisions identified in disome-seq are simply a subset of them. In this case, it is likely that the upstream ribosome is far 5′ of the paused ribosome, and by the time the upstream ribosome approaches, most paused ribosome has resumed elongation. In this model, all translational pauses identified by disome-seq will also be identified by monosome-seq. The second possibility is that disome-seq captures translational pauses that occur at locations not identified by monosome sequencing, possibly because the collision often occurs not long after the pause of the leading ribosome.

To differentiate these two possibilities, we measured the intersection of the translational pausing events from the two methods. Disome-seq often identified translational pauses that are undetectable in monosome-seq (Fig. 2e, f). For example, *CYC8* is a gene encoding a general transcriptional co-repressor that can fold as the prion [OCT+] [35]; a translational pause downstream of polyglutamine (polyQ) was detected by disome-seq instead of monosome-seq (Fig. 2f). Similarly, TEF4p, the γ subunit of the elongation factor eEF1B, contains a lysine-rich region that is often ubiquitinylated or succinylated [36, 37]; the downstream translational pause was uniquely detected by disome-seq (Fig. 2f). The inability of monosome-seq to fully characterize the dynamics of the ribosome along the mRNA is likely due to the omission of the disome protected mRNA fragments (Fig. 1a). Consistently, while the monosome or disome density correlated well among genes between biological replicates, the correlation between the monosome and disome densities was much weaker (Additional file 1: Fig. S2).

To determine if ribosome collisions are likely functional and thereby are conserved over evolution, we retrieved the disome-seq data generated for human cells in a recent study [38]. We projected disome footprints detected in human cells to the orthologous positions of the yeast genome based on the amino acid at the A-site of the paused ribosome. For each ribosome collision in yeast, we estimated its distance (*d*) to the closest counterpart in human cells (Fig. 2g). *d* was equal to zero in 19.4% of yeast ribosome collision sites (Fig. 2h), indicating that these yeast ribosome collisions occur at the same site in the human genome. To assess the statistical significance, we assigned ribosome collisions to random positions in human cells 10,000 times, keeping the number of ribosome collisions in each gene unchanged. The fraction of conserved ribosome collisions (*d* = 0) in every randomized sample was smaller than what we observed in the genomes (*P* < 0.0001, the permutation test, Fig. 2h), indicating that the positions of ribosome collisions are evolutionarily conserved over the 1300-million-year evolution between yeast and humans [39].

### Ribosomes tend to collide at stop codons

**Fig. 3:**
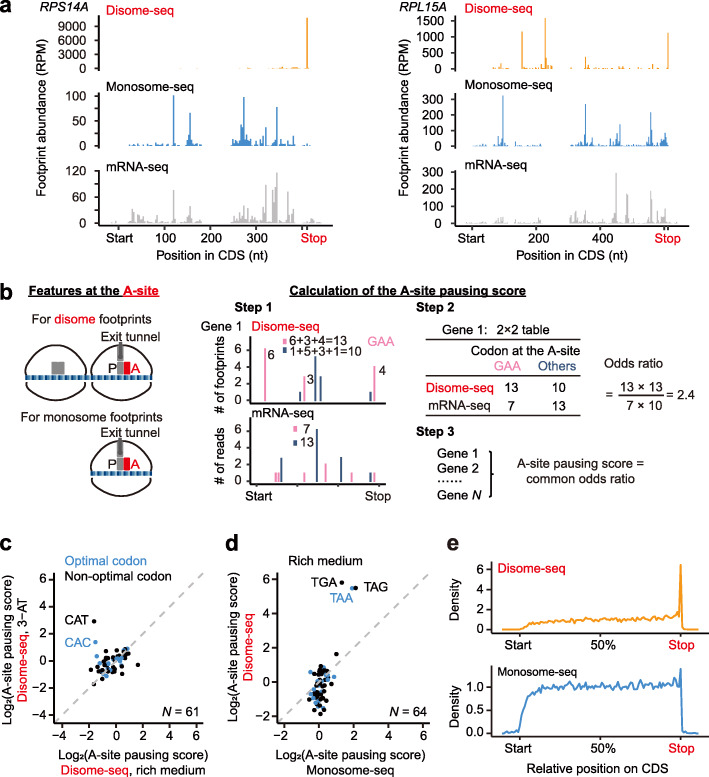
Ribosome collisions occur at stop codons. **a** Two genes (*RPS14A* and *RPL15A*) exemplify ribosome collisions at stop codons. **b** The schematic of the calculation of the A-site pausing score. We counted the numbers of disome (or monosome) footprints with the concerning codon (GAA as an example here) or the other codons (all 63 non-GAA codons) at their A-sites, respectively. The mRNA-seq reads were used to control for the codon frequency in the transcript. The A-site pausing score was defined as the common odds ratio among genes, and the *P* value was given by the Mantel-Haenszel test. **c** The scatter plot shows the A-site pausing scores estimated from cells treated with 3-AT or growing in the rich medium, for the 61 codons that encode amino acids. The dashed line shows *y* = *x*. **d** The scatter plot shows the A-site pausing scores estimated from disome-seq or monosome-seq, for cells growing in the rich medium. The dashed line shows *y* = *x*. **e** Aggregated profiles of footprint densities over 3527 (58, 59, 61, and 62-nt footprints in disome-seq, in orange) and 5230 (28 and 29-nt footprints in monosome-seq, in blue) genes, normalized against the CDS length, are shown

Visually, we noticed that many highly expressed genes exhibited large numbers of disome-seq reads at the stop codon, but also at internal codons, suggesting that ribosome collisions occur both internally and at the ends of open reading frames (Fig. 3a). To identify the causes of these collisions, we searched for the sequence features at the A-site, P-site, and exit tunnel of the 3′-paused ribosome of a disome, respectively. We defined the propensity of a codon to induce ribosome collisions at the A-site (i.e., the A-site pausing score) for each of the 64 codons as its enrichment in the disome footprints (Fig. 3b). Taking the codon GAA as an example (Fig. 3b), all disome fragments from one gene were classified into two categories based on the codon identity at the A-site, GAA or the others. The odds ratio was estimated for each gene with the corresponding codon frequency in the appendant mRNA-seq as the background; the A-site pausing score was defined as the common odds ratio across all genes calculated by Mantel-Haenszel tests (Fig. 3b). A significantly > 1 A-site pausing score implies slow decoding of the codon.

To test if the A-site pausing score defined here can effectively detect the slow decoding of the histidine codons in cells treated with 3-AT, we estimated the A-site pausing score for each codon in yeast cells treated with 3-AT or growing in the rich medium. Two histidine codons deviated from the diagonal line (Fig. 3c), indicating that the pausing score defined in this study is useful to detect translational pauses.

In comparing the disome-seq and the monosome-seq data for cells growing in the rich medium, all three stop codons exhibited extremely high A-site pausing scores in disome-seq, while their A-site pausing scores in monosome-seq were only a bit higher than the amino-acid coding codons’ (Fig. 3d). The results were consistent between two biological replicates of disome-seq (Additional file 1: Fig. S4a). To avoid artifacts generated by PCR amplification bias during the preparation of the high-throughput sequencing library, we used UMI to exclude PCR duplicates. Three stop codons still exhibited extremely high A-site pausing scores (Additional file 1: Fig. S4b). Consistently, disome reads accumulated at stop codons at the genomic scale (Fig. 3e). Collectively, these observations suggest that ribosome release is slow.

### Ribosome collisions occur preferentially at the amino acids that terminate α-helices

**Fig. 4:**
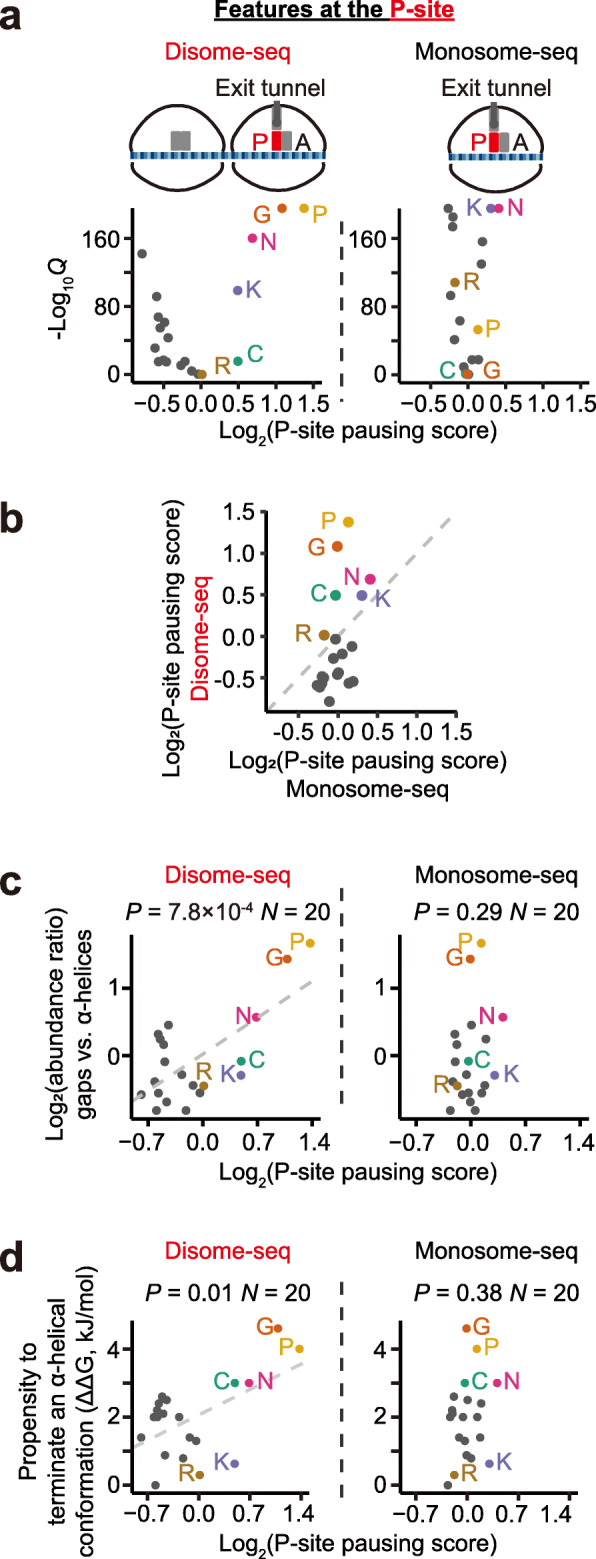
Ribosome collisions occur at the amino acids that can terminate α-helices. **a** Volcano plots show the P-site pausing score (based on disome and monosome footprints, respectively) of each amino acid and the corresponding *Q* value (false discovery rate). Six amino acids with > 1 P-site pausing scores in disome-seq are labeled. P, proline; G, glycine; N, asparagine; K, lysine; C, cysteine; R, arginine. **b** The scatter plot shows the P-site pausing scores estimated from disome-seq or monosome-seq. The dashed line shows *y* = *x*. **c** The scatter plots show the P-site pausing score of an amino acid and the ratio of the whole-genome occurrence of the amino acid in gaps vs. in α-helices. The *P* value was given by linear regression. The dashed line represents the linear regression line (if statistically significant). The residuals of both linear models followed the normal distribution (*P* = 0.63 and *P* = 0.80, the Kolmogorov-Smirnov tests). **d** The scatter plots show the P-site pausing score of an amino acid and the propensity to terminate an α-helix by the amino acid (ΔG relative to alanine, in the unit of kJ/mol). The *P* value was given by linear regression. The dashed line represents the linear regression line (if statistically significant). The residuals of both linear models followed the normal distribution (*P* = 0.25 and *P* = 0.89, the Kolmogorov-Smirnov tests)

To determine if peptide-bond formation causes ribosome collisions, we similarly calculated the P-site pausing score for each of the 20 amino acids; amino-acid identity, but not codon identity, is likely to matter at the P-site. Five amino acids (proline, glycine, asparagine, cysteine, and lysine) showed significantly > 1 P-site pausing scores in disome-seq (Fig. 4a, b). Among them, proline and glycine are known poor substrates for the formation of a peptide bond [40] and therefore slow down translation elongation and lead to ribosome collisions. It remains unclear how other amino acids induce ribosome collisions. Nevertheless, these amino acids share the same property that they weaken the stability of the α-helix [41]. Proline and glycine are conformationally either too inflexible or too flexible, respectively, to form an α-helix. The bulk and shape of asparagine and cysteine also destabilize α-helices. Positively charged residues (e.g., polylysine) likely repel each other and prevent the formation of an α-helix [41].

We estimated the number of occurrences of each amino acid in gaps or α-helices in the yeast genome; the ratio between them indicates the propensity of an amino acid to appear in the gap. We found that this ratio could be predicted from the P-site pausing score of the amino acid in disome-seq (*P* = 7.8 × 10^−4^, *N* = 20, linear regression, Fig. 4c) but not in monosome-seq (*P* = 0.29, *N* = 20, Fig. 4c). Furthermore, the propensity to terminate an α-helical conformation of an amino acid (measured by ΔΔG) [42, 43] was also predictable from the P-site pausing score in disome-seq (*P* = 0.01, *N* = 20, linear regression, Fig. 4d) but not in monosome-seq (*P* = 0.38, Fig. 4d), suggesting a sequence-mediated coupling between ribosome collisions and the intrinsically disordered region.

### Ribosome collisions tend to occur when the nascent polylysine chain is in the exit tunnel

**Fig. 5:**
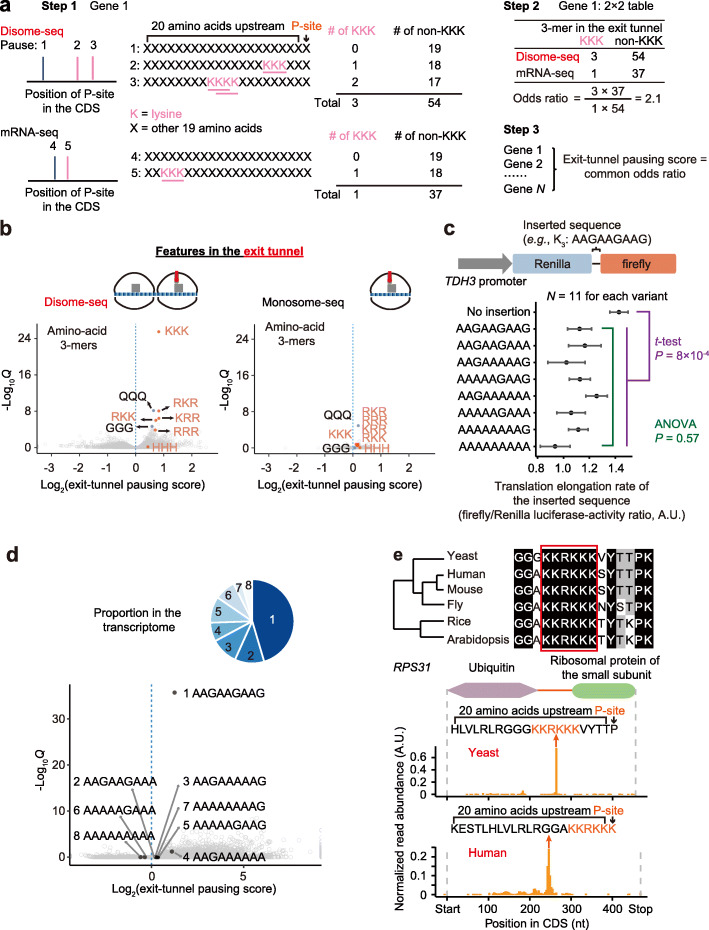
Ribosome collisions are enriched at polylysine in the exit tunnel. **a** The schematic of calculating the exit-tunnel pausing score. For each gene, we counted the numbers of the concerning amino-acid 3-mer (KKK as an example here) or the other 3-mers (non-KKK) in the 20 amino-acid region upstream of the P-site of the paused ribosome. mRNA-seq data were used as the background. The exit-tunnel pausing score was defined as the common odds ratio among genes, and the *P* value was given by the Mantel-Haenszel test. **b** The volcano plots show the exit-tunnel pausing score (based on disome or monosome footprints) of each of the 8000 amino-acid 3-mers and the corresponding *Q* value. Some 3-mers of consecutive positively charged amino acids are labeled in orange. **c** Determination of elongation rate with the dual-luciferase assay, in which the variants of different codon combinations for triple-lysine were inserted between Renilla and firefly-luciferase. Data are shown as the mean ± standard error (*N* = 11 biological replicates for each variant). ANOVA stands for the analysis of variance. **d** Similar to Fig. 5b, the volcano plot shows the exit-tunnel pausing score for disome footprints of each of the 61^3^ codon 3-mers and the corresponding *Q* value. The eight codon combinations for triple-lysine are highlighted. The pie chart shows the relative abundance of the eight codon combinations for triple-lysine in the yeast transcriptome. **e** A putative pausing signal inducing ribosome collisions (KKRKKK) was conserved in the linker region between two domains of Rps31p, across the eukaryotic kingdom (top). The ribosome collision with the putative pausing signal located within the exit tunnel was also conserved between yeast and human (bottom). The footprint abundance at a site was normalized by the total footprints of *RPS31* in the corresponding species

Positively charged nascent peptides can hinder translation elongation by interacting with the negatively charged exit tunnel [22]. To test this hypothesis with disome-seq data, we calculated the exit-tunnel pausing score from the enrichment of each of the (20^3^ =) 8000 amino-acid 3-mers in the 20-amino-acid region upstream of the P-site of the leading ribosome. Taking triple-lysine as an example (Fig. 5a), a window of three amino acids slid one amino acid per step in this 21-amino-acid region, counting the number of triple-lysine or the total number of all other 3-mers for each pausing site; these numbers were added together for all pausing sites of a gene. The odds ratio was estimated for each gene with the corresponding numbers in the appendant mRNA-seq data as the background. The exit-tunnel pausing score was defined as the common odds ratio across the genome aggregated by the Mantel-Haenszel test.

Ribosome collisions were strongly associated with positively charged 3-mers (e.g., RKR) when in exit tunnels; by contrast, such pausing signals were obscured in monosome-seq (Fig. 5b). Triple-lysine had the strongest collision signal among all the 3-mers (Fig. 5b), much stronger than other positively charged 3-mers (e.g., RKR, RKK, KRR, and RRR). It has been suggested that the reading-through of stop codons could result in the translation of poly(A) tails into polylysine, which activates the RQC pathway [44]. As a result, A-tracts are often used to induce RQC in heterologous reporter systems [10]. Since that AAA is the non-optimal codon of lysine [45], it is unclear if its poor codon optimality also participates in promoting translational pausing in these experiments.

To experimentally determine if combinations of synonymous codons for triple-lysine have different efficacies to induce translational pauses, we constructed a dual-luciferase reporter system by inserting individual synonymous variants of triple-lysine between the Renilla and firefly-luciferases (Fig. 5c). A reduction in the firefly/Renilla luciferase-activity ratio can report translational pausing induced by the inserted sequence—translation elongation replaces initiation as the rate-limiting step in protein synthesis of the downstream luciferase. We did not detect significant differences in the firefly/Renilla ratio among the eight synonymous codon combinations of triple-lysine (*P* = 0.57, analysis of variance, Fig. 5c). In contrast, the firefly/Renilla ratio was significantly reduced when triple-lysine was inserted (*P* = 8 × 10^−4^, the two-tailed *t* test, Fig. 5c), suggesting that lysine residues, instead of the non-optimal AAA codons, are accountable for the ribosome collisions in A-tracts.

This observation is not unexpected because it is the identity of amino acids rather than the identity of synonymous codons that should matter once the nascent peptide is in the exit tunnel. Consistently, when we separately estimated the pausing scores for the eight synonymous codon combinations encoding triple-lysine (Fig. 5d), endogenous ribosome collisions induced by triple-lysine were mainly contributed by triple-AAG (Fig. 5d), the most abundant codon combination for triple-lysine in the yeast transcriptome (Fig. 5d).

Polylysines often appear in the gap regions because the repellence among the positively charged residues prevents the formation of an α-helix [41]. For example, polylysine-induced ribosome collision occurs in the linker between the two domains of RPS31p, the fusion protein of ubiquitin and ribosomal protein (Fig. 5e), raising the possibility that a translational pause benefits the cell by providing sufficient time for the cotranslational folding of the upstream domain. Consistently, despite being in an intrinsically disordered region, these positively charged amino acids (KKRKKK) are conserved across eukaryotes (Fig. 5e). To test if the ribosome collision is also conserved, we retrieved the disome-seq data for human cells reported in a recent study [38]. The ribosome collision, with these positively charged nascent peptides in the exit tunnel, was detected in both human and yeast cells (Fig. 5e), albeit these two species have diverged since 1300 million years ago [39], suggesting a function of this inter-domain ribosome collision.

### Disomes exhibit a different structure from the RQC-inducing di-ribosomes

The pervasiveness of ribosome collisions detected in fast-proliferating cells (Fig. 2) and the ubiquitous sequence signals of disomes (e.g., proline and polylysine, Figs. 4 and 5) in endogenous genes are astonishing since the stacked ribosomes observed previously in reporter genes (di-ribosomes) could lead to the decay of both mRNA and nascent peptides through the no-go decay and the RQC pathways [46]. Alternatively, some disome footprints detected in fast proliferating cells may simply reflect the inevitable nature of stochastic and temporary ribosome collisions since an mRNA is often translated simultaneously by multiple ribosomes. For example, in the top 2% of highly translated mRNAs, on average ~ 3 ribosomes were located in a 100-nt region (Additional file 1: Fig. S5), a highly crowded situation for ribosomes.

Consistent with this idea, collision propensity of a transcript was positively correlated with ribosome density (Fig. 2d)—the 3′-paused ribosomes are easier to be caught up by a 5′-elongating ribosome on mRNAs with high ribosomal flux. We speculate that these ribosome collisions were neither induced by faulty mRNA nor by aberrant nascent peptides, and it would be very costly for cells to activate the no-go decay or the RQC pathway for these ribosome collisions. Their translation is likely transiently interrupted and to be resumed. If true, we predict that these disomes should be structurally different from the RQC-inducing di-ribosomes reported in previous studies.

**Fig. 6:**
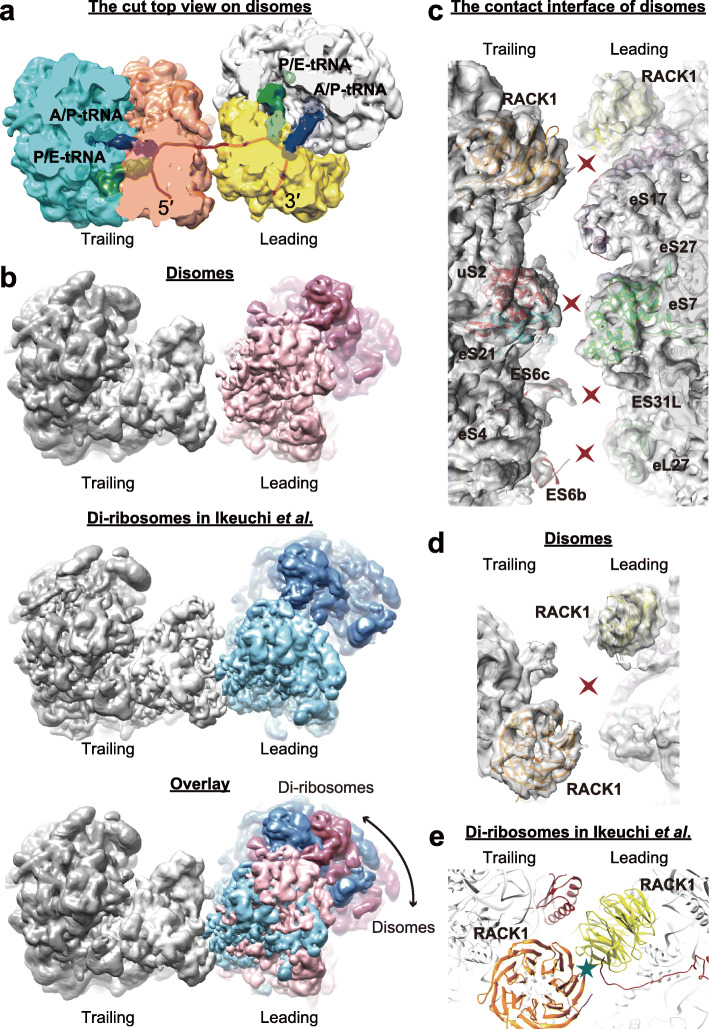
The cryo-EM structure of disomes. **a** A composite map of the cryo-EM structure of disomes. The disomes were collected without cycloheximide in the lysis buffer. Both the leading and the trailing ribosomes are in the rotated state harboring hybrid A/P (blue) and P/E tRNAs (green). Small subunits are labeled by orange and yellow, and large subunits are labeled by cyan and gray. A model of mRNA is highlighted in red. **b** The top views on disomes (top) and di-ribosomes (middle). The structure of di-ribosomes was from Ikeuchi et al. [9]. An overlay is shown (bottom) by docking di-ribosomes to disomes, aligned according to the trailing ribosomes; the conformational difference in the leading ribosomes is highlighted by the arrow. **c**–**e** The zoomed-in detail of the contact interface between the 40S subunits of the leading and the trailing ribosomes, in particular, the 40S head-to-head contact site of disomes (**d**) and that of di-ribosomes (**e**). The blue star indicates a strong interaction between the two ribosomes in di-ribosomes, and the red crosses indicate the missing interactions in disomes

To test if the disomes collected from endogenous genes in fast-proliferating cells are structurally identical to the RQC-inducing di-ribosomes, we performed cryo-EM analyses of disomes collected after RNase I digestion. Following the previous protocols for the structural determination of di-ribosomes or tri-ribosomes [9, 15, 16], we omitted the elongation inhibitor cycloheximide in the lysis buffer. Disomes showed a structure of two ribosomes linked by a bent mRNA, with two 40S subunits facing toward each other (Fig. 6a). Furthermore, the 5′-trailing ribosomes were detected in the rotated state that harbors hybrid A/P and P/E tRNAs (Fig. 6a), indicating that they cannot further translocate, likely due to the road-blocking 3′-leading ribosomes. These observations are in general consistent with the structure of di-ribosomes induced by hard stalls in cell-free systems [9, 15, 16].

However, two distinct features indicate the essential difference between disomes and di-ribosomes. First, the relative orientation between the two ribosomes in the disome was different from that in the RQC-triggering di-ribosome (Fig. 6b). It was reported that the two ribosomes in the di-ribosome form a large interface with highly specific contacts between two 40S subunits (Additional file 1: Fig. S6a), which was considered to be a feature recognizable by the RQC initiation factors such as Hel2p [9, 11, 16, 47]. However, in the disome, the interface between the two ribosomes was relatively flexible; the contacts between the two ribosomes were much weaker or nearly absent in our structure of the disome (Fig. 6c, Additional file 1: Fig. S6b-h). For instance, a tight interaction was reported between the two RACK1 (Asc1p)—the protein involved in the initiation of RQC—from the two ribosomes in the di-ribosome [9]. However, the interaction was completely lost in the disome (Fig. 6d, e), due to the different orientation of the two 40S subunits (Fig. 6b). These observations suggest that the disome may lack specific structural features required for recruiting RQC factors, such as Hel2p.

Second, the leading ribosome of the majority (31,010/35,918 = 86.3%) of disomes was detected in the rotated state (Fig. 6a), in sharp contrast to the non-rotated state (with P/P and E/E tRNAs) reported for the leading ribosome in di-ribosomes. The non-rotated state has been previously reported as a feature of stalled 80S ribosomes before collisions [9], and therefore, the rotated state of the leading ribosome indicates that these disomes are likely induced by transient pauses. Collectively, the results of cryo-EM analyses indicate that the widespread disomes observed in vivo (Fig. 2c) are structurally different from di-ribosomes that are recognized and resolved by the RQC pathway.

### Ribosomes are associated with specific chaperones

**Fig. 7:**
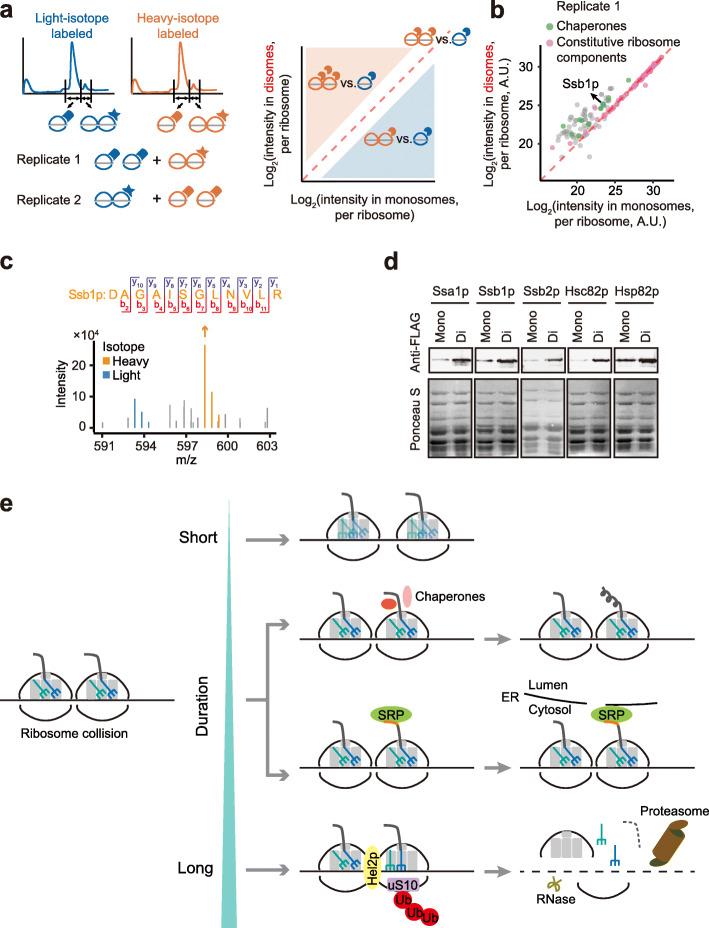
Collided ribosomes are associated with specific chaperones. **a** The schematic of detecting the disome-enriched proteins through SILAC. Two biological replicates were performed with reciprocal labeling (the left panel). Because equal amount of total protein extracted from the monosome fraction and disome fraction were mixed, proteins having the same abundance per ribosome between monosomes and disomes should be located on the diagonal (the right panel). Proteins locating above or below the diagonal should be those enriched in the disomes or the monosomes, respectively. **b** The protein intensities per ribosome in disomes were plotted against those in monosomes. Chaperones (green) are enriched in the disome, whereas most constitutive ribosomal proteins (pink) show the same abundance per ribosome between monosomes and disomes. **c** The m/z (mass divided by charge number) spectrum of Ssb1p and its fragments detected in MS/MS. **d** The enrichment of chaperones in disomes. Immunoblotting was performed using anti-FLAG antibody, with Ponceau S stained total protein as the loading control. Mono and Di represent proteins extracted from the monosome and disome fractions, respectively. **e** A schematic of the fates of ribosome collisions with various durations. The double-rotated disome conformation is used to show the initial status for ribosome collisions (left). The signal peptide is highlighted in orange. SRP, signal recognition particle; ER, endoplasmic reticulum; Ub, ubiquitin

To understand the function of endogenous ribosome collisions, we attempted to identify disome-specific ribosome components. We labeled ribosome proteins with stable isotopes, digested the mRNA with RNase I, and separated disomes from monosomes with sucrose density gradient centrifugation. We mixed an equal amount of protein extracted from the heavy-labeled disome fraction with light-labeled monosome fraction (or light-labeled disome fraction with heavy-labeled monosome fraction in a label-swap replicate) and performed tandem mass spectrometry (MS/MS, Fig. 7a).

In addition to ribosomal proteins, some highly expressed metabolic enzymes were also identified (Additional file 1: Fig. S7, and Table S4, S5); for two reasons, we speculate that they are nascent peptides of incomplete protein translation attached to the ribosomes. First, the peptides of these enzymes captured by MS/MS tended to be in the first half of the coding sequences (the common odds ratio = 1.9, *P* = 0.001, the Mantel-Haenszel test). For example, all detected peptides of FAS2p, a fatty acid synthetase, were in the first half (Additional file 1: Fig. S7b). By contrast, the peptides of ribosomal proteins captured by MS/MS were evenly distributed in the first and second half of the coding sequences (the common odds ratio = 1.0, *P* = 0.7). Second, among the 26 metabolic enzymes, the protein abundance of a metabolic enzyme in the disome fraction was positively correlated with the abundance of disome footprints on its encoding gene (*ρ* = 0.57, *P* = 2.5 × 10^−3^, *N* = 26, Spearman’s correlation, Additional file 1: Fig. S7c). By contrast, we did not detect such correlation for ribosomal proteins (*ρ* = 0.16, *P* = 0.08, *N* = 129, Spearman’s correlation, Additional file 1: Fig. S7c); this is not unexpected since most ribosomal proteins were likely captured by MS/MS as ribosome components instead of as nascent peptides.

Most proteins have similar abundance per ribosome in disomes and in monosomes (e.g., two copies in a disome and one copy in a monosome) and therefore were on the diagonal in the scatter plot showing protein intensities in disomes vs. in monosomes; many were constitutive ribosome components (Fig. 7b and Additional file 1: Fig. S7a). Proteins above the diagonal line were the disome-enriched proteins (Fig. 7b, Additional file 1: Fig. S7a, and Table S4, S5). Proteins involved in the RQC pathway such as Hel2p, Slh1p, Cue3p, and Rqt4p [9, 16, 48] were not observed significantly enriched in disomes (Additional file 1: Table S4, S5).

In contrast, all 11 chaperones identified by our MS/MS analysis had > 1.5-fold per-ribosome abundance in disomes than in monosomes (*P* = 1 × 10^−6^, Fisher’s exact test, Fig. 7b; *P* = 1 × 10^−4^, Additional file 1: Fig. S7a). For example, although SSB1p was reported as a ribosome-associated chaperone [49], how SSB1p is distributed among ribosomes remained unclear. The MS/MS analyses indicate that SSB1p is associated primarily with disomes (Fig. 7b, c). To validate this result, we randomly chose five out of these 11 chaperones and fused 4×FLAG to the C-terminus for each of them. The enrichment in disomes was confirmed with the immunoblotting assay for all five chaperones (Fig. 7d). These observations suggest that some chaperones are associated with ribosome collisions, capable of participating in the folding of nascent peptides.

## Discussion

### Caveats

There are at least four caveats in this study since we assumed that the abundance of disome footprints faithfully reflects the intensity of ribosome collisions at a genomic location. First, if there is any relation between disome footprints and the transcripts binding to two ribosomes due to incomplete RNase I digestion, disome footprints may not always indicate ribosome collisions. To test this possibility experimentally, we collected the transcripts binding to two ribosomes, performed RNase I digestion, and sequenced ribosome-protected mRNA fragments (Additional file 1: Fig. S8a). The footprints repeatedly observed in disome-seq did not show up in such experiments (Additional file 1: Fig. S8b as an example), indicating that disome footprints are fundamentally different from the transcripts binding to two ribosomes, in which the two ribosomes are likely sparsely distributed, and the mRNA fragment between them is sensitive to RNase I.

The second confounding factor is ligation bias. RNA fragments with various 5′-nucleotide compositions may have variable ligation efficiency with a fixed 5′-adaptor sequence during the preparation of the high-throughput sequencing libraries [50, 51]. To reduce such ligation bias, we had added three random nucleotides to the 3′-end of the 5′-adaptor during library preparation. To assess how much ligation bias is remained, we calculated the frequency for each of the sixty-four 3-mers in the −15th to −13th nt region upstream of the A-site of monosome footprints, as well as in the −45th to −43th nt region upstream of the A-sites of the leading ribosomes of disome footprints. Both were positively correlated with the genome-wide tri-nucleotide composition in the coding sequences (Additional file 1: Fig. S9a-c), indicating that ligation bias has been largely eliminated from our monosome-seq and disome-seq libraries.

Nevertheless, we realized that any ligation bias not entirely eliminated could have led to discrepancies in the translational pauses detected by disome-seq and monosome-seq. It is because a translational pause generates different 5′-end for monosome and disome footprints (Additional file 1: Fig. S9a). To assess such possibility, we estimated the 3-mer frequencies in the −15th to −13th nt region upstream of the A-sites (for disomes, the A-site of the leading ribosome) for translational pauses detected in disome-seq or monosome-seq, respectively. Such 3-mers are the 5′-end of the 28-nt monosome footprints but are the internal sequences of disome footprints. Although monosome-seq and disome-seq identify largely non-overlapping pausing sites (Fig. 2e), the 3-mer frequency in the −15th to −13th nt region upstream of the A-sites were positively correlated between the two experiments (*ρ* = 0.95, *P* < 2.2 × 10^−16^, *N* = 64, Spearman’s correlation, Additional file 1: Fig. S9d). This observation indicates that ligation bias cannot be a major explanation for the discrepancy in the locations of translational pauses identified by disome-seq or monosome-seq.

A third caveat is related to the background used in the Mantel-Haenszel test for calculating the pausing scores. We performed appendant mRNA-seq experiments that mRNA was sheared into < 50-nt fragments using a high-salt buffer, instead of the RNase I digestion as in the monosome-seq and disome-seq experiments. The purpose of using such mRNA-seq data as the background in our analyses (Fig. 3, 4, 5) was to control for any potential ligation bias, amplification bias, or sequencing bias in the high-throughput sequencing. Nevertheless, we realized that another approach was to use codon or amino-acid frequencies in each gene as the background (Additional file 1: Fig. S10a). We recalculated the A-site and P-site pausing scores using this approach and found them virtually unchanged (Additional file 1: Fig. S10b, c), indicating that the reported pausing scores in this study are computationally robust.

Last, it is worth noting that the ribo-seq protocol has been “evolving” since its debut in 2009 [17]. In the initial protocol, yeast cells were co-cultured with cycloheximide, a step that was later shown distorting the location of ribosome footprints within genes [19, 20, 52]. Furthermore, the addition of cycloheximide, as well as other antibiotics, in the lysis buffer will stabilize ribosomes at various conformations that protect mRNA fragments of different sizes [51, 53]. The size selection for ribosome footprints has also been shown to have unexpected effects on the identification of translational pausing sites [54]. We avoided co-culturing yeast cells with cycloheximide but added cycloheximide in the lysis buffer to stabilize ribosomes, following recently updated ribo-seq protocols [19, 20, 53, 55]. Nevertheless, it is reasonable to assume that although disome-seq represents some advance in understanding ribosome collisions, it inevitably reflects only part of the whole story, due to technical limitations of the experimental procedures.

Similarly, it remains unclear how the chemicals in the lysis buffer could have affected the structural determination of disomes by cryo-EM. In addition to the disomes collected with cycloheximide omitted from the lysate (Fig. 6, Additional file 1: Fig. S6), we also determined the structure of the disomes extracted with cycloheximide added to the lysis buffer (as in the disome-seq experiment). These disomes held the two structural features we reported in Fig. 6: a markedly different interface from the di-ribosome and a rotated state for the leading ribosome (Additional file 1: Fig. S11a-c). Nevertheless, the structural determinations of disomes under various experimental conditions are recommended in the future for a more comprehensive understanding of ribosome collisions.

### The 61-nt disome footprints

Although not highlighted in our results, note that we did conduct additional analyses to examine the length distribution of disome footprints. In addition to the 58-nt disome footprints whose conformation was determined in Fig. 1e as two 28-nt monosome footprints spaced by two nts, the 5′-end of the 61-nt fragments also exhibited an apparent 3-nt periodicity (Fig. 1d, 2b), suggesting the presence of a second disome conformation. The disomes accumulated at the stop codons (Additional file 1: Fig. S12a) indicate that the 61-nt disome footprints were composed of two 28-nt monosome footprints spaced by five nts. The fate of such 61-nt disomes is likely determined by which of the two events occurs first. If the leading ribosome resumes translation first, the ribosome collision is resolved; alternatively, if the trailing ribosome moves first, probably because the leading ribosome has paused for a long time, a 58-nt disome is formed.

The 61-nt disome footprints were more abundant than the 58-nt disome footprints in cells growing in the rich medium (Fig. 2b, Additional file 1: Fig. S13a). The abundance of the 61-nt disome footprints was reduced when cells were treated with 3-AT (Fig. 1d, Additional file 1: Fig. S13a), a phenomenon that was also observed in a recent study [56]. We speculate that the translational pauses at the histidine codons induced by the 3-AT treatment last longer than the time frame of an average translational pause for yeast cells growing in the rich medium, thereby providing additional time for one more move of the trailing ribosome toward the leading ribosome.

To determine if the distinction between the 58-nt and the 61-nt disome footprints can provide additional information for the strength of a ribosome collision, we separately estimated the pausing scores from the 58-nt or the 61-nt disome footprints. They were highly correlated (for the A-site, *ρ* = 0.92, *P* < 2.2 × 10^−16^, *N* = 64; for the P-site, *ρ* = 0.88, *P* = 3.2 × 10^−7^, *N* = 20, Spearman’s correlations, Additional file 1: Fig. S12b), indicating the absence of length-specific collision signals between the 58-nt and the 61-nt disome footprints. To improve the statistical power, we combined the 58 and 61-nt footprints for the analyses in Fig. 3, 4, 5. A recent investigation on mouse liver also reported two lengths of disome footprints, 59–60 nts and 62–63 nts; similar to our observations, nearly identical pausing sequences were reported for the disome footprints of these two lengths [57].

### The 53-nt disome footprints

In addition to the 58-nt and the 61-nt disome footprints, we observed the 53-nt disome footprints, which showed an apparent 3-nt periodicity (Fig. 1d, 2b) and accounted for 10.9% of disome footprints for yeast cells growing in the rich medium (Additional file 1: Fig. S13a). This indicates the existence of a third disome conformation. When treated with 3-AT, the 5′-end of most 53-nt disome footprints was 45-nt upstream of the histidine codons (Additional file 1: Fig. S13b), the same position as in the 58-nt disome footprints (Fig. 1e). It indicates that the positions of the A-site of the leading ribosome relative to the 5′-end are identical between the 53-nt and the 58-nt footprints. This observation echoes the two lengths of monosome footprints reported in previous studies, ~ 21 nts and ~ 28 nts [51, 53]; these two footprints share the same 5′-end but show different 3′-end. The 28-nt footprints represent the ribosomes whose A-sites are occupied (in the rotated state with hybrid A/P and P/E tRNAs or the non-rotated state with A/A and P/P tRNAs); the length of footprints reduces to ~ 21 nts when the A-site of a ribosome is open (in the non-rotated state with P/P and E/E tRNAs) [53].

In alignment with the two lengths of monosome footprints, we speculate that the (28 + 2 + 23 =) 53-nt disome footprints represent a disome conformation that the A-site of the leading ribosome is open, whereas the (28 + 2 + 28 =) 58-nt disome footprints represent a conformation that the A-site of the leading ribosome is occupied. If true, we predict that the abundance of the 53-nt disome footprints will increase when cells are treated with 3-AT; it is because 3-AT induces ribosome collisions mainly through a prolonged time frame for decoding histidine codons, the very state that the A-site of the leading ribosome is open. The proportions of the 53-nt footprints indeed increased from 10.9% for cells growing in the rich medium to 17.9% for cells treated with 3-AT (Fig. 1d, 2b, Additional file 1: Fig. S13a), in support of our interpretation of the 53-nt disome footprints.

If the 53-nt disome footprints represent a disome conformation that the A-site of the leading ribosome is open, we further predict the existence of disome structure that the leading ribosome is in the non-rotated state with P/P and E/E tRNAs (presumably protecting 23-nt mRNA fragment) and the trailing ribosome is in the rotated state with hybrid A/P and P/E tRNAs (protecting 28-nt mRNA fragment). Indeed, we observed that such conformation in (4908/35,918 =) 13.7% of the disomes collected from yeast cells growing in the rich medium (Additional file 1: Fig. S11d). Nevertheless, the specific contacts between the two 40S subunits observed in di-ribosomes [9, 16] remained absent in these disomes (Additional file 1: Fig. S11e).

For cells growing in the rich medium, the stop codons were less representative in the A-site of leading ribosomes in the 53-nt disome footprints (Additional file 1: Fig. S13c, d), echoing the 52-nt and 54-nt disome footprints recently reported in human cells [38]. In the study, the 52-nt and 54-nt footprints were protected by the disomes that the A-site of the leading ribosome was open and tended to locate in coding sequences compared to the stop codon. On the other hand, the 58 and 61-nt disome footprints detected in our study are similar to the ~ 61-nt footprints reported in human cells; they showed a stronger tendency to locate at stop codons (Fig. 3e, Additional file 1: Fig. S13c) [38]. The reduced enrichment of the 53-nt disome footprints at stop codons (Fig. 3e, Additional file 1: Fig. S13c, d) suggests that stop codons are rapidly occupied by release factors in the A-site of ribosomes; the translation is slow at stop codons probably because of the prolonged subsequent steps disassembling the post-termination ribosomal complexes [58].

If the 53-nt and the 58-nt/61-nt footprints are protected by disomes that the A-site of the leading ribosome is open and occupied, respectively, we reasoned that the 58-nt/61-nt disome footprints should be more informative than the 53-nt ones in detecting the translational pauses associated with peptide bond formation and translocation. It is because these processes slow down translation elongation only if the A-site of a ribosome is occupied. Indeed, when calculated from the 53-nt disome footprints, the P-site pausing score of proline, the amino acid that is well known as a poor acceptor for peptide bond formation [40, 46], was no longer significantly > 1; such pausing signal was observed for the 58-nt disome footprints (Fig. 4a, Additional file 1: Fig. S13e).

We further reasoned that the 53-nt disome footprints should be more informative than the 58-nt ones in detecting the effect of codon optimality, because codon optimality causes translational pauses only if the A-site of a ribosome is open [53]. However, we did not observe more ribosome collisions associated with non-optimal codons than optimal codons using either the 53-nt disome footprints (*y*-axis in Additional file 1: Fig. S13c, *P* = 0.47, *N* = 61, Mann-Whitney *U* test) or the 58-nt/61-nt disome footprints (*x*-axis in Additional file 1: Fig. S13c, *P* = 0.85, *N* = 61, Mann-Whitney *U* test). In fact, the A-site pausing score for the 61 amino-acid codons was highly correlated between the 53-nt and the 58-nt/61-nt disome footprints (Additional file 1: Fig. S13c, *ρ* = 0.56, *P* = 3.5 × 10^−6^, *N* = 61, Spearman’s correlation). Our observation is consistent with Han et al., who reported in human cells that synonymous codon usage/tRNA supply was poorly associated with the propensity for ribosome collisions [38].

Synonymous codons are known to be recognized at variable rates [19, 20, 53], which further regulate mRNA stability [31, 59]; however, the impact of codon optimality on ribosome collisions was not detected in disome-seq. One explanation is that decoding is a relatively rapid step in the work cycle of translation elongation [60]. Therefore, its potential in inducing variable ribosome collisions is masked by variation in the duration of other steps such as peptide bond formation. Consistently, when the decoding time became a dominant factor in the work cycle of translation elongation as the yeast cells were treated with 3-AT, the non-optimal codon of histidine, CAT, did induce more ribosome collisions than the optimal codon, CAC (Fig. 1e, Additional file 1: Fig. S13b, f).

### The evidence for the existence of trisomes

In addition to disomes, translational pauses may further lead to longer queues of collided ribosomes, for example the collision of three ribosomes—trisomes [15, 56, 61]. Indeed, after RNase I digestion, a small number of particles were observed at the ~ 62 mm position after sucrose gradient centrifugation in Fig. 1b; the density of these particles was higher than disomes and was similar to transcripts binding to the three ribosomes, suggesting that they were trisomes. Although the abundance of these trisomes was insufficient for a trisome-seq experiment, the presence of trisomes upstream of stop codons was evident by the disome footprints with the 5′-end ~ 75-nt upstream of the stop codon (Additional file 1: Fig. S12c).

### The absence of translational “ramp”

Yeast genes often use more non-optimal codons in the 5′ region of the coding sequences, which has been speculated to serve as a translational “ramp” to reduce downstream ribosome collisions [62]. However, we did not find the signals that non-optimal codons induced more ribosome collision (Fig. 3d, Additional file 1: Fig. S13c) nor did we detect any accumulation of ribosomes downstream of the start codon in either monosome-seq or disome-seq (Fig. 3e). It is likely that the previously observed accumulation of ribosomes around the start codon [17] is a byproduct of the co-culturing with cycloheximide [19, 20, 61] that partially inhibits translation elongation but does not block initiation [63].

Instead, we observed more ribosome collisions downstream of a transcript (Fig. 3e), an “inverse ramp” that in our view can be partly explained by two mutually non-exclusive mechanisms. First, ribosomes tend not to collide in the upstream region of an mRNA due to a steric effect. The small subunit occupies ~ 37 nts of mRNA during subunit joining, with 22 nts downstream of the start codon [64]. This conformation requires the 5′-end of the leading ribosome at least 24-nt downstream of the start codon; when translation elongation starts, the start codon is at the P-site of the ribosome, indicating that such ribosomes occupy 13 nts downstream of start codons (Additional file 1: Fig. S14a). Therefore, such a steric effect sets a minimum 11-nt distance between two ribosomes at translation initiation, reducing the possibility of ribosome collisions in the 5′-region of coding sequences. For example, even if the leading ribosome stays still, it requires three moves of the trailing ribosome to collide (Additional file 1: Fig. S14a). Consistent with this model, disomes were missing entirely in the first nine nts of the open reading frame of any gene (Additional file 1: Fig. S14b). The collisions propensity increases gradually along the coding sequence, reaching a plateau ~ 50 nts after translation starts, as indicated by the meta-gene analysis of the disome-seq data (Additional file 1: Fig. S14b).

A second explanation is that translation termination is relatively slow compared to elongation, resulting in ribosomal “traffic jam” upstream of stop codons; abundant disomes (Fig. 3e) and even some trisomes were formed close to stop codons (Additional file 1: Fig. S12c). It can be reasonably generalized that the elevated ribosome density due to slow termination can result in more frequent ribosome collisions in a region longer than the ~ 90 nts that are occupied by trisomes.

Note that both steric effects aforementioned, ribosomes lining up equidistantly downstream of start codons and queueing upstream of stop codons for translation termination and recycling, should have greater impacts on shorter genes, for two reasons. First, ribosomal flux is generally higher in shorter genes [33], amplifying both steric effects. Second, the same affected genomic region (e.g., a 90-nt region occupied by trisomes) will take up a larger fraction of the coding sequence in shorter genes. Consistently, the inverse ramp appears to be steeper among genes with shorter coding sequences (Additional file 1: Fig. S14c).

### Additional causes of ribosome collisions

In addition to the sequence features identified at the A-site, the P-site, and the exit tunnel (Fig. 3, 4, 5), sequences outside of the ribosome may also render heterogeneity in the elongation rate. For example, mRNA secondary structure has been shown to cause translational pausing [23]. mRNA downstream of disome footprints exhibited a stronger secondary structure (Additional file 1: Fig. S15), indicating its role in inducing ribosome collisions. Relatedly, RNA helicases TIF1p and TIF2p were associated with disomes (Additional file 1: Table S4, S5), potentially removing mRNA secondary structures to relieve translational pauses. There are also sequence features that lead to ribosome collisions with unknown mechanisms. For example, two collision-inducing 3-mers, QQQ and GGG, were identified in the exit tunnel (Fig. 5b); they are not positively charged. Nevertheless, both are related to protein folding to some extent. The former is a well-known signal for protein misfolding [65], and the latter is a prominent signal for intrinsic protein disorder [66].

### The benefit from ribosome-collision mediated cotranslational protein folding

It is apparently costly when ribosomes are sequestered upstream of the stop codon, waiting for release, since they are not used for active protein synthesis. Nevertheless, slow ribosome release may benefit the cell by providing the newly synthesized peptide sufficient time to fold within the exit tunnel of ribosomes [67], rather than in the complex cytoplasmic environment. Some amino-acid sequences (e.g., proline and glycine at the P-site as well as polylysine in the exit tunnel) induce translational pauses as well (Figs. 4 and 5); they are also signals for the translation completion of α-helices (Fig. 4c, d). These amino-acid sequences may provide the time and subcellular environment for the folding of newly synthesized peptides inside the exit tunnel, especially α-helices. The narrowest region of the exit tunnel, the constriction site, is ~ 10 Å in width [68], sufficient for cotranslational folded α-helices (~ 5 Å in width) [69] to pass by. It would be important in the future to test if the manipulation of the translational pausing signals can affect protein folding.

Such a dual role of amino acids can catalyze the evolution of protein structure because it does not take additional time for placing a translational pausing signal after an evolutionary change in protein structure—the amino-acid substitution that results in innovation in protein structure at the same time confers a translational pause, making the emergent protein structure ready to be folded. Together with the cotranslational chaperones associated with collided ribosomes, the proper folding of a novel protein structure is warranted during evolution.

### The fates of ribosome collisions

The key to studying ribosome collision-mediated translational regulation is to unveil the dynamics of ribosomes during translation. Traditional ribo-seq provides the location information of monosomes but misses collided ribosomes, which are present in 46% of genes in yeast cells growing in the rich medium and provide unique information about translational pausing. Three similar studies in humans and zebrafish [38], yeast [56], and mice [57] were recently published during the preparation of our manuscript. All four studies, including our own, performed disome-seq and showed that ribosome collisions were widespread, despite the use of various model organisms.

We performed structural analyses on yeast disomes; 86.3% disomes were composed of two ribosomes that both were at the rotated state (Fig. 6a). This structure is consistent with the (28 + 2 + 28 =) 58-nt or the (28 + 5 + 28 =) 61-nt disome footprints that the same length of mRNA (28 nts) was protected by the two ribosomes. By contrast, when the leading ribosomes switch to the non-rotated state with P/P and E/E tRNAs, the two ribosomes will protect different lengths of mRNA (28 and 23 nts for the trailing and leading ribosomes, respectively). In mouse liver, Arpat et al. reported that the majority of disomes protected (29 + 1 + 29 =) 59 nts or (29 + 4 + 29 =) 62 nts [57]. Since the same length of mRNA (29 nts) was also protected by the two ribosomes, the lengths of these disome footprints can be explained by the double-rotated disome conformation determined in this study.

The data collected in the four studies led us to propose an integrated model for the ribosome-collision mediated translational regulation. Since an mRNA is translated simultaneously by multiple ribosomes, ribosome collisions are likely inevitable. The high ribosomal flux on an mRNA will increase the chance of collisions in general (Fig. 2d) due to the inherent stochasticity of biochemical reactions, on the basis of which sequence-associated heterogeneity in the elongation rate further promotes the propensity of collisions at specific locations. Besides, some ribosome collisions may be induced by aberrant transcripts or arrested nascent peptides.

There are at least three possible fates for these ribosome collisions observed in this study. Firstly, some ribosome collisions are likely transient, and the translation of both ribosomes can be resumed (Fig. 7e, top path); this could be the mechanism that keeps the ribosome density largely constant over a transcript (Fig. 3e, bottom panel, Additional file 1: Fig. S14c) in spite of widespread collided ribosomes (~ 5.8% at the moment that the disome “snapshot” was taken, Fig. 1b; or ~ 10% estimated in mouse liver [57]). Second, some ribosome collisions may last for a more extended period; these collisions may promote cotranslational protein folding or aid in targeting to correct subcellular locations [57, 70] (Fig. 7e, middle path). Third, if ribosome collisions last for a sufficiently long time, the leading ribosomes will shift to the non-rotated stalling state, and a more rigid interface between the collided ribosomes will trigger the recognition by the RQC pathway, leading to the degradation of mRNA and nascent peptides [9, 15, 16, 38, 56] (Fig. 7e, bottom path).

It is not yet clear the determinants of the collision duration, the mechanisms by which cells can sense how long a ribosome collision has persisted, and the fraction of ribosome collisions destined for each of the three fates. It is also unclear the mechanisms by which cells distinguish programmed (for protein folding or subcellular localization) and sporadic collisions (due to aberrant transcripts and defective nascent peptides). Considering the central role of ribosome collisions in protein homeostasis, these are key issues that need to be studied in the future.

## Conclusions

Ribosome collisions are widespread in fast proliferating yeast cells, especially on mRNA with high ribosomal flux. Ribosome collisions tend to occur at stop codons and are often related to the translation completion of α-helices. A large number of collided ribosomes are structurally incompetent to trigger the RQC pathway; instead, they are often associated with chaperones, which likely aid in cotranslational protein folding. Taken together, we offer a mechanism that chaperones sense translation elongation rate through ribosome collisions to determine which proteins/peptide regions require cotranslational folding.

## Methods

### Polysome profiling and disome-seq

#### Sample preparation

The laboratory strain BY4742 (*MATα his3Δ1 leu2Δ0 lys2Δ0 ura3Δ0*) was cultivated at 30 °C in the rich medium YPD (1% yeast extract, 2% peptone, and 2% dextrose). For experiments of the 3-AT treatment, strain S288C was cultivated at 30 °C in the SC−His+3-AT medium (synthetic complete medium with histidine dropped-out and 100 mM 3-AT added). We harvested cells following previous studies [24]. Briefly, cells were cultured for over two doubling cycles in the absence of cycloheximide, were collected at OD_660_ ~ 0.6 by vacuum filtration, and were immediately frozen in liquid nitrogen. Ribosomes were extracted with the polysome lysis buffer (PLB), which contained 200 mM Tris-HCl (pH 8.0), 200 mM KCl, 35 mM MgCl_2_, 1% (v/v) Triton X-100, 5 mM DTT, and 50 μg/mL cycloheximide.

#### Polysome profiling

The extracted ribosomes were pelleted through a 30 mL sucrose cushion containing 400 mM Tris-HCl (pH 8.0), 200 mM KCl, 30 mM MgCl_2_, 1.75 M sucrose, 5 mM DTT, and 50 μg/mL cycloheximide, by ultracentrifugation at 4 °C overnight (33,500 rpm, Beckman, 70Ti rotor). The ribosome pellet was dissolved in 300 μL RNase I buffer (20 mM Tris-HCl pH 8.0, 140 mM KCl, 5 mM MgCl_2_, 50 μg/mL cycloheximide, and 50 μg/mL chloramphenicol) and separated by ultracentrifugation at 4 °C for 3 h (35,300 rpm, Beckman, SW41 rotor) through a 5–50% sucrose gradient (40 mM Tris-HCl pH 8.4, 20 mM KCl, 10 mM MgCl_2_, and 50 μg/mL cycloheximide) prepared by Gradient Master (Biocomp). The profiling signals were recorded by Piston Gradient Fractionator (Biocomp).

#### Monosome-seq, disome-seq, and mRNA-seq

Libraries were prepared as described previously [17, 24, 55], with modifications. Fifty thousand units (A_260_) of ribosome dissolved in the PLB buffer were treated with 750 U RNase I (Ambion, AM2294) at 25 °C for 2 h. Our pilot experiments, as well as previous studies, have shown that sucrose gradient centrifugation is not necessary [55, 71–73] as long as the digestion with RNase I is complete (Additional file 1: Fig. S1); rather, this step consumes a large amount of ribosome (especially disome) samples. The following-up computational analyses can help to tell if the RNA fragments being collected are largely protected by ribosomes: whether the fragments are restricted to the coding sequences, whether the fragments are enriched in certain lengths, and whether a 3-nt periodicity exists (Additional file 1: Fig. S3c-f). Therefore, RNA was directly extracted from the solution for RNase I digestion with hot phenol and was separated on a 17% (w/v) 7 M urea denaturing polyacrylamide gel in a 0.5× Tris-borate-EDTA (TBE) electrophoresis buffer. RNA fragments with the length of approximate 20–30 nts or 50–80 nts were extracted by gel crushing and further incubated with an RNA gel extraction buffer (300 mM NaOAc pH 5.2, 10 mM Tris-HCl pH 8.0, 1 mM EDTA pH 8.0) overnight.

To control for technical bias during library preparation, appendant mRNA-seq was performed [17]. Specifically, total RNA was extracted using hot phenol, and 75 μg extracted RNA was applied to mRNA purification using the Dynabeads™ mRNA purification kit (Life Technologies, 61006). For fragmentation, 11.1 μL 10× fragmentation reagent (Thermo Fisher Scientific, AM8740) and 100 μL ddH_2_O were added to the purified mRNA. Fragmentation proceeded for 30 min at 70 °C and was aborted when 11.1 μL 10× stop solution was added. Fragmented RNA was precipitated by isopropanol overnight and was separated on a 17% (w/v) 7 M urea denaturing polyacrylamide gel. RNA fragments with the length ~ 28 nts were extracted from gel.

The extracted RNA fragments for monosome-seq, disomes-seq, or mRNA-seq were subjected to small RNA library construction for Illumina sequencing (Gnomegen, k02420). The 5′-RNA adaptor contained a 3-nt random sequence at the 3′-end to avoid potential ligation bias. Monosome-seq/RNA-seq and disome-seq libraries were sequenced with single-end 50 and paired-end 100 modes on BGISEQ-500 (BGI Group), respectively.

#### Mapping reads to the yeast genome

The 3-nt random sequence at the 5′-end of each sequencing read was removed. The removed 3-nt sequence was added to the head of each read in the fastq format as the UMI, which can serve to remove PCR duplications generated during Illumina library preparation. The sequence identical to the 3′-sequencing adaptor was also trimmed in each read using cutadapt V1.16 (http://gensoft.pasteur.fr/docs/cutadapt/1.6/index.html) [74]. Reads without the 3′-adaptor sequence were removed since they (> 46 nts for monosome-seq or > 96 nts for disome-seq) were much longer than the expectation (20–30 nts for monosome-seq and 50–80 nts for disome-seq). Trimmed reads shorter than 20 nts were also excluded from further analysis.

The *Saccharomyces cerevisiae* genome (SGD R64-1-1) (https://www.yeastgenome.org/) was used as the reference [75]. The trimmed reads were mapped against rRNA with bowtie V1.2.2 [76] (http://bowtie-bio.sourceforge.net/manual.shtml); the mapped reads were filtered to avoid rRNA contamination. The rest reads were aligned against coding sequences with the --no-novel-juncs parameter using Tophat V2.1.1 [77] (https://ccb.jhu.edu/software/tophat/manual.shtml). Reads with multiple alignments or with mapping quality < 30 were discarded. Biological replicates were highly correlated (Additional file 1: Fig. S2) and were combined in the majority of our analyses. To remove PCR duplicates generated during Illumina library preparation, sequencing reads of the same length, sequence, and UMI were counted only once. The number of disome reads obtained in this study (Additional file 1: Table S2) are comparable to those in previous studies [24].

#### The definition of the A-site

The definition of A-site in the disome footprints with various length is overviewed in Additional file 1: Fig. S16. Specifically, for the 58-nt disome footprints, if the 5′-end of a read was mapped to the first nucleotide of a codon (in frame), the 46th–48th nts was defined as the A-site of the leading ribosome; if mapped to + 1 (or + 2) frame, the 45th–47th (or 47th–49th) nts was defined as the A-site since the footprint likely shifted by + 1 and − 1 nt during RNase I digestion. The A-site of the leading ribosome was also defined for the 59-nt disome footprints: if the 5′-end was in frame, meaning that one more nt was kept at the 3′-end during RNase I digestion, the 46th–48th nts was the A-site. If mapped to + 2 frame, meaning that one more nt was kept at the 5′-end during RNase I digestion, the 47th–49th nts was the A-site. For the 61 and 62-nt disome footprints, the A-site was defined assuming that an extra codon was included in the space between two ribosomes. The A-site of the 53 and 54-nt disome footprints was defined as that of the 58 and 59-nt disome footprints since the footprints were trimmed from the 3′-end. For the 28 and 29-nt monosome footprints, the definition of the A-site was the same as the 58 and 59-nt disome footprints except for a 30-nt offset.

#### The calculation of the pausing scores

The A-site pausing score of each of the 64 codons was defined as the common odds ratio among genes calculated by the Mantel-Haenszel test. A 2 × 2 contingency table was generated for each gene. Disome footprints were divided into two categories, the concerning codon and other codons at the A-site of the leading ribosome. mRNA reads were used as the background to control for codon frequency in the gene; the “A-site” of a 28–29 nt mRNA-seq read was defined as if it was a 28–29 nt monosome footprint. The P-site pausing score was estimated similarly with the amino acid at the P-site under consideration. The exit-tunnel pausing score was estimated for each of the amino-acid 3-mers in the 20-amino-acid region upstream of the P-site; this length was conservative since peptides with 33 to 67 amino acids can be folded within the exit tunnel [78]. Flanking 3-mers may hitchhike on the causal 3-mer, and therefore, we combined multiple disome reads mapped to the same genomic position into one to reduce such a hitchhiking effect.

Statistical analyses were performed with *R* (https://www.r-project.org/), and plots were generated with an *R* package, ggplot2 (https://ggplot2.tidyverse.org/). The *Q* values were calculated using an *R* package, qvalue (http://github.com/jdstorey/qvalue). All statistical tests were two-sided unless otherwise specified.

### Dual-luciferase assays

The Renilla and firefly-luciferase sequences were obtained from the pTH727-CEN-RLuc/staCFLuc plasmid (http://www.addgene.org) and were concatenated with a linker sequence GGTCGACGGATCCCCGGG between them, for the purpose of keeping correct protein folding. This Renilla-linker-firefly fragment was further inserted into the p426 plasmid and was expressed under *TDH3* promoter. The sequences of AAGAAGAAG, AAGAAGAAA, AAGAAAAAG, AAAAAGAAG, AAGAAAAAA, AAAAAGAAA, AAAAAAAAG, and AAAAAAAAA were individually inserted upstream of the coding sequence of firefly luciferase. Individual plasmids were transformed into BY4742. The plasmid-containing yeast clones were selected on SC–uracil plates.

The activity of luciferase was detected as described in a previous study [79]. Briefly, transgenic yeast strains were individually cultivated in the SC–uracil medium (150 μL) at 30 °C overnight. Ten microliters of each yeast culture was transferred into a fresh SC–uracil medium (140 μL) and was cultivated for another 4 h in a 96-well plate. Passive lysis buffer (40 μL, Promega, E1910) was added per well for cell lysis. Twenty-five microliters of the suspension was mixed with 25 μL firefly luciferase substrate (Promega, E1910) and was incubated for 20 min at 25 °C. Firefly luciferase activity was measured by the Synergy multi-mode reader (BioTek). Twenty-five microliters of Stop-and-Glo reagent (Promega, E1910) was added and mixed, which was incubated for another 20 min before the measurement of Renilla-luciferase activity.

### Cryo-EM

#### Disome purification

Ten thousand units (A_260_) of ribosome pellet were purified from cell lysates (cycloheximide omitted) and were subjected to digestion in 300 μL RNase I buffer with 200 U RNase I (Ambion, AM2294) at 25 °C for 2 h. The disome fraction was collected with sucrose gradient centrifugation and was concentrated through a 50-kDa centrifugal filter (Millipore, UFC905096). The concentration was adjusted to *A*_260_ = 11.0.

#### Data acquisition

Vitrified specimens were prepared by adding 4 μL disome samples at the concentration of ~ 150 nM to a glow-discharged holey carbon grid (Quantifoil R1.2/1.3), which was covered with a freshly made thin carbon film. Grids were blotted for 1 s and plunge-frozen into liquid ethane using an FEI Vitrobot Mark IV (4 °C and 100% humidity). The cryo-grids were initially screened at a nominal magnification of × 92,000 in an FEI Talos Arctica microscope (200 kV), equipped with an FEI CETA camera. High-quality grids were transferred to an FEI Titan Krios electron microscope operating at 300 kV, and images were collected using a K2 Summit direct electron detector (Gatan) in counting mode at a nominal magnification of × 105,000, which corresponds to a pixel size of 1.356 Å at the object scale (with defocus varying from − 1.0 to − 2.0 μm). Movie stacks were collected semi-automatically using SerialEM [80]. Each micrograph was dose-fractionated to 32 frames with a dose rate of ~ 10.0 counts per physical pixel per second for a total exposure time of 6.4 s.

#### Data processing

Original image stacks were summed and corrected for drift and beam-induced motion at the micrograph level using MotionCor2 [81]. SPIDER [82] was used for micrograph screening. The contrast transfer function parameters of each micrograph were estimated by Gctf [83]. All 2D and 3D classification and refinement were performed with RELION-3.0 [84].

The disome data were initially processed using 80S monosome as a template for particle picking. A total of 1683 micrographs were collected, and 355,439 particles were picked for a cascade 2D and 3D classification with a binning factor of two. 281,953 particles with a box size of 400 pixels were split into 10 classes during the first round 3D classification (Additional file 1: Fig. S17).

Particles contained densities for two 80S ribosomes were re-centered and re-extracted with an enlarged box size of 440 pixels. After removing duplicated particles, 45,119 disome particles were subjected to another round of 3D classification. Two classes (a total of 35,918 particles) were pooled, and a soft mask on the leading ribosome was applied during the following mask-based 3D classification. Two groups were obtained: 86.3% of the particles contained A/P and P/E tRNA in the leading ribosome (group 1), the rest (13.7%) containing P/P and E/E tRNA in the leading ribosome (group 2). The 4908 particles in group 2 were subjected to 3D auto-refine with a soft mask on the trailing or leading ribosome, resulting in 7.96 Å or 8.06 Å maps, respectively. The 31,010 particles in group 1 were split into ten classes during the final round of 3D classification with a soft mask on the trailing ribosome. A total of 27,599 particles were subjected to 3D auto-refine with a soft mask on the trailing or leading ribosome, resulting in 4.85 Å or 4.59 Å maps (gold-standard FSC 0.143 criteria), respectively (Additional file 1: Fig. S17, S18).

#### The structural determination of disomes collected in the cycloheximide-containing lysis buffer

Disomes were also collected in the lysis buffer containing 50 μg/mL cycloheximide. The experimental and computational procedures were otherwise similarly performed. In brief, a total of 1439 micrographs were collected, and 269,962 particles were picked for a cascade 2D and 3D classification. A total of 4194 particles were applied for 3D auto-refine with a soft mask on the trailing or leading ribosome, resulting in 7.11 Å or 8.39 Å map, respectively.

### Mass spectrometry

Stable isotope labeling with amino acids in cell culture (SILAC) was performed as described previously [85]. Briefly, a strain modified from BY4742 (*MATα his3Δ1 leu2Δ0 lys2Δ0 ura3Δ0 arg4Δ0::kanMX4 car1Δ0::LEU2*) was cultured at 30 °C in the regular SC medium or in the SC medium substituted with heavy isotopes (37.25 mg/L Lys8 and 20.94 mg/L Arg10). Lys8 and Arg10 represent ^15^N_2_^13^C_6_-lysine and ^15^N_4_^13^C_6_-arginine, respectively.

Cells were harvested at the mid-log phase (OD_660_ ~ 0.6), and polysomes were extracted. Polysome profiling was performed after RNase I digestion, and proteins were precipitated from the monosome or disome fraction with a double volume of 95% ethanol, respectively. The protein precipitants were dissolved with the urea buffer (8 M urea, 1 mM sodium orthovanadate, 1 mM sodium fluoride, 2.5 mM sodium pyrophosphate, 1 mM B-glycerophosphate, 0.2% tablet of protease inhibitors, and 1 mM PMSF). One hundred fifty micrograms of light-labeled monosome protein was mixed with 150 μg heavy-labeled disome protein; 150 μg heavy-labeled monosome protein was mixed with 150 μg light-labeled disome protein as a replicate. The protein mixtures were subject to the MS/MS analysis on Orbitrap Elite (Thermo Fisher Scientific).

The raw data were processed with MaxQuant V1.5.8.3 [86] (https://www.biochem.mpg.de/5111795/maxquant) using default parameters. All raw data were searched against the yeast proteome with the addition of potential contaminants. The protease was set as trypsin/P and trypsin. Two missed cuts were allowed. The protein intensities were retrieved from the output file (proteinGroups.txt) of MaxQuant. The proteins associated with ribosome collision can be identified by comparing the abundance per ribosome between disomes and monosomes. In principle, if a significant proportion of monosomes collected in the experiments were vacant, this comparison will identify proteins associated with translating ribosomes instead of those associated with disomes. However, this is an unlikely case since all 197,367 monosomes detected in our cryo-EM analyses accommodated tRNAs, indicating they are mostly translating ribosomes.

If the peptides of metabolic enzymes were detected in the ribosome particles because they were nascent peptides of incomplete protein translation, they should be enriched in the first half (N-terminus) of a protein. To determine if it is true, we divided the peptides detected into two categories, according to whether the N-terminus of the peptide belonged to the first half of the protein or not. With the theoretical digested peptides (arginine or lysine at the C-terminus) as a background, a 2 × 2 contingency table was generated for each gene. The common odds ratio among genes was calculated by the Mantel-Haenszel test.

### Immunoblotting

We did not have specific antibodies for the yeast chaperones enriched in disomes; therefore, we decided to perform immunoblotting for 4×FLAG tagged chaperones with the antibody against the FLAG tag. To this end, we constructed transgenic yeast lines where 4×FLAG tag was inserted to the C-terminus of five chaperones. Specifically, a DNA fragment composing the terminator of *ADH1* and the *kanMX* cassette was obtained from the pBS7 plasmid. 4×FLAG sequence was fused to the 5′-terminus of this fragment, which was further cloned into the pUC57 plasmid to generate the pUC57-*4×FLAG*-*ADH1 terminator*-*kanMX* plasmid (TSINGKE Biological Technology). Primers were designed to amplify this fragment and for recombination-based knock-in, right before the stop codon of each chaperone gene (Additional file 1: Table S6).

Proteins were isolated from the monosome or disome fraction as described in the “Mass spectrometry” section. An equal amount of monosome and disome proteins were separated by 10% SDS-polyacrylamide gel and were transferred on to a Hybond ECL (GE Healthcare, RPN303D) membrane. The membrane was probed with anti-DDDDK tag (binding to FLAG® tag sequence, horseradish peroxidase-conjugated; Abcam, ab49763). Chemiluminescent horseradish peroxidase substrate (Millipore, WBKLS0100) was added to detect the antibody. The signal was captured with e-BLOT TOUCH IMAGER (Beijing Poliness Trade Co., Limited).

### Computational analyses of the disome-seq data for human cells

The human genome (Ensembl Genes 101) was downloaded from Ensembl [87]. The most reliable principal isoform was used as the reference cDNA for each gene. The disome-seq data for human cells were retrieved from the Gene Expression Omnibus (GEO) at the National Center for Biotechnology Information (NCBI) under the accession number of GSE145723. The sequencing reads were mapped against human rRNAs with bowtie V1.2.2, and the unmapped reads were used in the following analyses. These reads were aligned against human protein-coding transcripts using STAR V2.7.3a [88] with the following parameters, --alignEndsType EndToEnd, --outFilterType BySJout, --outFilterMismatchNmax 2, --outFilterMultimapNmax 5, --outFilterMatchNmin 16. We followed the previous definition for the A-site of the leading ribosome in a disome footprint [38]. Specifically, offsets were 45 nts for the 51-nt and 59-nt footprints, 46 nts for the 52-nt and 60-nt footprints, 48 nts for the 54-nt and 62-nt footprints, and 49 nts for the 55-nt and 63-nt footprints.

### The annotation of α-helices

The protein secondary structure annotation of yeast protein was retrieved from UniProt [89] (https://www.uniprot.org/). Adjacent α-helices without a gap were concatenated. Short α-helices composed of 4 amino acids or less were filtered. The secondary structure of Rps31p was predicted with the online secondary structure prediction method, GOR IV [90], at https://npsa-prabi.ibcp.fr/.

### Alignment of proteins from fungi, animals, and plants

Homologous genes were identified by BLAST (https://blast.ncbi.nlm.nih.gov/Blast.cgi). Orthologous gene pairs between yeast and human were retrieved from Ensembl BioMart [91] and NCBI HomoloGene [92]. Protein sequences were aligned by Clustal X [93]. Species trees were constructed with TimeTree [94] at http://www.timetree.org.

### Prediction of the minimum free energy of an mRNA fragment

The minimum free energy of the 30-nt mRNA downstream of a disome footprint was calculated with the RNAfold V2.4.13 of the Vienna RNA package [95] (http://rna.tbi.univie.ac.at/).

## Supplementary Information

**Additional file 1: Fig. S1.** Disomes persisted after RNase I digestion. **Fig. S2.** Correlations between libraries of disome-seq, monosome-seq, and mRNA-seq. **Fig. S3.** The distribution of monosome and disome footprints. **Fig. S4.** The A-site pausing scores between replicates or between data processing approaches. **Fig. S5.** Highly translated mRNAs are crowded with ribosomes. **Fig. S6.** The contact interfaces of disomes (cycloheximide omitted in the lysate) and di-ribosomes. **Fig. S7.** The identification of disome-associated proteins. **Fig. S8.** Ribosome footprints in the two-ribosome-containing transcripts. **Fig. S9.** The detection of the 5′-ligation bias during the library preparation for monosome-seq and disome-seq. **Fig. S10.** The pausing scores estimated using the codon/amino-acid frequency in the genome as the background. **Fig. S11.** Additional cryo-EM structures of disomes. **Fig. S12.** On the 61-nt disome footprints. **Fig. S13.** On the 53-nt disome footprints. **Fig. S14.** The “inverse ramp” of disome footprints on the CDS. **Fig. S15.** The mRNA secondary structure downstream of disome footprints. **Fig. S16.** The presumed conformation and the putative A-site for disome footprints of various lengths **Fig. S17.** Cryo-EM data processing for disome particles that were collected with cycloheximide omitted in the lysis buffer. **Fig. S18.** Fourier shell correlation (FSC) curves for the final 3D density maps after RELION-based post-processing. **Table S1.** Summary of the monosome-seq libraries. **Table S2.** Summary of the disome-seq libraries. **Table S3.** Summary of the mRNA-seq libraries. **Table S4.** Disome-associated proteins (replicate 1: heavy isotope labeled disome proteins, disome/monosome intensity ratio > 1.5). **Table S5.** Disome-associated proteins (replicate 2: light isotope labeled disome proteins, disome/monosome intensity ratio > 1.5). **Table S6.** Primers used for tagging 4×FLAG to the C-terminus of each chaperone.

**Additional file 2.** Review history.

## Data Availability

The *S. cerevisiae* genome (SGD R64-1-1) was downloaded from https://www.yeastgenome.org/ [75]. The protein secondary structure annotation of yeast protein was retrieved from UniProt (https://www.uniprot.org/) [89]. The human genome (Ensembl Genes 101) was retrieved from Ensembl [87]. Orthologous gene pairs between yeast and human were retrieved from Ensembl BioMarts [91] and NCBI HomoloGene [92]. The high-throughput sequencing data of disome-seq, monosome-seq, and mRNA-seq discussed in this publication have been deposited in NCBI GEO [96] and are accessible through GEO Series accession number GSE158572 [97]. The mass spectrometry proteomics data have been deposited to the ProteomeXchange Consortium (http://proteomecentral.proteomexchange.org) via the PRIDE partner repository [98] with the dataset identifier PXD015271 [99]. The cryo-EM density maps have been deposited in the Electron Microscopy Data Bank (EMDB) (http://www.emdataresource.org/) [100] under accession numbers of EMD-30553 [101] and EMD-30554 [102] (the leading and trailing ribosomes of disome collected without cycloheximide in the lysis buffer) and EMD-30580 [103] and EMD-30581 [104] (the leading and trailing ribosomes of disome collected with cycloheximide in the lysis buffer). Codes to analyze the data are available at GitHub, under the terms of the MIT license [105], and at Zenodo [106].
