# Supplementary material for: Disome-seq reveals widespread ribosome collisions that promote cotranslational protein folding
Source: Genome Biol. 2021 Jan 5;22:16. doi: 10.1186/s13059-020-02256-0 (PMC7784341; doi:10.1186/s13059-020-02256-0)
Supplement: Supplementary file 1 — Additional file 1: Fig. S1. Disomes persisted after RNase I digestion. Fig. S2. Correlations between libraries of disome-seq, monosome-seq, and mRNA-seq. Fig. S3. The distribution of monosome and disome footprints. Fig. S4. The A-site pausing scores between replicates or between data processing approaches. Fig. S5. Highly translated mRNAs are crowded with ribosomes. Fig. S6. The contact interfaces of disomes (cycloheximide omitted in the lysate) and di-ribosomes. Fig. S7. The identification of disome-associated proteins. Fig. S8. Ribosome footprints in the two-ribosome-containing transcripts. Fig. S9. The detection of the 5′-ligation bias during the library preparation for monosome-seq and disome-seq. Fig. S10. The pausing scores estimated using the codon/amino-acid frequency in the genome as the background. Fig. S11. Additional cryo-EM structures of disomes. Fig. S12. On the 61-nt disome footprints. Fig. S13. On the 53-nt disome footprints. Fig. S14. The “inverse ramp” of disome footprints on the CDS. Fig. S15. The mRNA secondary structure downstream of disome footprints. Fig. S16. The presumed conformation and the putative A-site for disome footprints of various lengths Fig. S17. Cryo-EM data processing for disome particles that were collected with cycloheximide omitted in the lysis buffer. Fig. S18. Fourier shell correlation (FSC) curves for the final 3D density maps after RELION-based post-processing. Table S1. Summary of the monosome-seq libraries. Table S2. Summary of the disome-seq libraries. Table S3. Summary of the mRNA-seq libraries. Table S4. Disome-associated proteins (replicate 1: heavy isotope labeled disome proteins, disome/monosome intensity ratio > 1.5). Table S5. Disome-associated proteins (replicate 2: light isotope labeled disome proteins, disome/monosome intensity ratio > 1.5). Table S6. Primers used for tagging 4×FLAG to the C-terminus of each chaperone. [file 13059_2020_2256_MOESM1_ESM.docx]

Supplementary Information For

**Disome-seq reveals widespread ribosome collisions that
promote cotranslational protein folding**

T. Zhao, Y.-M. Chen, Y. Li, J. Wang, S. Chen, N. Gao, and W. Qian

Supplementary information includes Supplementary Figures S1-18 and Supplementary Tables S1-6.

**SUPPLEMENTARY FIGURES**


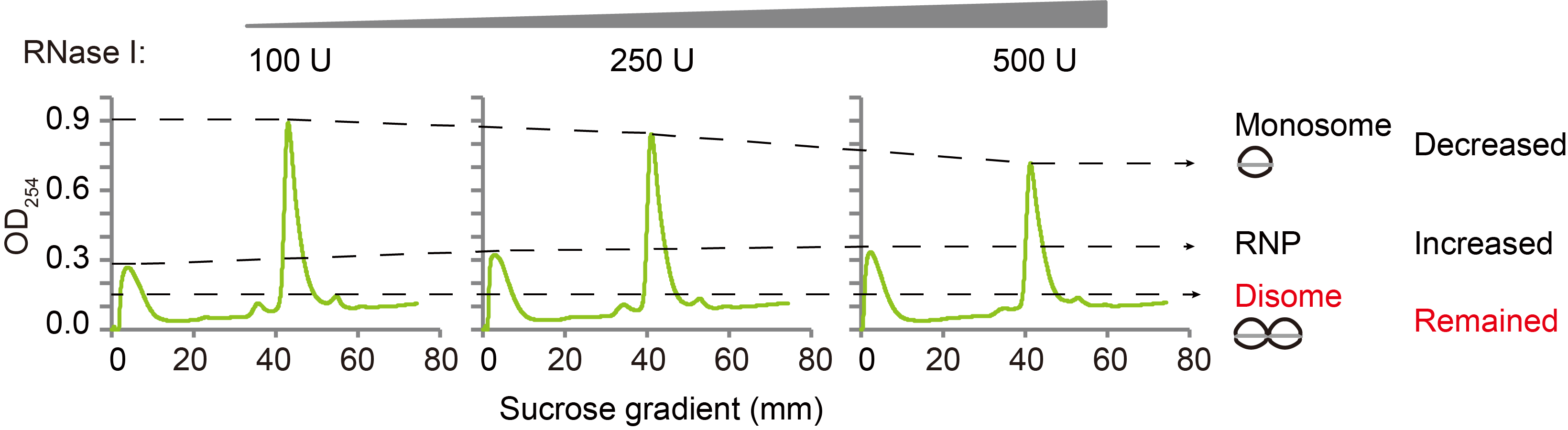


**Fig. S1. Disomes persisted after RNase I digestion.**

Samples containing an equal amount of ribosome-bound mRNA (5000 A_260_ unit) were treated with 100 U, 250 U, and 500 U RNase I, respectively. As the concentration of RNase I increased, the abundance of monosome reduced, and that of free ribonucleoprotein (RNP) increased, suggesting the disruption of ribosomes by the excessive RNase I digestion. However, the disome persisted—the mRNA fragment in-between was resistant to the RNase I digestion likely due to the steric effect.


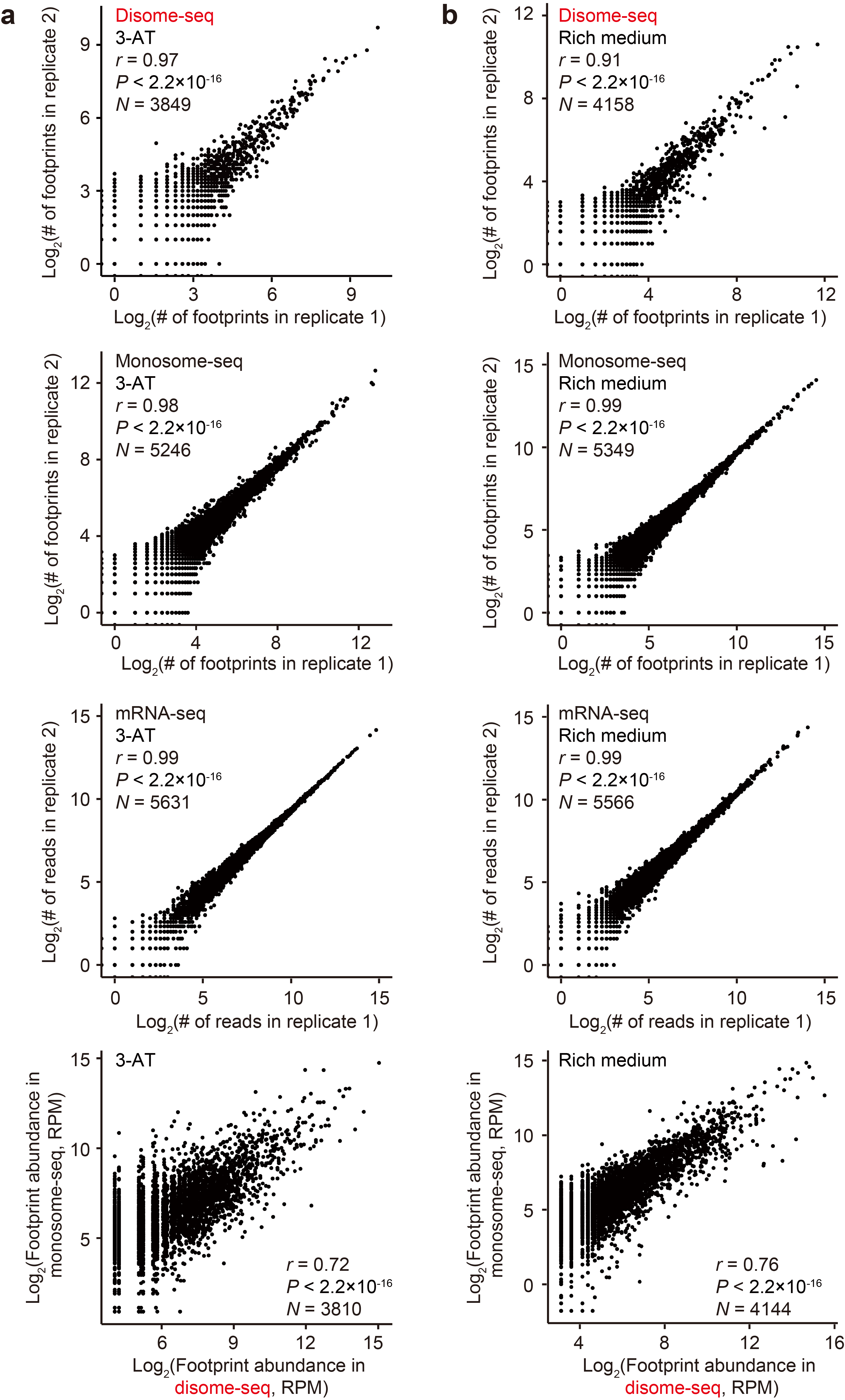


**Fig. S2. Correlations between libraries of disome-seq, monosome-seq, and mRNA-seq.**

**a** The top three scatter plots show the number of mapped reads for each gene between biological replicates. The bottom scatter plot shows the abundance of mapped reads (in the unit of reads per million) for each gene between disome-seq and monosome-seq; the average footprint abundance of two biological replicates is shown. All libraries were obtained from 3-AT treated yeast cells. Each dot represents a gene, and the *P*-values were given by Pearson’s correlation.

**b** Same as (**a**), except yeast cells were cultured in the rich medium.


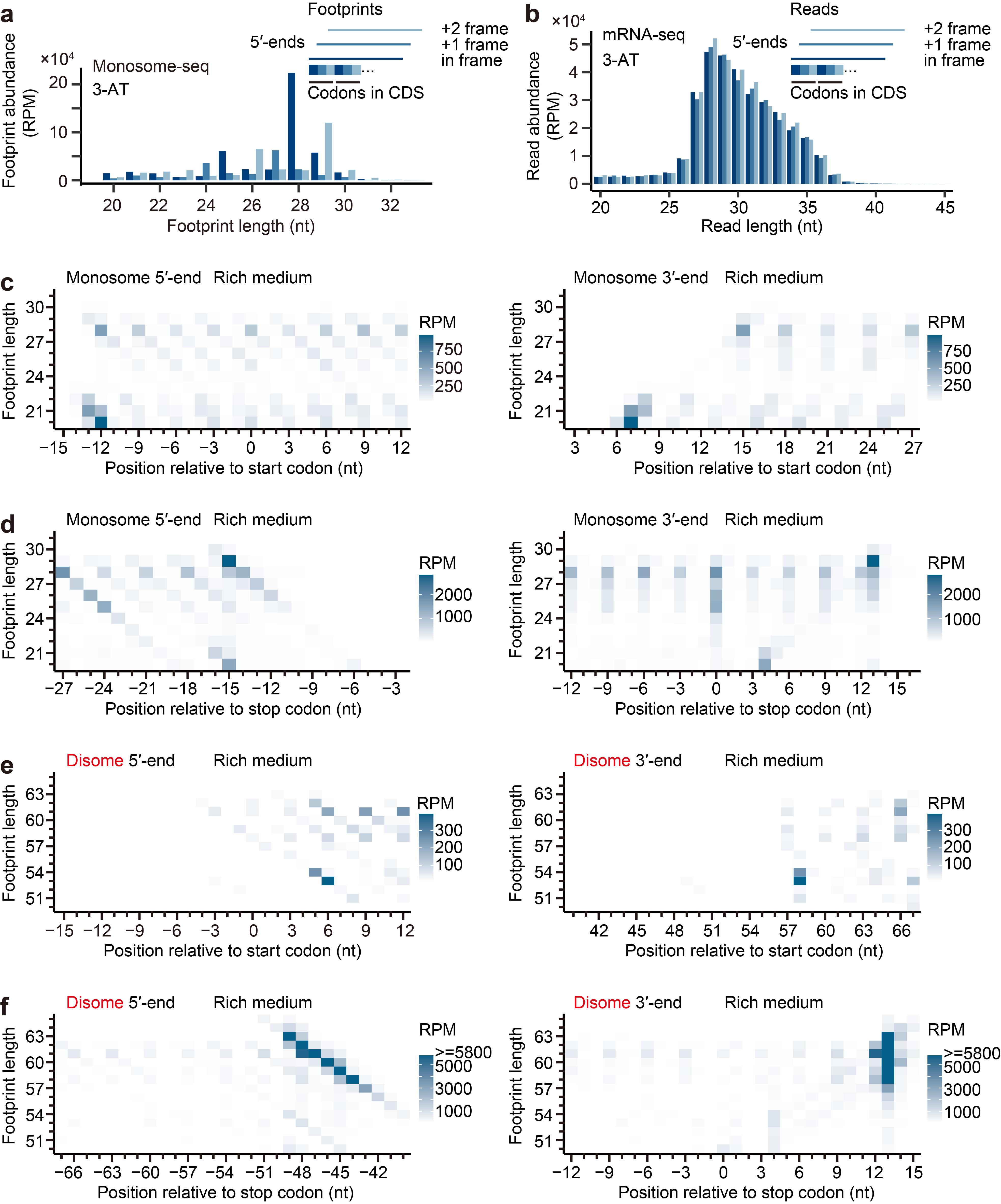


**Fig. S3. The distribution of monosome and disome footprints.**

**a** The length distribution of monosome footprints obtained from 3-AT treated yeast cells. The 5′-end of the 28-nt monosome footprints displayed an apparent 3-nt periodicity, similar to previous observations by Ingolia *et al*. (2009). It is consistent with the non-overlapping 3-nt genetic code – ribosome moves three nts per work cycle of translation elongation. The average footprint abundance of two biological replicates is shown.

**b** The length distribution of mRNA-seq reads obtained from 3-AT treated yeast cells. The mRNA-seq reads display no apparent periodicity. The average read abundance of two biological replicates is shown.

**c, d** The monosome footprints obtained from yeast cells growing in the rich medium. The aggregated abundance profile around the start codon (**c**) and the stop codon (**d**). The 5′-end (left) or the 3′-end (right) positions of the footprints are plotted against the footprint length. The color scale reflects the abundance of footprints. As shown in the plot, the two major footprint lengths, 28-nt and 20-nt, share the same 5′-end but different 3′-end. As reported in a previous study (Wu *et al*., 2019), the short footprints (~21-nt) are detected when the A-site of a ribosome is open whereas the long footprints (~28-nt) are detected when the A-site of a ribosome is occupied. More 20-nt monosome footprints were observed around the start codon, likely because the A-site of ribosomes is open during translation initiation; more 28-nt monosome footprints were observed around the stop codon, likely because the A-site of ribosomes is rapidly occupied by the release factor.

**e, f** Similar to (**c, d**), for disome footprints.


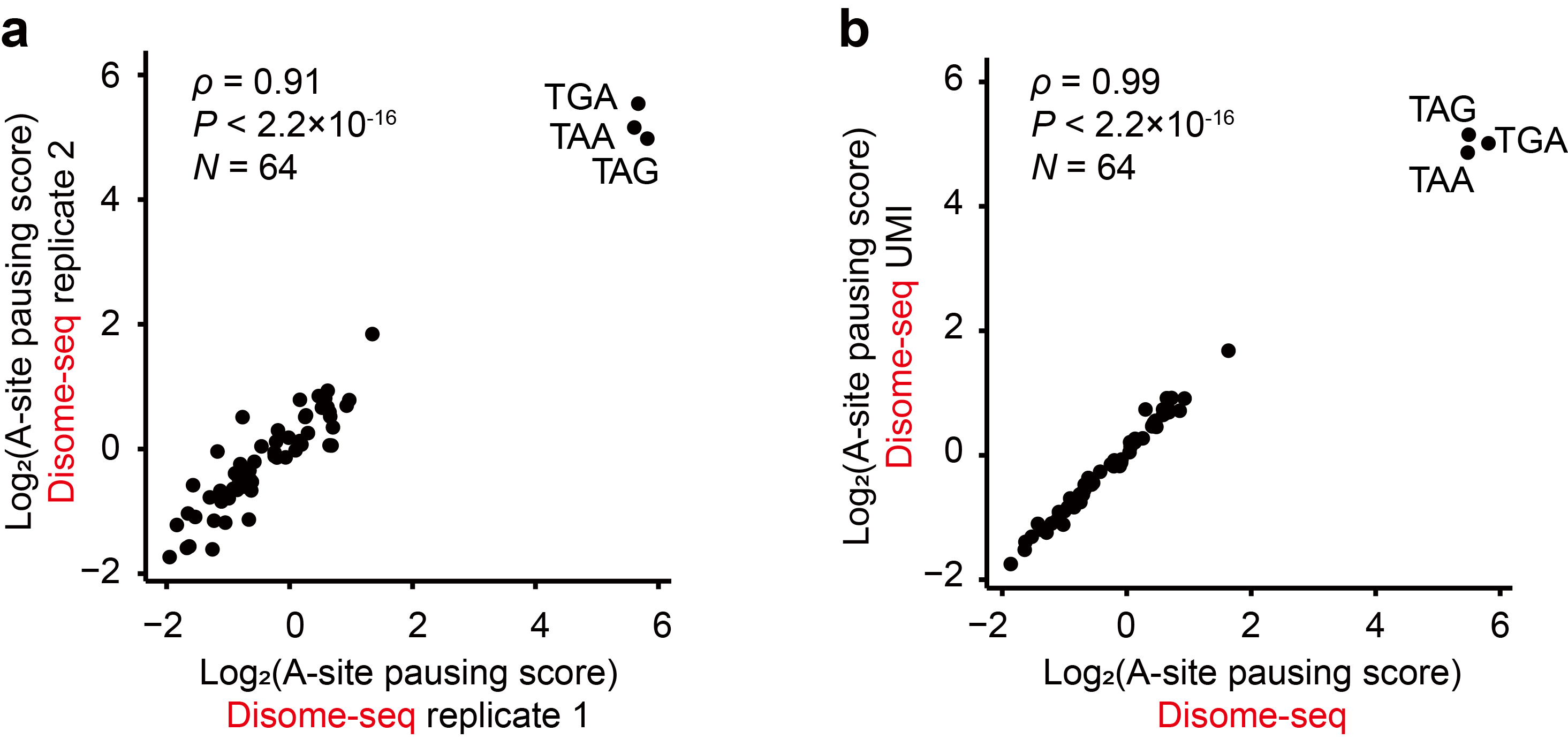


**Fig. S4. The A-site pausing scores between replicates or between data processing approaches.**

**a** The scatter plot shows the A-site pausing scores for disome footprints calculated from two biological replicates.

**b** The scatter plot shows the A-site pausing scores for disome footprints calculated before and after PCR duplicates were removed using the UMI information.


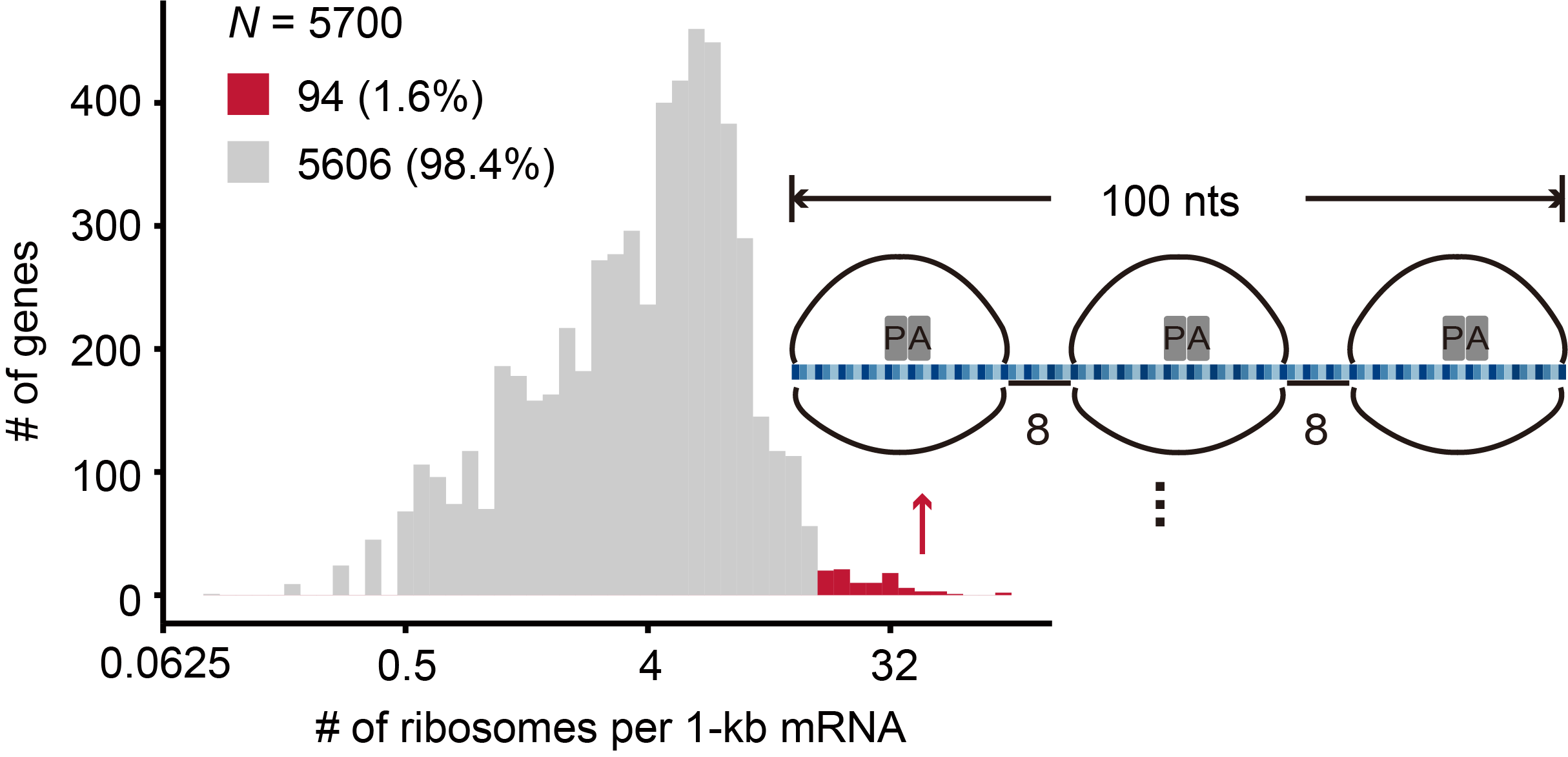


**Fig. S5. Highly translated mRNAs are crowded with ribosomes.**

Data were retrieved from Arava *et al.* (2003).


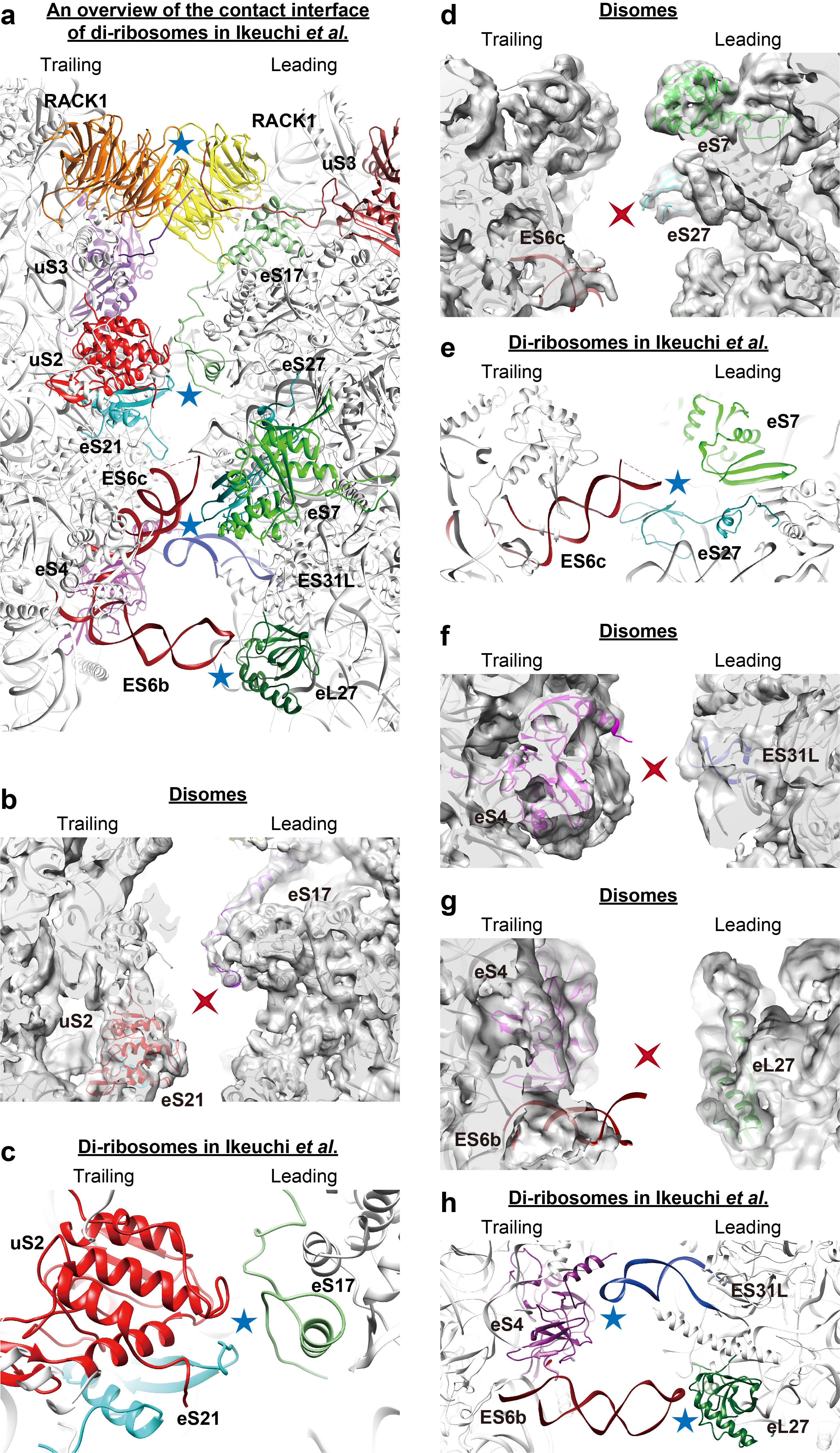


**Fig. S6. The contact interfaces of disomes (cycloheximide omitted in the lysate) and di-ribosomes.**

**a** The zoomed-in overview of the contact interface between the 40S subunits of the RQC-inducing di-ribosomes (Ikeuchi *et al*., 2019).

**b**, **c** The zoomed-in view of the 40S body-to-body contact site of disomes (**b**) and di-ribosomes (**c**). The interactions between eS17 of the leading ribosome and the uS2 and eS21 of the trailing ribosome were observed in the di-ribosome, but were absent in the disome.

**d**, **e** The zoomed-in view of the 40S platform-to-platform contact site of disomes (**d**) and di-ribosomes (**e**). The interactions between eS27, eS7 of the leading ribosome and the expansion segment ES6c of the trailing ribosome were observed in the di-ribosome, but were absent in the disome.

**f-h** The zoomed-in view of the 60S-to-40S contact site of disomes (**f**, **g**) and di-ribosomes (**h**). The dual interactions between ES31L of the leading ribosome and eS4 of the trailing ribosome (**f, h**) and between eL27 of the leading ribosome and ES6b of the trailing ribosome (**g, h**) were observed in the di-ribosome, but were absent in the disome.

The blue stars indicate strong interactions between the two ribosomes in the di-ribosome. The red crosses indicate that these interactions are absent in the disome.


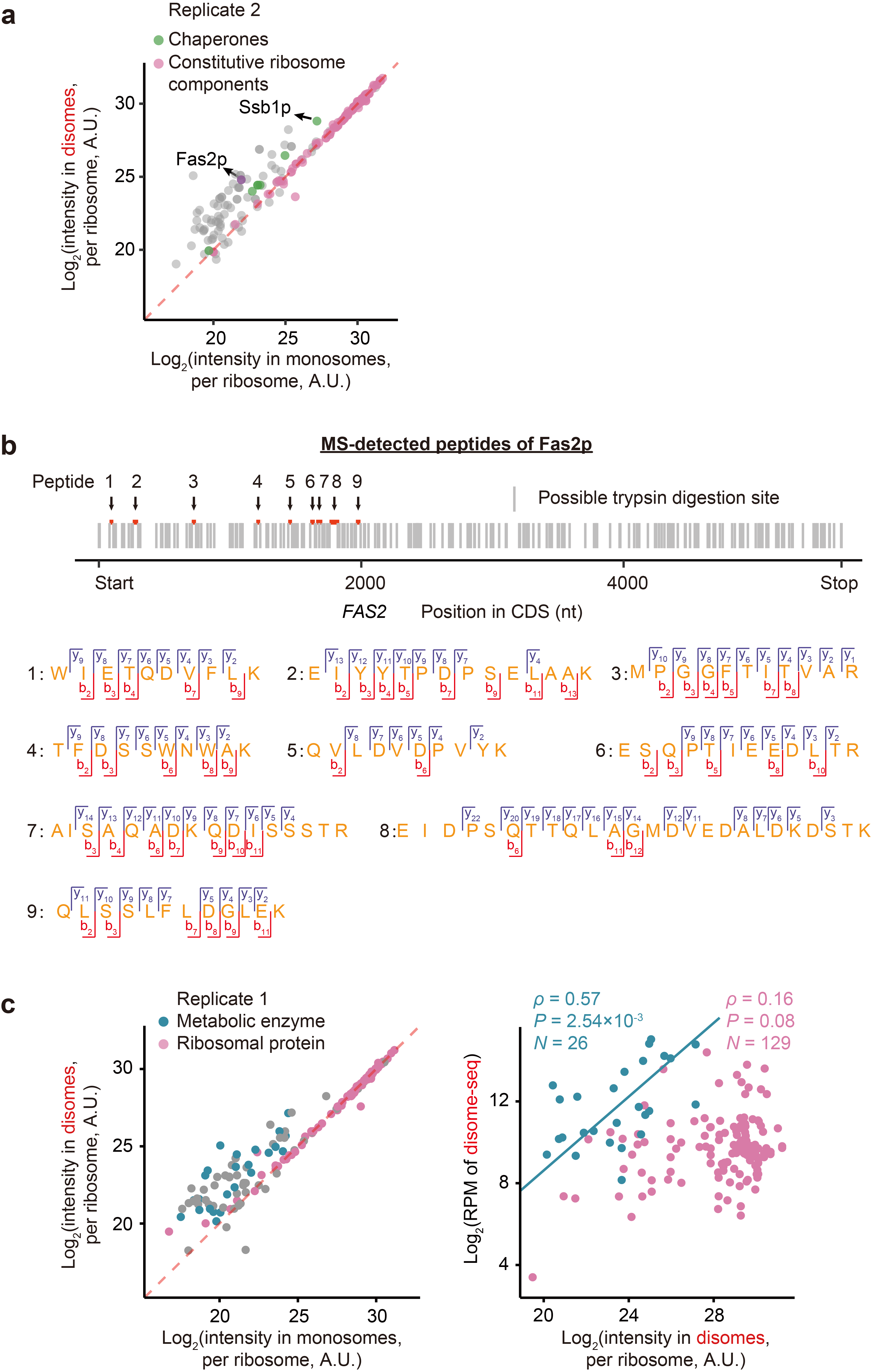


**Fig. S7. The identification of disome-associated proteins.**

**a** A biological replicate of **Fig. 7b**. Fas2p is highlighted in purple.

**b** MS-detected peptides (red) and the N-termini of the theoretical peptides generated by *in silico* trypsin digestion (grey) of Fas2p. MS/MS spectra are shown at the bottom.

**c** Positive correlation between the abundance of disome footprints (detected by disome-seq) and the protein intensity detected in the disome fraction (detected by MS/MS) among metabolic enzymes. As a negative control, no such correlation was detected among ribosomal proteins. The blue line shows the major axis among metabolic enzymes.


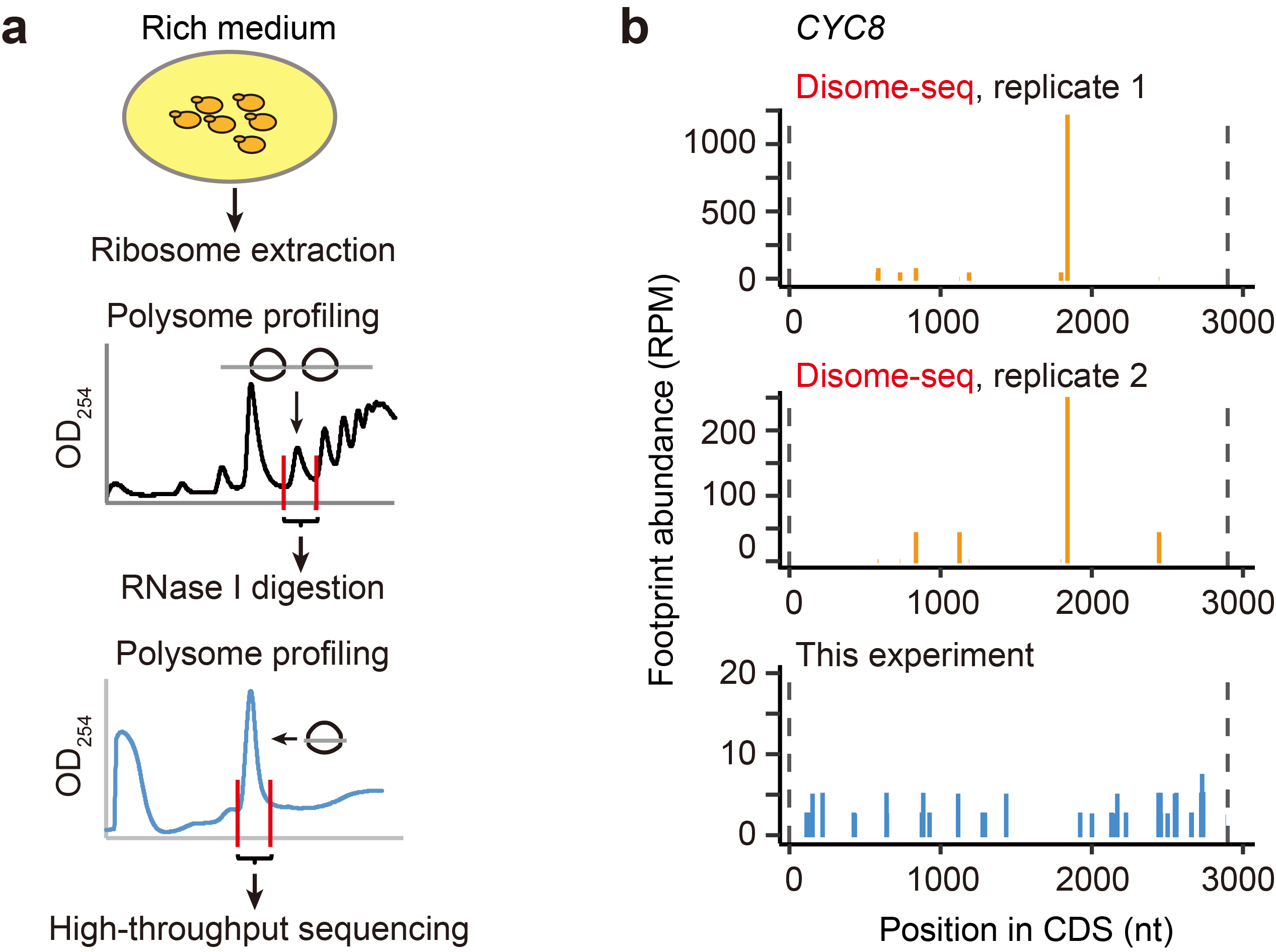


**Fig. S8. Ribosome footprints in the two-ribosome-containing transcripts.**

**a** The ribosome footprints in the transcripts bound by two ribosomes were sequenced.

**b** The ribosome collision that was repeatedly detected in disome-seq (orange) was not observed in this experiment (blue).


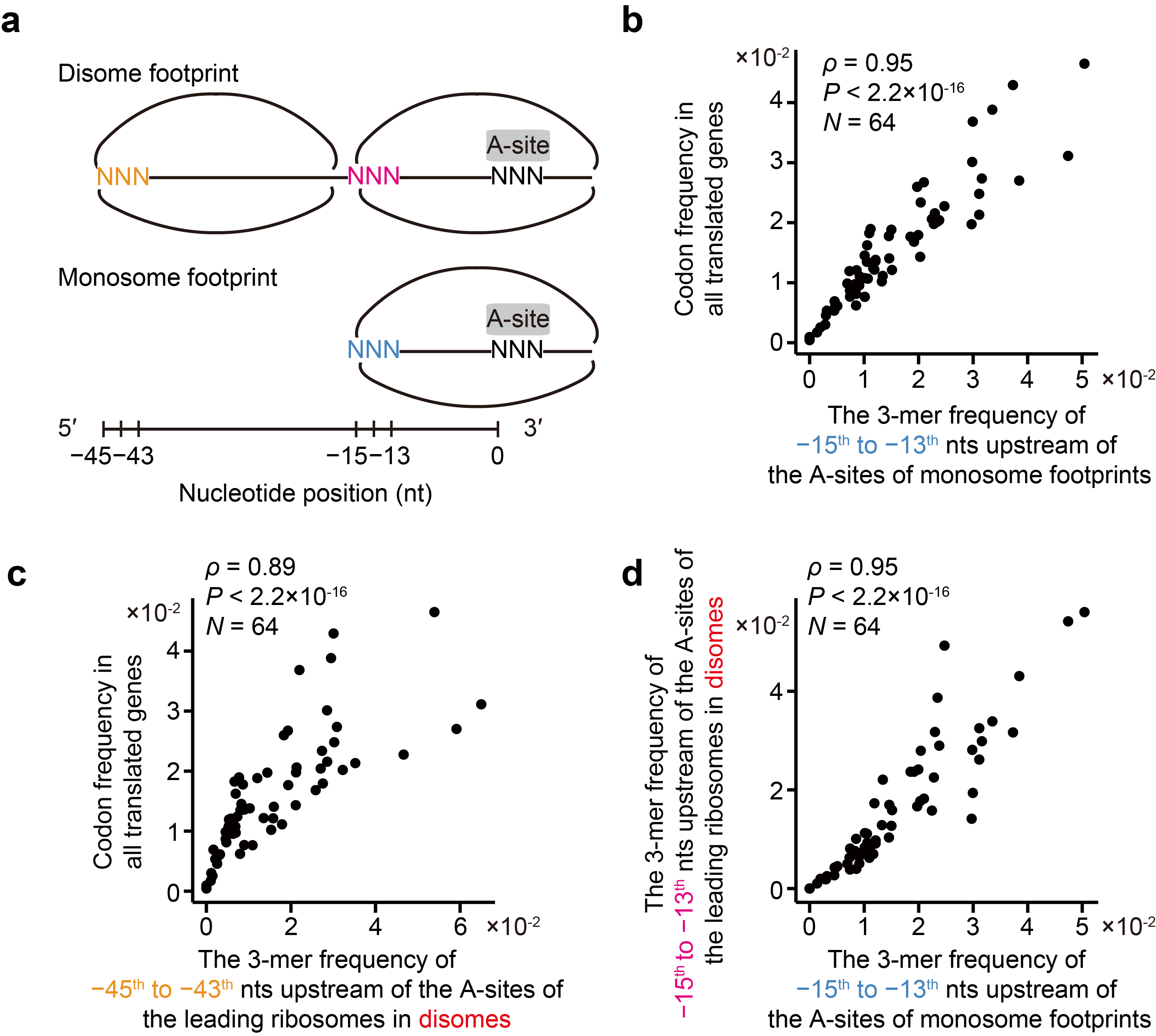


**Fig. S9. The detection of the 5′-ligation bias during the library preparation for monosome-seq and disome-seq.**

**a** The schematic of the nucleotide position of the 3-mers used in this figure.

**b** The correlation between the codon frequency in all translated genes and the 3-mer frequencies in the −15^th^ to −13^th^ nt region upstream of the A-sites of the monosome footprints. The codon frequency was used (instead of the 3-mer frequency in all three reading frames) because the −15^th^ to −13^th^ nt (as well as the −45^th^ to −43^th^ nt) region upstream of an A-site should be in frame.

**c** Similar to (**b**), but the −45^th^ to −43^th^ nt region upstream of the A-sites of the leading ribosomes detected in disome-seq.

**d** The correlation of the frequency of 3-mers in the −15^th^ to −13^th^ nt region upstream of the A-sites of the (leading) ribosomes detected in monosome-seq (or disome-seq).


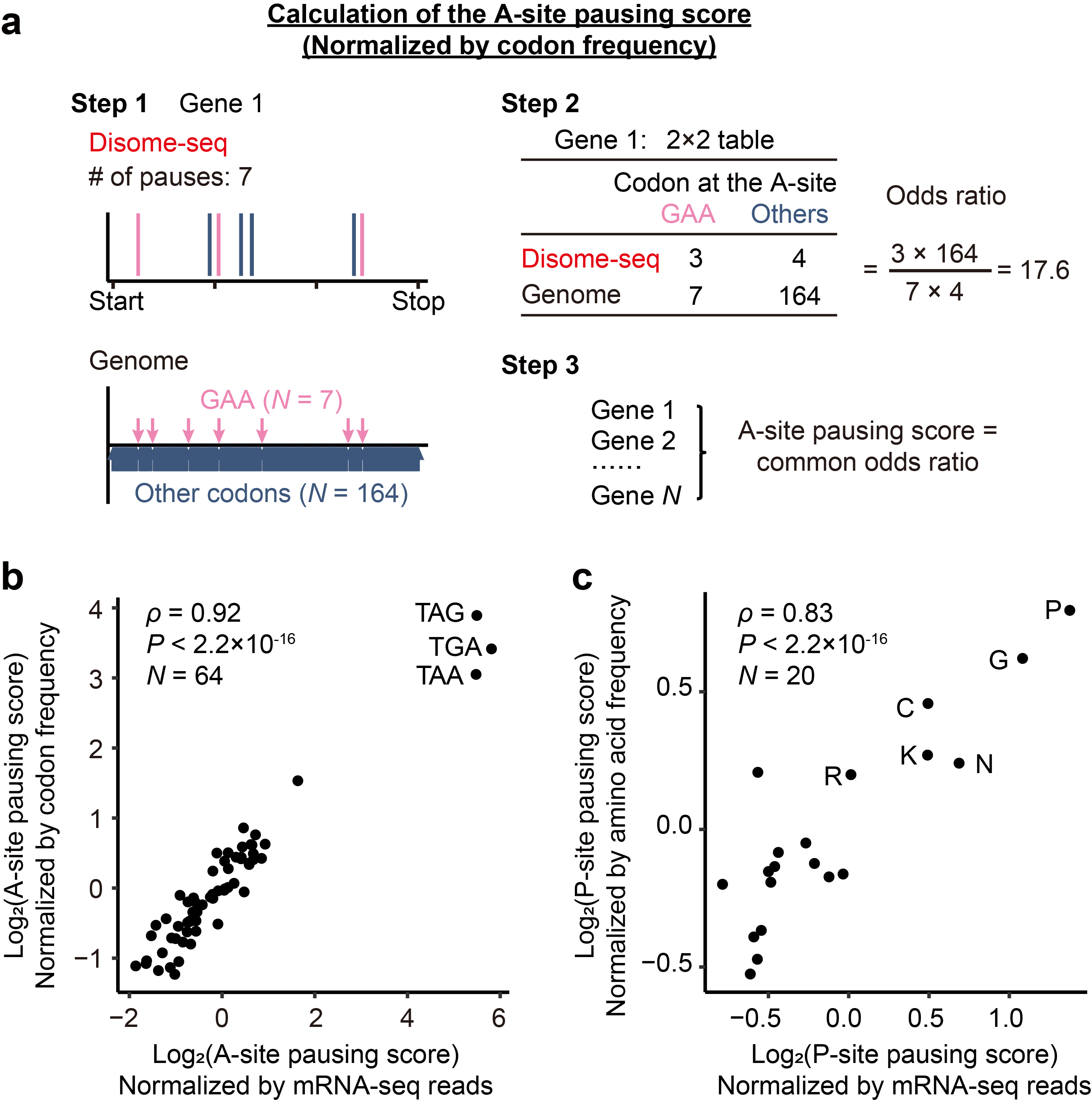


**Fig. S10. The pausing scores estimated using the codon/amino-acid frequency in the genome as the background.**

**a** Similar to **Fig. 3b**, the schematic shows the calculation of the A-site pausing score, but using codon frequencies in individual transcripts as the background. In alignment with the codon frequency information in the genomic sequence, only position information of disome footprints was used for this calculation (abundance information was omitted). Pink and blue arrows indicate the positions of GAA and the other codons, respectively.

**b** The scatter plot shows the A-site pausing scores for disome footprints using mRNA-seq reads or codon frequencies as the background.

**c** The scatter plot shows the P-site pausing scores for disome footprints using mRNA-seq reads or amino-acid frequencies as the background.


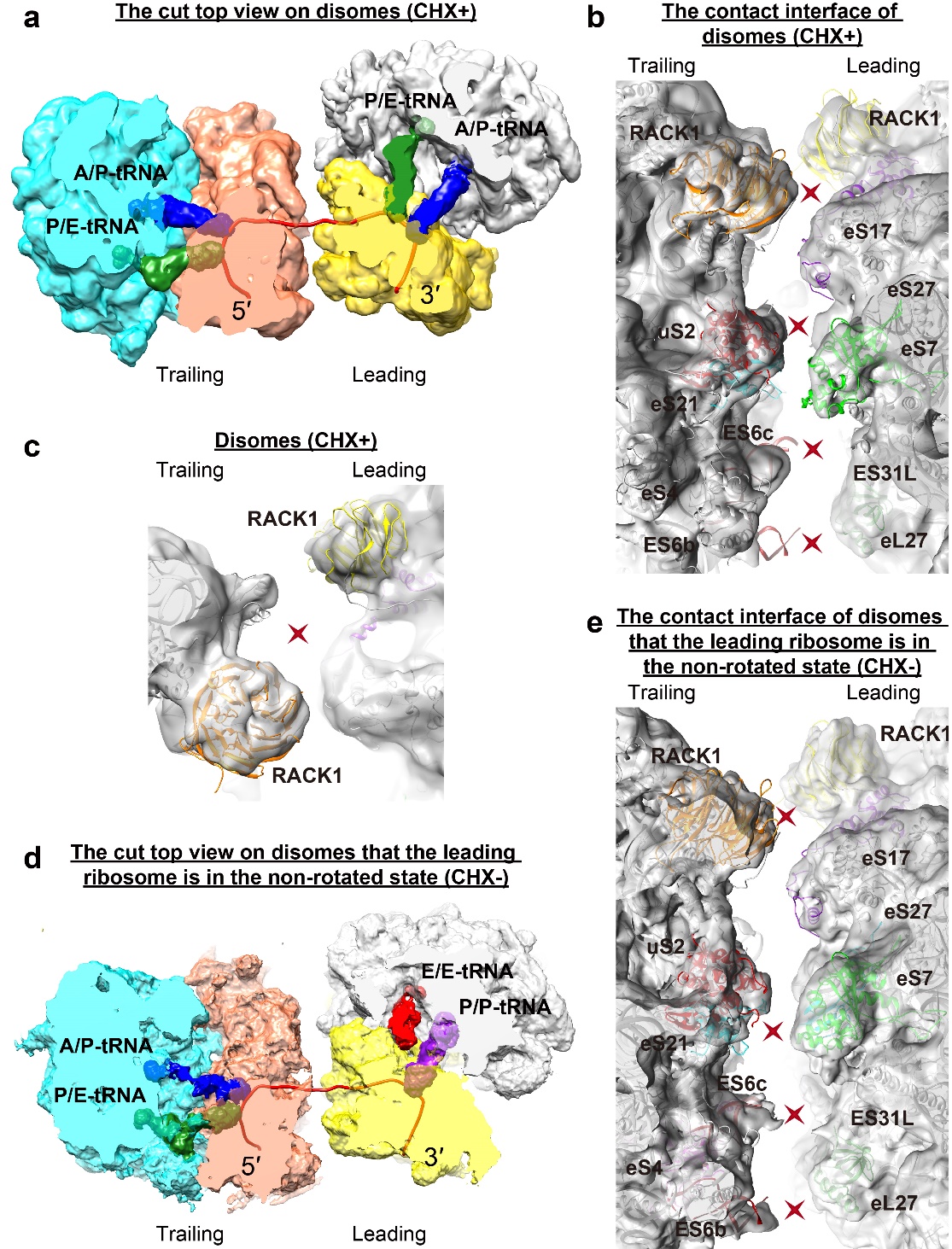


**Fig. S11. Additional cryo-EM structures of disomes.**

**a-c** Similar to **Fig. 6a, c, d**, but the disomes were collected with cycloheximide in the lysis buffer (CHX+). (**a**) A composite map of the cryo-EM structure of disomes. (**b**) The zoomed-in detail of the contact interface between the 40S subunits of the leading and trailing ribosomes. (**c**) The zoomed-in view of the 40S head-to-head contact site of disomes.

**d, e** Similar to **Fig. 6a, c**, but showing 13.7% of the total disome particles that the leading ribosome is in the non-rotated state harboring P/P (purple) and E/E tRNAs (red). The disomes were collected with cycloheximide omitted in the lysate (CHX−). (**d**) A composite map of the cryo-EM structure of disomes. (**e**) The zoomed-in detail of the contact interface between the 40S subunits of the leading and trailing ribosomes. Note that the non-rotated state of the leading ribosome does not necessarily mean that these disomes are the substrates for RQC. In our cryo-EM analyses, (27631/197367=) 14.0% monosomes obtained from yeast cells growing in the rich medium are in the non-rotated state, suggesting that the non-rotated leading ribosomes detected in disomes could merely be part of the regular work cycle of translation elongation. Furthermore, as indicated in (**e**), the contact interface between the 40S subunits of the leading and trailing ribosomes remains relatively flexible (compared to the di-ribosome), even if the leading ribosome is in the non-rotated state.


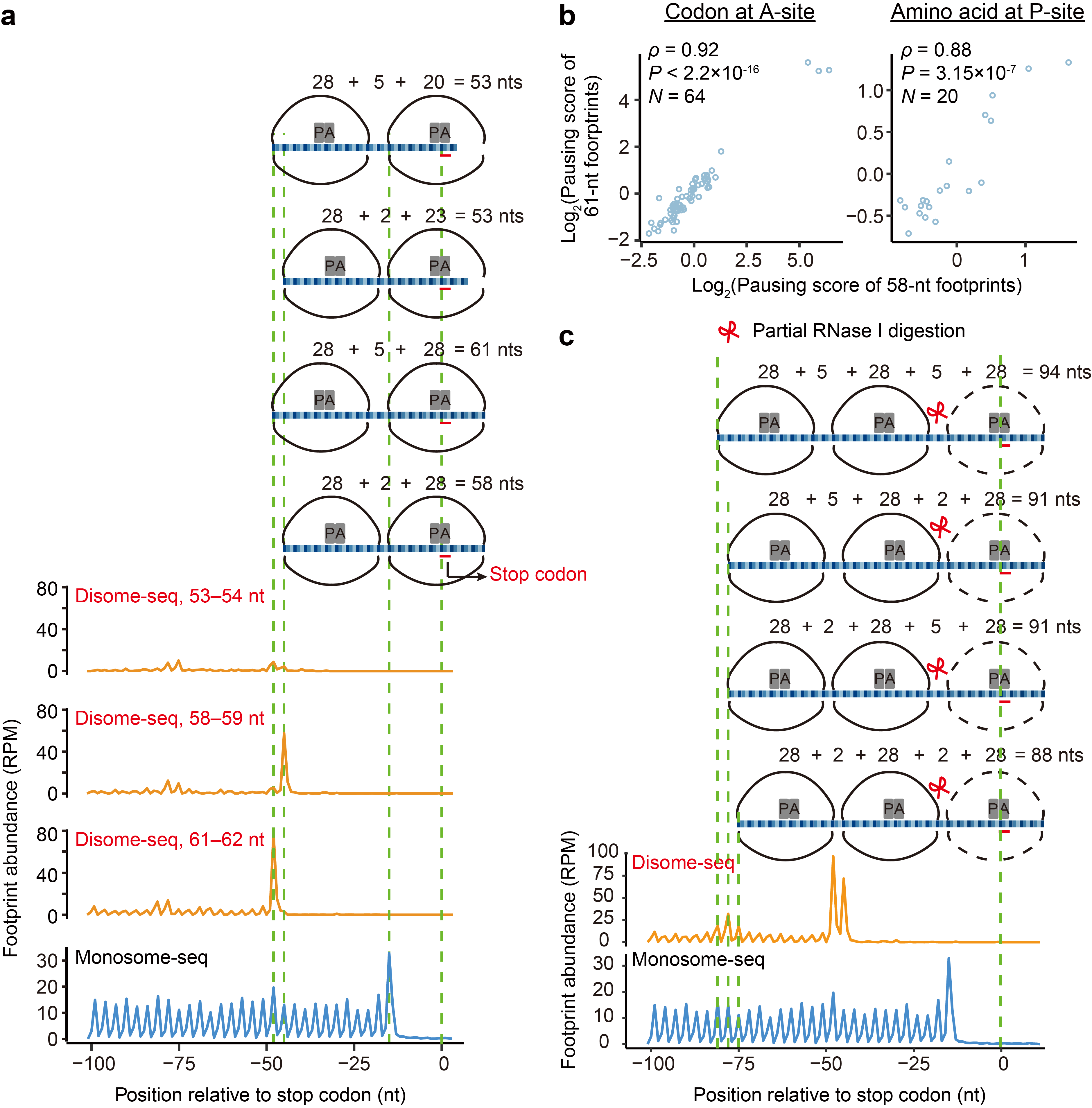


**Fig. S12. On the 61-nt disome footprints.**

**a** The aggregated abundance profile around the stop codon of the 5′-end of the monosome (blue) and disome footprints (orange). The footprint abundance at each nucleotide site was normalized by the total reads of the corresponding gene before aggregation. The presumed conformations of the 53-nt, the 58-nt, and the 61-nt disome footprints are shown. The 53-nt disome footprints are likely protected by a mix of 28+2+23 and 28+5+20 disomes, as indicated by the −45^th^ nt and −48^th^ nt peaks upstream of the stop codon. Since the 53-nt and the 58-nt disome footprints share the same 5′-end relative to the A-site of the leading ribosome in the 3-AT treated cells (**Fig. 1e** and **Additional file 1: Fig. S13b**), we focused on the 28+2+23 conformation in the rest of this study.

**b** The scatter plots show the A-site or the P-site pausing scores calculated from the 58–59 nt or the 61–62 nt disome footprints. The *P*-values were given by Spearman’s correlation.

**c** The conformations of trisomes, inferred from disome footprints around the stop codon. Disome footprints with the 5′-end ~75-nt upstream of the stop codon were generated likely through the partial RNase I digestion between the middle and the leading ribosome of a trisome.


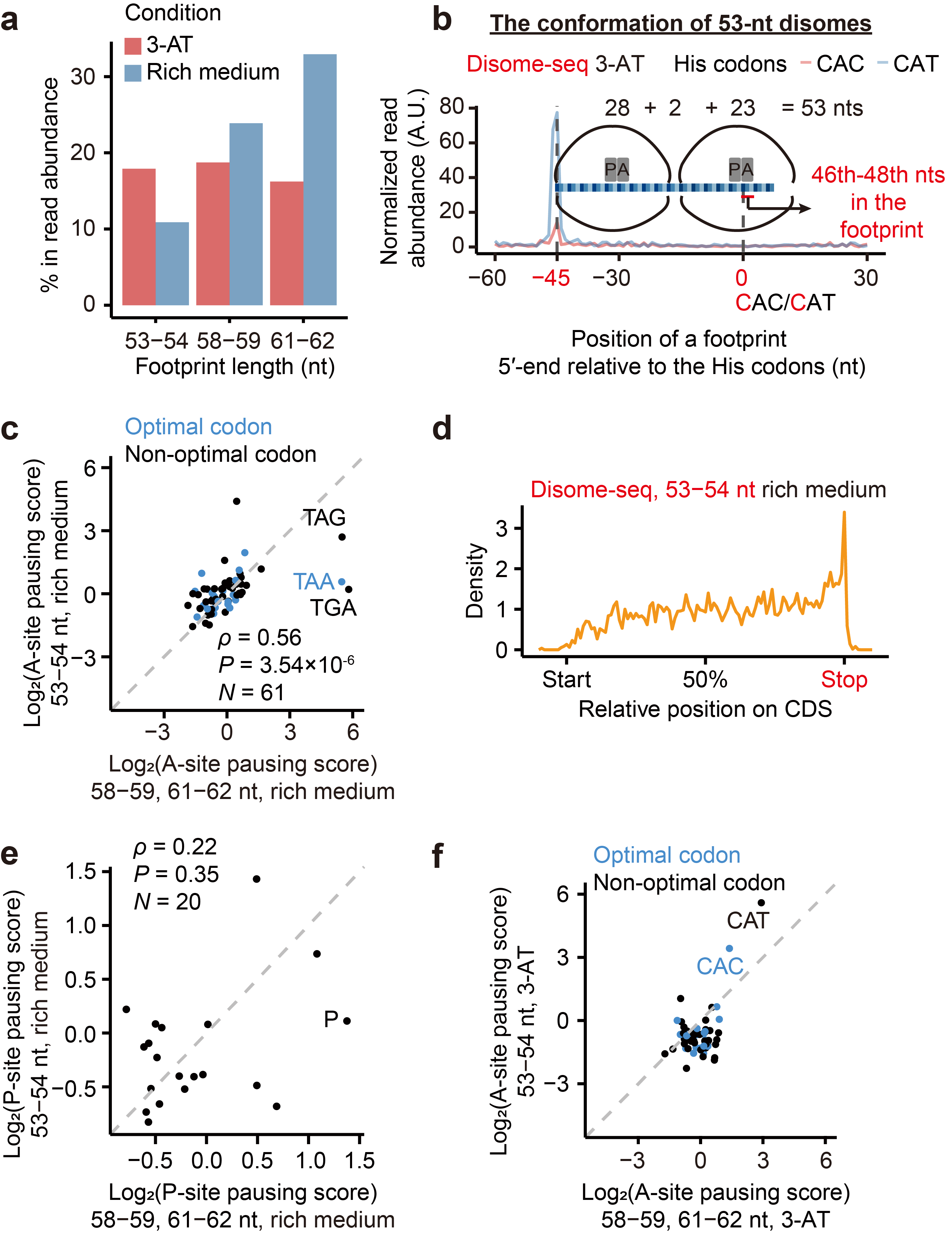


**Fig. S13. On the 53-nt disome footprints.**

**a** The percentage of disomes footprints with various lengths among all disomes footprints with the length ranging from 50 to 65 nts.

**b** Similar to **Fig. 1e**, the conformation of the 53-nt disome footprints was determined using disome footprints obtained from 3-AT treated yeast cells. The average footprint abundance of two replicates is shown.

**c** A scatter plot shows the A-site pausing scores estimated from the 58–59-nt/61–62-nt disome footprints and that from the 53 and 54-nt disome footprints, for cells growing in the rich medium. The dashed line shows *y* = *x*. The correlation for the 61 codons encoding amino acids was given by Spearman’s correlation.

**d** An aggregated profile of the 53 and 54-nt disome footprints density over 1876 genes, normalized against the CDS length, is shown.

**e** Similar to (**c**), showing the P-site pausing scores for the 20 amino acids. P, proline.

**f** Similar to (**c**), showing the results for 3-AT treated cells.


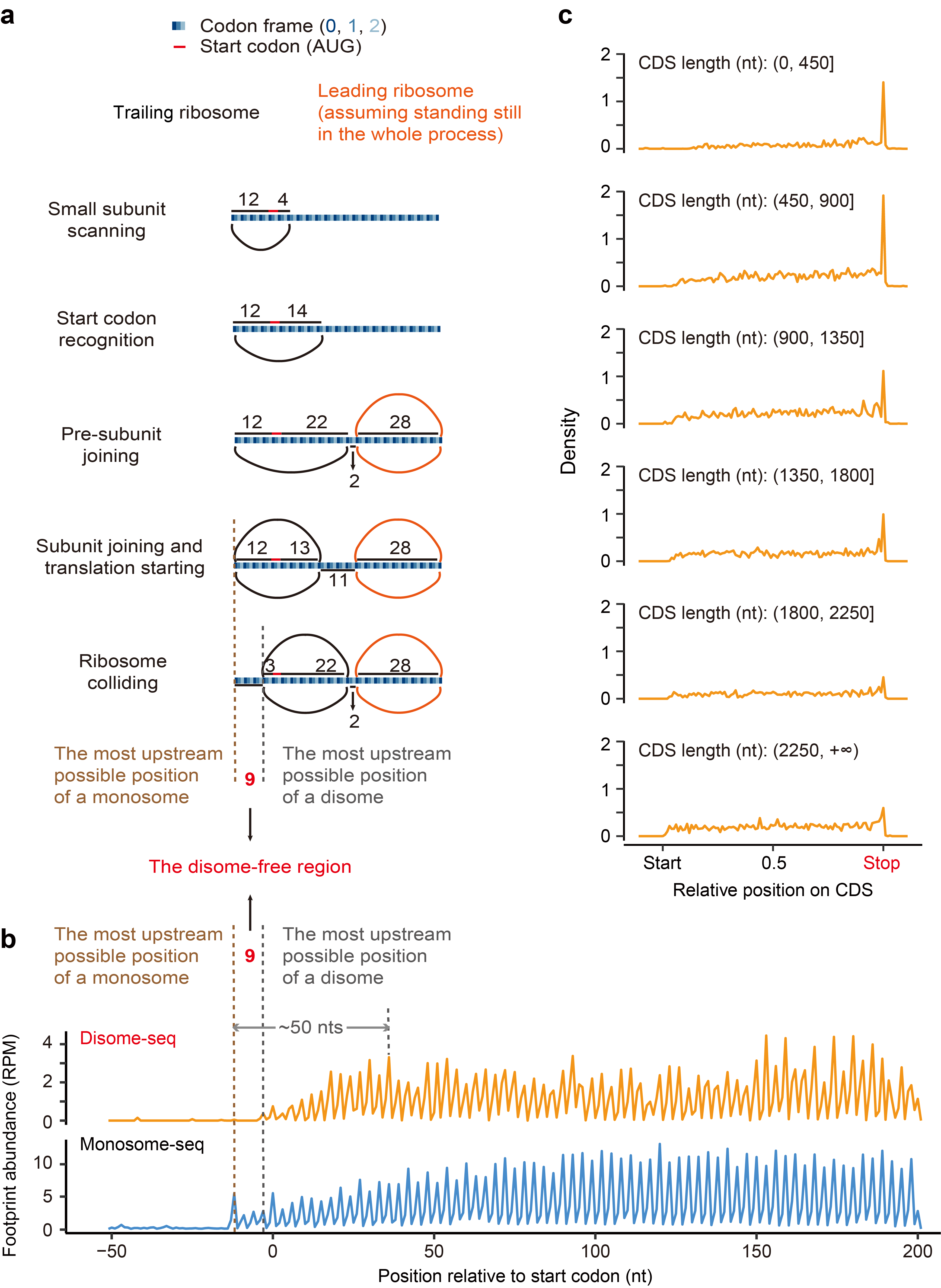


**Fig. S14. The “inverse ramp” of disome footprints on the CDS.**

**a** The “theoretical” most upstream position of a disome. A 9-nt disome-free region is predicted based on the reported conformational change of the 40S ribosomal small subunit during subunit joining (Archer *et al*., 2016).

**b** The 9-nt disome-free region is detected in the empirical data. The aggregated abundance profiles (aligned by the start codon) show the distribution of the 5′-end of monosome (blue) and disome footprints (orange). The footprint abundance at a site was normalized by the total footprints of the corresponding gene before aggregation.

**c** Aggregated profiles of disome footprint densities, normalized against the CDS length. Genes are sorted into six groups according to the CDS length.


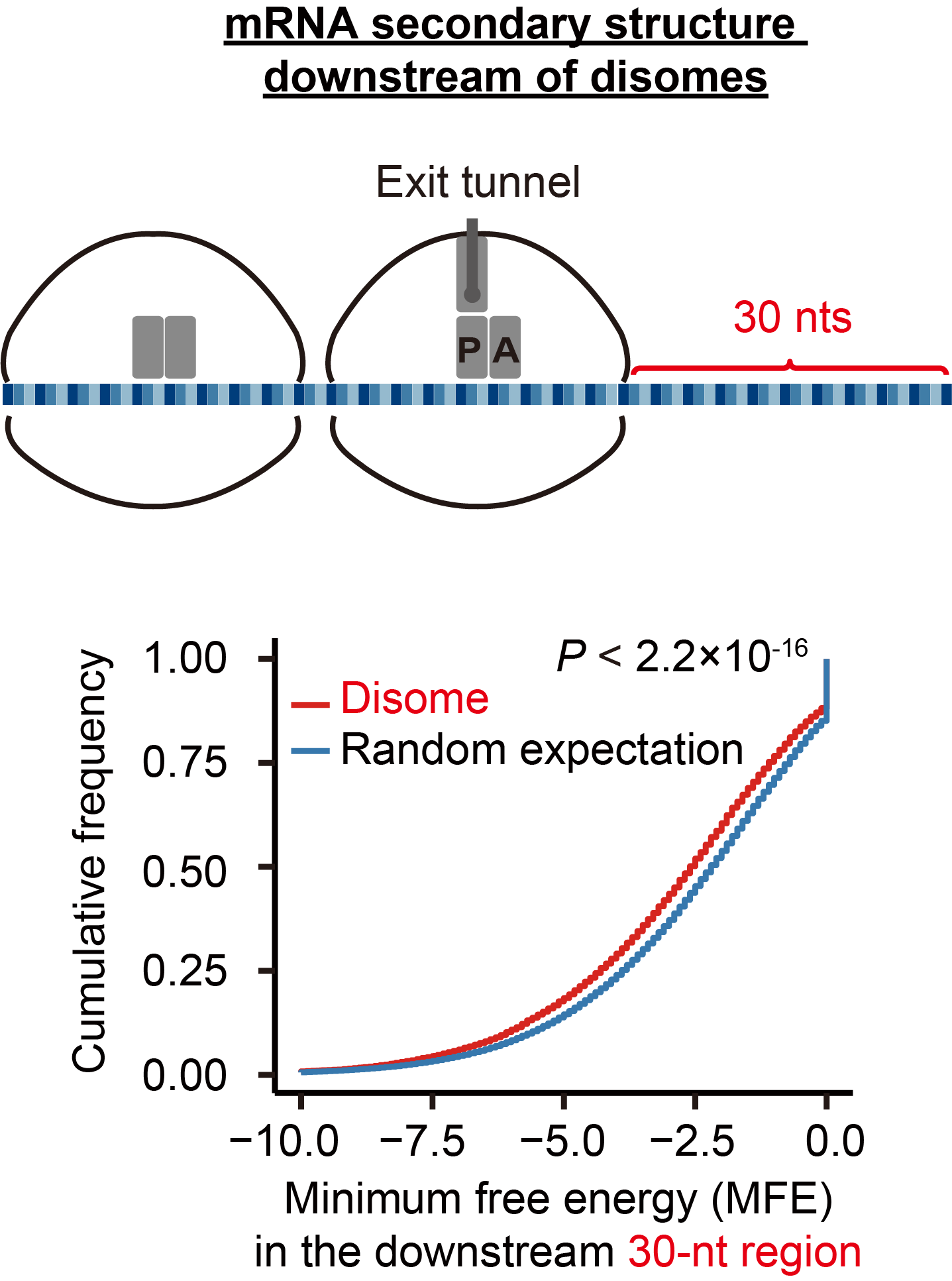


**Fig. S15. The mRNA secondary structure downstream of disome footprints.**

The red line shows the cumulative curve of the minimum free energy (MFE) of the 30-nt mRNA fragments downstream of disome footprints (red line). Random 30-nt mRNA fragments on the corresponding genes were used as the background (blue line). *P*-value was given by the Kolmogorov-Smirnov test.


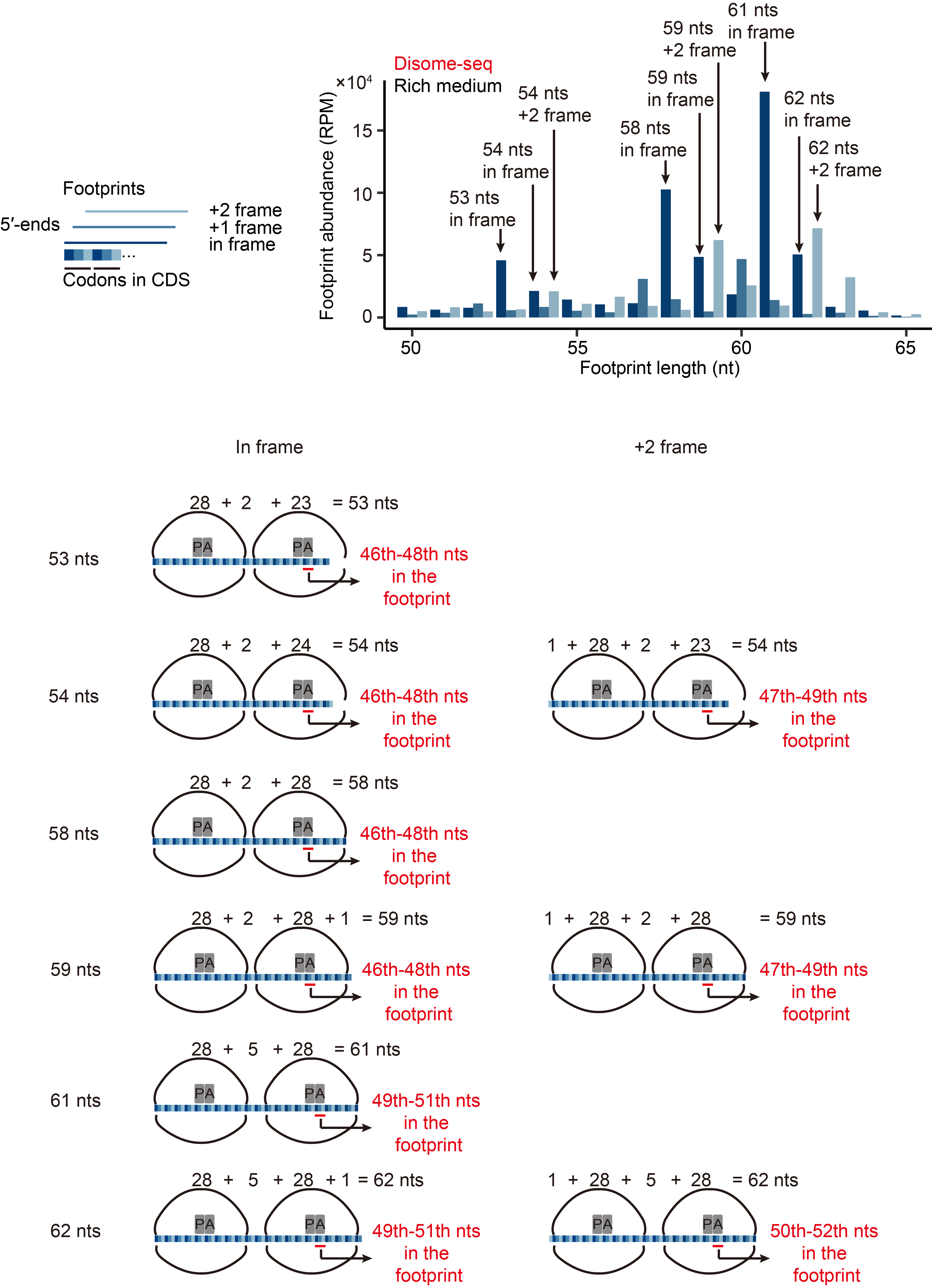


**Fig. S16. The presumed conformation** **and the putative A-site for disome footprints of various lengths**

The bar plot shows the length and reading frame (according to the 5′-end) distribution of the disome footprints obtained from yeast cells growing in the rich medium (combined from two biological replicates). The presumed conformation and the putative A-site are shown at the bottom for the disome footprints indicated in the bar plot.


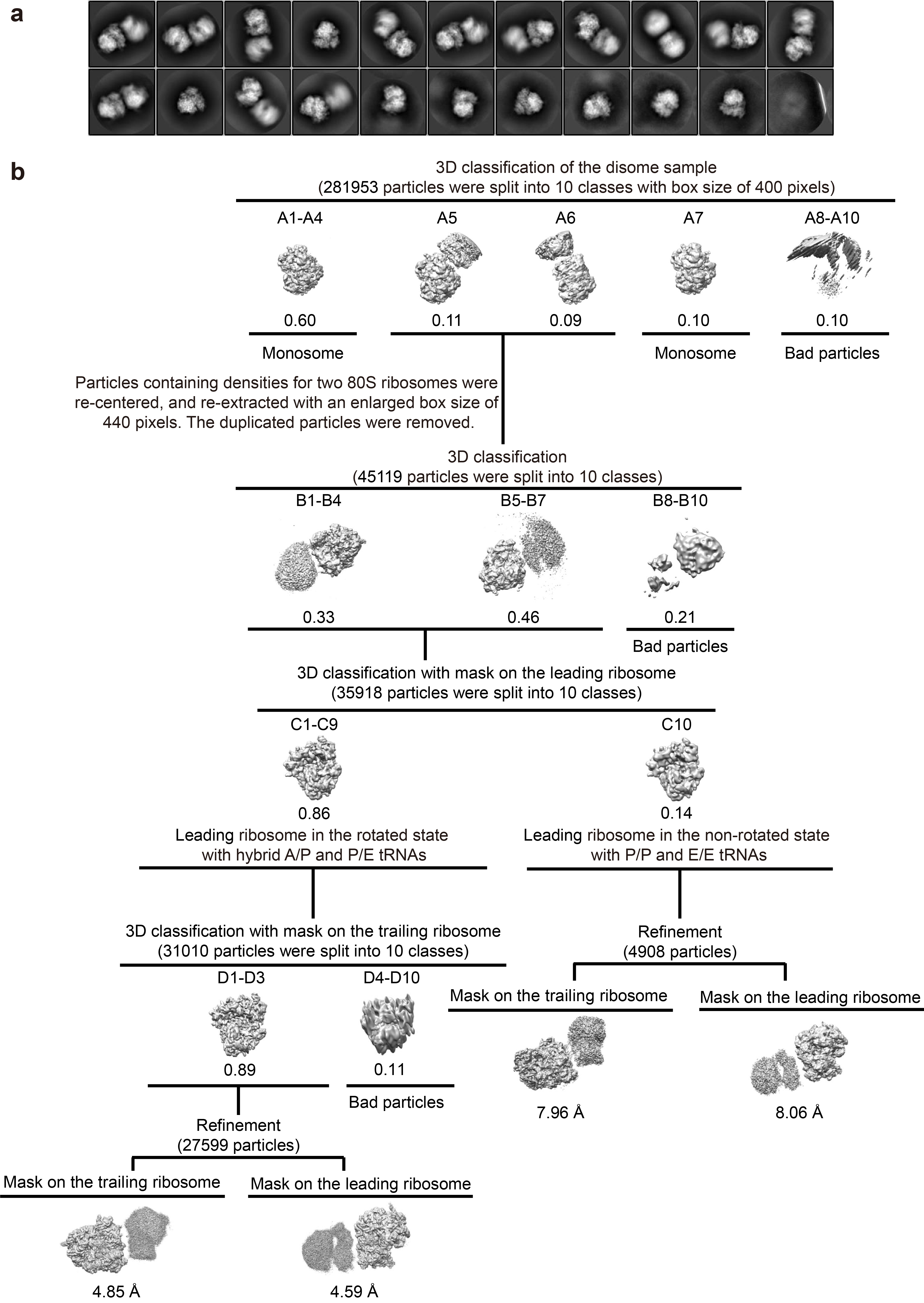


**Fig. S17. Cryo-EM data processing for disome particles that were collected with cycloheximide omitted in the lysis buffer.**

**a** Representative 2D class averages of disome particles.

**b** The flow chart for 3D classification and refinement.


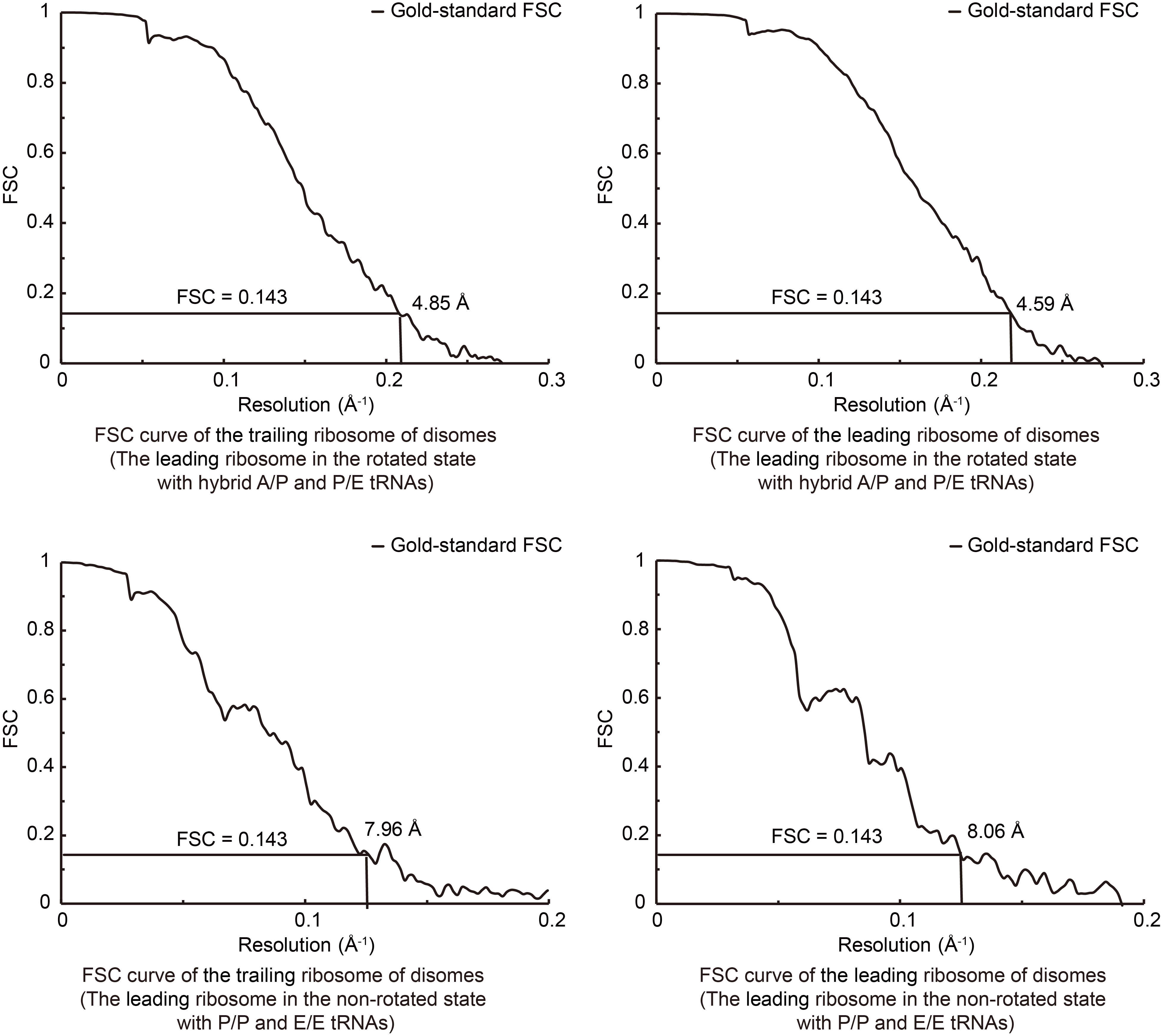


**Fig. S18. Fourier shell correlation (FSC) curves for the final 3D density maps after RELION-based post-processing.**

Disomes were collected with cycloheximide omitted in the lysis buffer.

**SUPPLEMENTARY TABLES**

**Table S1. Summary of the monosome-seq libraries.**

| Library | Total reads | Filtered reads^a^ | Uniquely mapped reads^b^ |
| --- | --- | --- | --- |
| Rich medium replicate 1 | 24693292 | 2420449 | 420358 |
| Rich medium replicate 2 | 20833790 | 1659124 | 336258 |
| 3-AT replicate 1 | 15470359 | 1897389 | 157989 |
| 3-AT replicate 2 | 14574600 | 1675158 | 147562 |
| Control in Fig. S8 | 26377014 | 5206211 | 858732 |

^a^ Reads after removing rRNA.

^b^ Reads uniquely mapped to the yeast genome.

**Table S2. Summary of the disome-seq libraries.**

| Library | Total reads | Filtered reads^a^ | Uniquely mapped reads^b^ |
| --- | --- | --- | --- |
| Rich medium replicate 1 | 272473666 | 13373574 | 2628459 |
| Rich medium replicate 2 | 263337526 | 4410686 | 681055 |
| 3-AT replicate 1 | 36139349 | 6144821 | 36084 |
| 3-AT replicate 2 | 39727602 | 8068750 | 44211 |

^a^ Reads after removing rRNA.

^b^ Reads uniquely mapped to the yeast genome.

**Table S3. Summary of the mRNA-seq libraries.**

| Library | Total reads | Uniquely mapped reads^a^ |
| --- | --- | --- |
| Rich medium replicate 1 | 5478547 | 1337661 |
| Rich medium replicate 2 | 6670236 | 1580266 |
| 3-AT replicate 1 | 6289058 | 1861188 |
| 3-AT replicate 2 | 4062390 | 1322674 |

^a^ Reads uniquely mapped to the yeast genome.

**Table S4. Disome-associated proteins (replicate 1: heavy isotope labeled disome proteins, disome/monosome intensity ratio > 1.5).**

| **ORF** | **Gene** | **Description*** |
| --- | --- | --- |
| **Chaperone** | | |

| YAL005C | *SSA1* | ATPase involved in protein folding and nuclear localization signal (NLS)-directed nuclear transport; member of heat shock protein 70 (HSP70) family; forms a chaperone complex with Ydj1p; localized to the nucleus, cytoplasm, and cell wall; 98% identical with Ssa2p, but subtle differences between the two proteins provide functional specificity with respect to propagation of yeast [URE3] prions and vacuolar-mediated degradations of gluconeogenesis enzymes |
| --- | --- | --- |
| YLL024C | *SSA2* | ATP binding protein involved in protein folding and vacuolar import of proteins; member of heat shock protein 70 (HSP70) family; associated with the chaperonin-containing T-complex; present in the cytoplasm, vacuolar membrane and cell wall; 98% identical with Ssa1p, but subtle differences between the two proteins provide functional specificity with respect to propagation of yeast [URE3] prions and vacuolar-mediated degradations of gluconeogenesis enzymes |
| YDL229W | *SSB1* | Cytoplasmic ATPase that is a ribosome-associated molecular chaperone, functions with J-protein partner Zuo1p; may be involved in folding of newly-made polypeptide chains; member of the HSP70 family; interacts with phosphatase subunit Reg1p |
| YNL209W | *SSB2* | Cytoplasmic ATPase that is a ribosome-associated molecular chaperone, functions with J-protein partner Zuo1p; may be involved in the folding of newly-synthesized polypeptide chains; member of the HSP70 family; homolog of SSB1 |
| YMR186W | *HSC82* | Cytoplasmic chaperone of the Hsp90 family, redundant in function and nearly identical with Hsp82p, and together they are essential; expressed constitutively at 10-fold higher basal levels than HSP82 and induced 2-3 fold by heat shock |
| YPL240C | *HSP82* | Hsp90 chaperone required for pheromone signaling and negative regulation of Hsf1p; docks with Tom70p for mitochondrial preprotein delivery; promotes telomerase DNA binding and nucleotide addition; interacts with Cns1p, Cpr6p, Cpr7p, Sti1p |
| YJL159W | *HSP150* | O-mannosylated heat shock protein that is secreted and covalently attached to the cell wall via beta-1,3-glucan and disulfide bridges; required for cell wall stability; induced by heat shock, oxidative stress, and nitrogen limitation |
| YIL016W | *SNL1* | Protein of unknown function proposed to be involved in nuclear pore complex biogenesis and maintenance as well as protein folding; has similarity to the mammalian BAG-1 protein |
| YJL008C | *CCT8* | Subunit of the cytosolic chaperonin Cct ring complex, related to Tcp1p, required for the assembly of actin and tubulins in vivo |
| YDR033W | *MRH1* | Protein that localizes primarily to the plasma membrane, also found at the nuclear envelope; the authentic, non-tagged protein is detected in mitochondria in a phosphorylated state; has similarity to Hsp30p and Yro2p |
| YNL064C | *YDJ1* | Type I HSP40 co-chaperone involved in regulation of the HSP90 and HSP70 functions; involved in protein translocation across membranes; member of the DnaJ family |

| **Helicase** | | |
| --- | --- | --- |
| YJL138C | *TIF2* | Translation initiation factor eIF4A, identical to Tif1p; DEA(D/H)-box RNA helicase that couples ATPase activity to RNA binding and unwinding; forms a dumbbell structure of two compact domains connected by a linker; interacts with eIF4G |
| YKR059W | *TIF1* | Translation initiation factor eIF4A, identical to Tif2p; DEA(D/H)-box RNA helicase that couples ATPase activity to RNA binding and unwinding; forms a dumbbell structure of two compact domains connected by a linker; interacts with eIF4G |
| **Metabolic** | | |
| YPL061W | *ALD6* | Cytosolic aldehyde dehydrogenase, activated by Mg2+ and utilizes NADP+ as the preferred coenzyme; required for conversion of acetaldehyde to acetate; constitutively expressed; locates to the mitochondrial outer surface upon oxidative stress |
| YGR192C | *TDH3* | Glyceraldehyde-3-phosphate dehydrogenase, isozyme 3, involved in glycolysis and gluconeogenesis; tetramer that catalyzes the reaction of glyceraldehyde-3-phosphate to 1,3 bis-phosphoglycerate; detected in the cytoplasm and cell wall |
| YJR009C | *TDH2* | Glyceraldehyde-3-phosphate dehydrogenase, isozyme 2, involved in glycolysis and gluconeogenesis; tetramer that catalyzes the reaction of glyceraldehyde-3-phosphate to 1,3 bis-phosphoglycerate; detected in the cytoplasm and cell wall |
| YOL086C | *ADH1* | Alcohol dehydrogenase, fermentative isozyme active as homo- or heterotetramers; required for the reduction of acetaldehyde to ethanol, the last step in the glycolytic pathway |
| YDR502C | *SAM2* | S-adenosylmethionine synthetase, catalyzes transfer of the adenosyl group of ATP to the sulfur atom of methionine; one of two differentially regulated isozymes (Sam1p and Sam2p) |
| YLR180W | *SAM1* | S-adenosylmethionine synthetase, catalyzes transfer of the adenosyl group of ATP to the sulfur atom of methionine; one of two differentially regulated isozymes (Sam1p and Sam2p) |
| YLR044C | *PDC1* | Major of three pyruvate decarboxylase isozymes, key enzyme in alcoholic fermentation, decarboxylates pyruvate to acetaldehyde; subject to glucose-, ethanol-, and autoregulation; involved in amino acid catabolism |
| YPL231W | *FAS2* | Alpha subunit of fatty acid synthetase, which catalyzes the synthesis of long-chain saturated fatty acids; contains the acyl-carrier protein domain and beta-ketoacyl reductase, beta-ketoacyl synthase and self-pantetheinylation activities |
| YJL130C | *URA2* | Bifunctional carbamoylphosphate synthetase (CPSase)-aspartate transcarbamylase (ATCase), catalyzes the first two enzymatic steps in the de novo biosynthesis of pyrimidines; both activities are subject to feedback inhibition by UTP |
| YBR196C | *PGI1* | Glycolytic enzyme phosphoglucose isomerase, catalyzes the interconversion of glucose-6-phosphate and fructose-6-phosphate; required for cell cycle progression and completion of the gluconeogenic events of sporulation |
| YKL060C | *FBA1* | Fructose 1,6-bisphosphate aldolase, required for glycolysis and gluconeogenesis; catalyzes conversion of fructose 1,6 bisphosphate to glyceraldehyde-3-P and dihydroxyacetone-P; locates to mitochondrial outer surface upon oxidative stress |
| YAL038W | *CDC19* | Pyruvate kinase, functions as a homotetramer in glycolysis to convert phosphoenolpyruvate to pyruvate, the input for aerobic (TCA cycle) or anaerobic (glucose fermentation) respiration |
| YKL182W | *FAS1* | Beta subunit of fatty acid synthetase, which catalyzes the synthesis of long-chain saturated fatty acids; contains acetyltransacylase, dehydratase, enoyl reductase, malonyl transacylase, and palmitoyl transacylase activities |
| YKL152C | *GPM1* | Tetrameric phosphoglycerate mutase, mediates the conversion of 3-phosphoglycerate to 2-phosphoglycerate during glycolysis and the reverse reaction during gluconeogenesis |
| YIL078W | *THS1* | Threonyl-tRNA synthetase, essential cytoplasmic protein |
| YGR254W | *ENO1* | Enolase I, a phosphopyruvate hydratase that catalyzes the conversion of 2-phosphoglycerate to phosphoenolpyruvate during glycolysis and the reverse reaction during gluconeogenesis; expression is repressed in response to glucose |
| YHR174W | *ENO2* | Enolase II, a phosphopyruvate hydratase that catalyzes the conversion of 2-phosphoglycerate to phosphoenolpyruvate during glycolysis and the reverse reaction during gluconeogenesis; expression is induced in response to glucose |
| YMR205C | *PFK2* | Beta subunit of heterooctameric phosphofructokinase involved in glycolysis, indispensable for anaerobic growth, activated by fructose-2,6-bisphosphate and AMP, mutation inhibits glucose induction of cell cycle-related genes |
| YHR019C | *DED81* | Cytosolic asparaginyl-tRNA synthetase, required for protein synthesis, catalyzes the specific attachment of asparagine to its cognate tRNA |
| YJL153C | *INO1* | Inositol-3-phosphate synthase, involved in synthesis of inositol phosphates and inositol-containing phospholipids; transcription is coregulated with other phospholipid biosynthetic genes by Ino2p and Ino4p, which bind the UASINO DNA element |
| YLR354C | *TAL1* | Transaldolase, enzyme in the non-oxidative pentose phosphate pathway; converts sedoheptulose 7-phosphate and glyceraldehyde 3-phosphate to erythrose 4-phosphate and fructose 6-phosphate |
| YDR050C | *TPI1* | Triose phosphate isomerase, abundant glycolytic enzyme; mRNA half-life is regulated by iron availability; transcription is controlled by activators Reb1p, Gcr1p, and Rap1p through binding sites in the 5′ non-coding region |
| YNR016C | *ACC1* | Acetyl-CoA carboxylase, biotin containing enzyme that catalyzes the carboxylation of acetyl-CoA to form malonyl-CoA; required for de novo biosynthesis of long-chain fatty acids |
| YCR012W | *PGK1* | 3-phosphoglycerate kinase, catalyzes transfer of high-energy phosphoryl groups from the acyl phosphate of 1,3-bisphosphoglycerate to ADP to produce ATP; key enzyme in glycolysis and gluconeogenesis |
| **Other** | | |
| YMR307W | *GAS1* | Beta-1,3-glucanosyltransferase, required for cell wall assembly and also has a role in transcriptional silencing; localizes to the cell surface via a glycosylphosphatidylinositol (GPI) anchor; also found at the nuclear periphery |
| YGR282C | *BGL2* | Endo-beta-1,3-glucanase, major protein of the cell wall, involved in cell wall maintenance |
| YFL039C | *ACT1* | Actin, structural protein involved in cell polarization, endocytosis, and other cytoskeletal functions |
| YDL055C | *PSA1* | GDP-mannose pyrophosphorylase (mannose-1-phosphate guanyltransferase), synthesizes GDP-mannose from GTP and mannose-1-phosphate in cell wall biosynthesis; required for normal cell wall structure |
| YKL164C | *PIR1* | O-glycosylated protein required for cell wall stability; attached to the cell wall via beta-1,3-glucan; mediates mitochondrial translocation of Apn1p; expression regulated by the cell integrity pathway and by Swi5p during the cell cycle |
| YBR009C | *HHF1* | Histone H4, core histone protein required for chromatin assembly and chromosome function; one of two identical histone proteins (see also HHF2); contributes to telomeric silencing; N-terminal domain involved in maintaining genomic integrity |
| YNL030W | *HHF2* | Histone H4, core histone protein required for chromatin assembly and chromosome function; one of two identical histone proteins (see also HHF1); contributes to telomeric silencing; N-terminal domain involved in maintaining genomic integrity |
| YBL002W | *HTB2* | Histone H2B, core histone protein required for chromatin assembly and chromosome function; nearly identical to HTB1; Rad6p-Bre1p-Lge1p mediated ubiquitination regulates transcriptional activation, meiotic DSB formation and H3 methylation |
| YDR224C | *HTB1* | Histone H2B, core histone protein required for chromatin assembly and chromosome function; nearly identical to HTB2; Rad6p-Bre1p-Lge1p mediated ubiquitination regulates transcriptional activation, meiotic DSB formation and H3 methylation |
| YFL037W | *TUB2* | Beta-tubulin; associates with alpha-tubulin (Tub1p and Tub3p) to form tubulin dimer, which polymerizes to form microtubules |
| YNL284C | *MRPL10* | Mitochondrial ribosomal protein of the large subunit; appears as two protein spots (YmL10 and YmL18) on two-dimensional SDS gels |
| YDL202W | *MRPL11* | Mitochondrial ribosomal protein of the large subunit |
| YPL093W | *NOG1* | Putative GTPase that associates with free 60S ribosomal subunits in the nucleolus and is required for 60S ribosomal subunit biogenesis; constituent of 66S pre-ribosomal particles; member of the ODN family of nucleolar G-proteins |
| YPR016C | *TIF6* | Constituent of 66S pre-ribosomal particles, has similarity to human translation initiation factor 6 (eIF6); may be involved in the biogenesis and or stability of 60S ribosomal subunits |
| YER006W | *NUG1* | GTPase that associates with nuclear 60S pre-ribosomes, required for export of 60S ribosomal subunits from the nucleus |
| YHR088W | *RPF1* | Nucleolar protein involved in the assembly and export of the large ribosomal subunit; constituent of 66S pre-ribosomal particles; contains a sigma(70)-like motif, which is thought to bind RNA |
| YMR012W | *CLU1* | eIF3 component of unknown function; deletion causes defects in mitochondrial organization but not in growth or translation initiation, can rescue cytokinesis and mitochondrial organization defects of the Dictyostelium cluA- mutant |
| YDR385W | *EFT2* | Elongation factor 2 (EF-2), also encoded by EFT1; catalyzes ribosomal translocation during protein synthesis; contains diphthamide, the unique posttranslationally modified histidine residue specifically ADP-ribosylated by diphtheria toxin |
| YOR133W | *EFT1* | Elongation factor 2 (EF-2), also encoded by EFT2; catalyzes ribosomal translocation during protein synthesis; contains diphthamide, the unique posttranslationally modified histidine residue specifically ADP-ribosylated by diphtheria toxin |
| YLR249W | *YEF3* | Gamma subunit of translational elongation factor eEF1B, stimulates the binding of aminoacyl-tRNA (AA-tRNA) to ribosomes by releasing eEF1A (Tef1p/Tef2p) from the ribosomal complex; contains two ABC cassettes; binds and hydrolyzes ATP |
| YBR118W | *TEF2* | Translational elongation factor EF-1 alpha; also encoded by TEF1; functions in the binding reaction of aminoacyl-tRNA (AA-tRNA) to ribosomes; may also have a role in tRNA re-export from the nucleus |
| YPR080W | *TEF1* | Translational elongation factor EF-1 alpha; also encoded by TEF2; functions in the binding reaction of aminoacyl-tRNA (AA-tRNA) to ribosomes; may also have a role in tRNA re-export from the nucleus |
| YGL008C | *PMA1* | Plasma membrane H+-ATPase, pumps protons out of the cell; major regulator of cytoplasmic pH and plasma membrane potential; P2-type ATPase; Hsp30p plays a role in Pma1p regulation; interactions with Std1p appear to propagate [GAR+] |
| YPL036W | *PMA2* | Plasma membrane H+-ATPase, isoform of Pma1p, involved in pumping protons out of the cell; regulator of cytoplasmic pH and plasma membrane potential |
| YBR127C | *VMA2* | Subunit B of the eight-subunit V1 peripheral membrane domain of the vacuolar H+-ATPase (V-ATPase), an electrogenic proton pump found throughout the endomembrane system; contains nucleotide binding sites; also detected in the cytoplasm |
| YDL185W | *VMA1* | Subunit A of the eight-subunit V1 peripheral membrane domain of the vacuolar H+-ATPase; protein precursor undergoes self-catalyzed splicing to yield the extein Tfp1p and the intein Vde (PI-SceI), which is a site-specific endonuclease |
| YDR233C | *RTN1* | ER membrane protein that interacts with Sey1p to maintain ER morphology; interacts with exocyst subunit Sec6p, with Yip3p, and with Sbh1p; null mutant has an altered ER morphology; member of the RTNLA (reticulon-like A) subfamily |

* Descriptions were retrieved from the *Saccharomyces* Genome Database (<https://www.yeastgenome.org/>)

**Table S5. Disome-associated proteins (replicate 2: light isotope labeled disome proteins, disome/monosome intensity ratio > 1.5).**

| **ORF** | **Gene** | **Description*** |  |  |
| --- | --- | --- | --- | --- |
| **Chaperone** | | |  |  |
| YAL005C | *SSA1* | ATPase involved in protein folding and nuclear localization signal (NLS)-directed nuclear transport; member of heat shock protein 70 (HSP70) family; forms a chaperone complex with Ydj1p; localized to the nucleus, cytoplasm, and cell wall; 98% identical with Ssa2p, but subtle differences between the two proteins provide functional specificity with respect to propagation of yeast [URE3] prions and vacuolar-mediated degradations of gluconeogenesis enzymes |  |  |
| YLL024C | *SSA2* | ATP binding protein involved in protein folding and vacuolar import of proteins; member of heat shock protein 70 (HSP70) family; associated with the chaperonin-containing T-complex; present in the cytoplasm, vacuolar membrane and cell wall; 98% identical with Ssa1p, but subtle differences between the two proteins provide functional specificity with respect to propagation of yeast [URE3] prions and vacuolar-mediated degradations of gluconeogenesis enzymes |  |  |
| YDL229W | *SSB1* | Cytoplasmic ATPase that is a ribosome-associated molecular chaperone, functions with J-protein partner Zuo1p; may be involved in folding of newly-made polypeptide chains; member of the HSP70 family; interacts with phosphatase subunit Reg1p |  |  |
| YNL209W | *SSB2* | Cytoplasmic ATPase that is a ribosome-associated molecular chaperone, functions with J-protein partner Zuo1p; may be involved in the folding of newly-synthesized polypeptide chains; member of the HSP70 family; homolog of SSB1 |  |  |
| YMR186W | *HSC82* | Cytoplasmic chaperone of the Hsp90 family, redundant in function and nearly identical with Hsp82p, and together they are essential; expressed constitutively at 10-fold higher basal levels than HSP82 and induced 2-3 fold by heat shock |  |  |
| YPL240C | *HSP82* | Hsp90 chaperone required for pheromone signaling and negative regulation of Hsf1p; docks with Tom70p for mitochondrial preprotein delivery; promotes telomerase DNA binding and nucleotide addition; interacts with Cns1p, Cpr6p, Cpr7p, Sti1p |  |  |
| YJL159W | *HSP150* | O-mannosylated heat shock protein that is secreted and covalently attached to the cell wall via beta-1,3-glucan and disulfide bridges; required for cell wall stability; induced by heat shock, oxidative stress, and nitrogen limitation |  |  |
| **Helicase** | | | |  |
| YJL138C | *TIF2* | Translation initiation factor eIF4A, identical to Tif1p; DEA(D/H)-box RNA helicase that couples ATPase activity to RNA binding and unwinding; forms a dumbbell structure of two compact domains connected by a linker; interacts with eIF4G |  |  |
| YKR059W | *TIF1* | Translation initiation factor eIF4A, identical to Tif2p; DEA(D/H)-box RNA helicase that couples ATPase activity to RNA binding and unwinding; forms a dumbbell structure of two compact domains connected by a linker; interacts with eIF4G |  |  |
| YLR419W | *YLR419W* | Putative helicase with limited sequence similarity to human Rb protein; the authentic, non-tagged protein is detected in highly purified mitochondria in high-throughput studies; YLR419W is not an essential gene |  |  |
| **Metabolic** |  |  |  | |
| YOL086C | *ADH1* | Alcohol dehydrogenase, fermentative isozyme active as homo- or heterotetramers; required for the reduction of acetaldehyde to ethanol, the last step in the glycolytic pathway |  |  |
| YJL130C | *URA2* | Bifunctional carbamoylphosphate synthetase (CPSase)-aspartate transcarbamylase (ATCase), catalyzes the first two enzymatic steps in the de novo biosynthesis of pyrimidines; both activities are subject to feedback inhibition by UTP |  |  |
| YJR009C | *TDH2* | Glyceraldehyde-3-phosphate dehydrogenase, isozyme 2, involved in glycolysis and gluconeogenesis; tetramer that catalyzes the reaction of glyceraldehyde-3-phosphate to 1,3 bis-phosphoglycerate; detected in the cytoplasm and cell wall |  |  |
| YDR502C | *SAM2* | S-adenosylmethionine synthetase, catalyzes transfer of the adenosyl group of ATP to the sulfur atom of methionine; one of two differentially regulated isozymes (Sam1p and Sam2p) |  |  |
| YLR180W | *SAM1* | S-adenosylmethionine synthetase, catalyzes transfer of the adenosyl group of ATP to the sulfur atom of methionine; one of two differentially regulated isozymes (Sam1p and Sam2p) |  |  |
| YCL064C | *CHA1* | Catabolic L-serine (L-threonine) deaminase, catalyzes the degradation of both L-serine and L-threonine; required to use serine or threonine as the sole nitrogen source, transcriptionally induced by serine and threonine |  |  |
| YKL060C | *FBA1* | Fructose 1,6-bisphosphate aldolase, required for glycolysis and gluconeogenesis; catalyzes conversion of fructose 1,6 bisphosphate to glyceraldehyde-3-P and dihydroxyacetone-P; locates to mitochondrial outer surface upon oxidative stress |  |  |
| YLR044C | *PDC1* | Major of three pyruvate decarboxylase isozymes, key enzyme in alcoholic fermentation, decarboxylates pyruvate to acetaldehyde; subject to glucose-, ethanol-, and autoregulation; involved in amino acid catabolism |  |  |
| YKL182W | *FAS1* | Beta subunit of fatty acid synthetase, which catalyzes the synthesis of long-chain saturated fatty acids; contains acetyltransacylase, dehydratase, enoyl reductase, malonyl transacylase, and palmitoyl transacylase activities |  |  |
| YBR196C | *PGI1* | Glycolytic enzyme phosphoglucose isomerase, catalyzes the interconversion of glucose-6-phosphate and fructose-6-phosphate; required for cell cycle progression and completion of the gluconeogenic events of sporulation |  |  |
| YJR073C | *OPI3* | Phospholipid methyltransferase (methylene-fatty-acyl-phospholipid synthase), catalyzes the last two steps in phosphatidylcholine biosynthesis |  |  |
| YIL078W | *THS1* | Threonyl-tRNA synthetase, essential cytoplasmic protein |  | |
| YPL231W | *FAS2* | Alpha subunit of fatty acid synthetase, which catalyzes the synthesis of long-chain saturated fatty acids; contains the acyl-carrier protein domain and beta-ketoacyl reductase, beta-ketoacyl synthase and self-pantetheinylation activities |  |  |
| YER091C | *MET6* | Cobalamin-independent methionine synthase, involved in methionine biosynthesis and regeneration; requires a minimum of two glutamates on the methyltetrahydrofolate substrate, similar to bacterial metE homologs |  |  |
| YAL038W | *CDC19* | Pyruvate kinase, functions as a homotetramer in glycolysis to convert phosphoenolpyruvate to pyruvate, the input for aerobic (TCA cycle) or anaerobic (glucose fermentation) respiration |  |  |
| YHR174W | *ENO2* | Enolase II, a phosphopyruvate hydratase that catalyzes the conversion of 2-phosphoglycerate to phosphoenolpyruvate during glycolysis and the reverse reaction during gluconeogenesis; expression is induced in response to glucose |  |  |
| YLR355C | *ILV5* | Bifunctional acetohydroxyacid reductoisomerase and mtDNA binding protein; involved in branched-chain amino acid biosynthesis and maintenance of wild-type mitochondrial DNA; found in mitochondrial nucleoids |  |  |
| YMR205C | *PFK2* | Beta subunit of heterooctameric phosphofructokinase involved in glycolysis, indispensable for anaerobic growth, activated by fructose-2,6-bisphosphate and AMP, mutation inhibits glucose induction of cell cycle-related genes |  |  |
| YKL152C | *GPM1* | Tetrameric phosphoglycerate mutase, mediates the conversion of 3-phosphoglycerate to 2-phosphoglycerate during glycolysis and the reverse reaction during gluconeogenesis |  |  |
| YPR184W | *GDB1* | Glycogen debranching enzyme containing glucanotranferase and alpha-1,6-amyloglucosidase activities, required for glycogen degradation; phosphorylated in mitochondria; activity is inhibited by Igd1p |  |  |
| YCR012W | *PGK1* | 3-phosphoglycerate kinase, catalyzes transfer of high-energy phosphoryl groups from the acyl phosphate of 1,3-bisphosphoglycerate to ADP to produce ATP; key enzyme in glycolysis and gluconeogenesis |  |  |
| **Other** | | | |  |
| YDL055C | *PSA1* | GDP-mannose pyrophosphorylase (mannose-1-phosphate guanyltransferase), synthesizes GDP-mannose from GTP and mannose-1-phosphate in cell wall biosynthesis; required for normal cell wall structure |  |  |
| YBR078W | *ECM33* | GPI-anchored protein of unknown function, has a possible role in apical bud growth; GPI-anchoring on the plasma membrane crucial to function; phosphorylated in mitochondria; similar to Sps2p and Pst1p |  |  |
| YGR282C | *BGL2* | Endo-beta-1,3-glucanase, major protein of the cell wall, involved in cell wall maintenance |  |  |
| YGR279C | *SCW4* | Cell wall protein with similarity to glucanases; scw4 scw10 double mutants exhibit defects in mating |  |  |
| YKL164C | *PIR1* | O-glycosylated protein required for cell wall stability; attached to the cell wall via beta-1,3-glucan; mediates mitochondrial translocation of Apn1p; expression regulated by the cell integrity pathway and by Swi5p during the cell cycle |  |  |
| YBR009C | *HHF1* | Histone H4, core histone protein required for chromatin assembly and chromosome function; one of two identical histone proteins (see also HHF2); contributes to telomeric silencing; N-terminal domain involved in maintaining genomic integrity |  |  |
| YNL030W | *HHF2* | Histone H4, core histone protein required for chromatin assembly and chromosome function; one of two identical histone proteins (see also HHF1); contributes to telomeric silencing; N-terminal domain involved in maintaining genomic integrity |  |  |
| YFL037W | *TUB2* | Beta-tubulin; associates with alpha-tubulin (Tub1p and Tub3p) to form tubulin dimer, which polymerizes to form microtubules |  |  |
| YFL039C | *ACT1* | Actin, structural protein involved in cell polarization, endocytosis, and other cytoskeletal functions |  |  |
| YJR113C | *RSM7* | Mitochondrial ribosomal protein of the small subunit, has similarity to E. coli S7 ribosomal protein |  |  |
| YPL093W | *NOG1* | Putative GTPase that associates with free 60S ribosomal subunits in the nucleolus and is required for 60S ribosomal subunit biogenesis; constituent of 66S pre-ribosomal particles; member of the ODN family of nucleolar G-proteins |  |  |
| YER006W | *NUG1* | GTPase that associates with nuclear 60S pre-ribosomes, required for export of 60S ribosomal subunits from the nucleus |  |  |
| YPR016C | *TIF6* | Constituent of 66S pre-ribosomal particles, has similarity to human translation initiation factor 6 (eIF6); may be involved in the biogenesis and or stability of 60S ribosomal subunits |  |  |
| YHR088W | *RPF1* | Nucleolar protein involved in the assembly and export of the large ribosomal subunit; constituent of 66S pre-ribosomal particles; contains a sigma(70)-like motif, which is thought to bind RNA |  |  |
| YAL025C | *MAK16* | Essential nuclear protein, constituent of 66S pre-ribosomal particles; required for maturation of 25S and 5.8S rRNAs; required for maintenance of M1 satellite double-stranded RNA of the L-A virus |  |  |
| YGR103W | *NOP7* | Component of several different pre-ribosomal particles; forms a complex with Ytm1p and Erb1p that is required for maturation of the large ribosomal subunit; required for exit from G<sub0</sub and the initiation of cell proliferation |  |  |
| YHR072W-A | *NOP10* | Constituent of small nucleolar ribonucleoprotein particles containing H/ACA-type snoRNAs, which are required for pseudouridylation and processing of pre-18S rRNA |  |  |
| YOL077C | *BRX1* | Nucleolar protein, constituent of 66S pre-ribosomal particles; depletion leads to defects in rRNA processing and a block in the assembly of large ribosomal subunits; possesses a sigma(70)-like RNA-binding motif |  |  |
| YLR196W | *PWP1* | Protein with WD-40 repeats involved in rRNA processing; associates with trans-acting ribosome biogenesis factors; similar to beta-transducin superfamily |  |  |
| YLR175W | *CBF5* | Pseudouridine synthase catalytic subunit of box H/ACA small nucleolar ribonucleoprotein particles (snoRNPs), acts on both large and small rRNAs and on snRNA U2; mutations in human ortholog dyskerin cause the disorder dyskeratosis congenita |  |  |
| YPL061W | *ALD6* | Cytosolic aldehyde dehydrogenase, activated by Mg2+ and utilizes NADP+ as the preferred coenzyme; required for conversion of acetaldehyde to acetate; constitutively expressed; locates to the mitochondrial outer surface upon oxidative stress |  |  |
| YLR249W | *YEF3* | Gamma subunit of translational elongation factor eEF1B, stimulates the binding of aminoacyl-tRNA (AA-tRNA) to ribosomes by releasing eEF1A (Tef1p/Tef2p) from the ribosomal complex; contains two ABC cassettes; binds and hydrolyzes ATP |  |  |
| YKL081W | *TEF4* | Gamma subunit of translational elongation factor eEF1B, stimulates the binding of aminoacyl-tRNA (AA-tRNA) to ribosomes by releasing eEF1A (Tef1p/Tef2p) from the ribosomal complex |  |  |
| YDR385W | *EFT2* | Elongation factor 2 (EF-2), also encoded by EFT1; catalyzes ribosomal translocation during protein synthesis; contains diphthamide, the unique posttranslationally modified histidine residue specifically ADP-ribosylated by diphtheria toxin |  |  |
| YOR133W | *EFT1* | Elongation factor 2 (EF-2), also encoded by EFT2; catalyzes ribosomal translocation during protein synthesis; contains diphthamide, the unique posttranslationally modified histidine residue specifically ADP-ribosylated by diphtheria toxin |  |  |
| YMR012W | *CLU1* | eIF3 component of unknown function; deletion causes defects in mitochondrial organization but not in growth or translation initiation, can rescue cytokinesis and mitochondrial organization defects of the Dictyostelium cluA- mutant |  |  |
| YBR118W | *TEF2* | Translational elongation factor EF-1 alpha; also encoded by TEF1; functions in the binding reaction of aminoacyl-tRNA (AA-tRNA) to ribosomes; may also have a role in tRNA re-export from the nucleus |  |  |
| YPR080W | *TEF1* | Translational elongation factor EF-1 alpha; also encoded by TEF2; functions in the binding reaction of aminoacyl-tRNA (AA-tRNA) to ribosomes; may also have a role in tRNA re-export from the nucleus |  |  |
| YMR307W | *GAS1* | Beta-1,3-glucanosyltransferase, required for cell wall assembly and also has a role in transcriptional silencing; localizes to the cell surface via a glycosylphosphatidylinositol (GPI) anchor; also found at the nuclear periphery |  |  |
| YGL008C | *PMA1* | Plasma membrane H+-ATPase, pumps protons out of the cell; major regulator of cytoplasmic pH and plasma membrane potential; P2-type ATPase; Hsp30p plays a role in Pma1p regulation; interactions with Std1p appear to propagate [GAR+] |  |  |
| YPL036W | *PMA2* | Plasma membrane H+-ATPase, isoform of Pma1p, involved in pumping protons out of the cell; regulator of cytoplasmic pH and plasma membrane potential |  |  |
| YJR121W | *ATP2* | Beta subunit of the F1 sector of mitochondrial F1F0 ATP synthase, which is a large, evolutionarily conserved enzyme complex required for ATP synthesis; phosphorylated |  |  |
| YDL185W | *VMA1* | Subunit A of the eight-subunit V1 peripheral membrane domain of the vacuolar H+-ATPase; protein precursor undergoes self-catalyzed splicing to yield the extein Tfp1p and the intein Vde (PI-SceI), which is a site-specific endonuclease |  |  |

* Descriptions were retrieved from the *Saccharomyces* Genome Database (<https://www.yeastgenome.org/>)

**Table S6. Primers used for tagging 4×FLAG to the C-terminus of each chaperone.**

| **Description** | **Sequence (5′**-**3′)** |
| --- | --- |
| SSA1 forward | TTCCCAGGTGGTGCTCCTCCAGCTCCAGAGGCTGAAGGTCCAACCGTTGAAGAAGTTGAT ACGGCCAGTGAATTCGGCG |
| SSA1 reverse | TTATACCCAGATCATTAAAAGACATTTTCGTTATTATCAATTGCCGCACCAATTGGCTTA CAGTATAGCGACCAGCATT |
| SSB1 forward | GAATTGAGAAAGGCTGAAGTTGGTTTGAAGAGAGTTGTCACCAAGGCCATGTCTTCTCGT ACGGCCAGTGAATTCGGCG |
| SSB1 reverse | AAAACAAAATTTATATACAATATAAGTAATATTCATATATATGTGATGAATGCAGTCTTA CAGTATAGCGACCAGCATT |
| SSB2 forward | GAGTTGAGAAAGGCAGAAGTTGGTTTGAAGAGAGTTGTCACCAAGGCCATGTCTTCTCGT ACGGCCAGTGAATTCGGCG |
| SSB2 reverse | TAAGACATTAAAAATGAAAAATATATATATGTGTATAACCTTAACCAGAATGACATCTTA CAGTATAGCGACCAGCATT |
| HSC82 forward | GCTTCTACCGAAGCTCCAGTTGAAGAGGTTCCAGCTGACACCGAGATGGAAGAAGTTGAT ACGGCCAGTGAATTCGGCG |
| HSC82 reverse | GTAAAAATAAGTAAACAAATTTATATAATATATAAAACATGAAGGCGAAAAAAGAGATTA CAGTATAGCGACCAGCATT |
| HSP82 forward | GCATCCACCGCAGCTCCGGTTGAAGAGGTTCCAGCTGACACCGAAATGGAAGAGGTAGAT ACGGCCAGTGAATTCGGCG |
| HSP82 reverse | TACCTATACGTTATATTATGTTTTGTTTATAACCTATTCAAGGCCATGATGTTCTACCTA CAGTATAGCGACCAGCATT |
