# Supplementary material for: Disome-seq reveals widespread ribosome collisions that promote cotranslational protein folding
Source: Genome Biol. 2021 Jan 5;22:16. doi: 10.1186/s13059-020-02256-0 (PMC7784341; doi:10.1186/s13059-020-02256-0)
Supplement: Supplementary file 2 — Additional file 2. Review history. [file 13059_2020_2256_MOESM2_ESM.docx]

Review History

**First round of review**

**Reviewer 1**

**Are you able to assess all statistics in the manuscript, including the appropriateness of statistical tests used?**

Yes, and I have assessed the statistics in my report.

**Comments to author:**

Zhao et al. develop and use a variant of ribosome profiling, termed disome-seq, to sequence mRNA fragments protected by two stacked ribosomes (disomes) in yeast. Such stacked ribosomes occur as a result of ribosomal collisions, when a downstream elongating ribosome is slow decoding, leading to the next, upstream ribosome queuing closely behind it. The authors characterize the ribosomal collision sites and show that they are (1) associated with stop codons, (2) with the decoding of specific amino acids and (3) with structural and polypeptide motifs that can interact with the exit tunnel (through charge) and that form alpha-helices, which suggests a role for translational pausing in co-translational protein folding.

Moreover, unique structural information of the disome, using cryo-EM, is provided, which plausibly shows that the disomes transiently formed during translation elongation are fundamentally different from hard stalls of di-ribosomes that are resolved by the RQC pathway. Finally, mass spectrometry data shows that disomes associate with specific chaperones which likely aid in co-translational protein folding.

Of note, this work nicely complements two recently reported studies in human cell lines and zebrafish (Cell reports 2020) and in mice (on biorxiv) that have reported on a similar disome footprint sequencing approach to characterise ribosomal pause sites. The three manuscripts form a valuable trio of complementary research addressing how temporary elongation slow-down represents a so-far poorly studied, yet integral part of protein biosynthesis. Generally, this study is well done and appropriate for publication in Genome Biology. Specific issues to address before publication are given below.

Main points:

(1)	An important issue is the statistical framework used in the study. A few key points (1a to 1d) that this Reviewer deems important to clarify are listed in the following. Of note, as the study is based on overall comparably few usable disome reads (see Table S2) from biological duplicate datasets, the issue of statistical power and stochastic distortions is pertinent. As a general remark, a more detailed description of the analytical and computational methods is required in order to be able to evaluate the study better.

-Point (1a): For Figure 3B, in the calculation of the odds ratio as a pausing score, the control (denominator) was based on the mRNA-seq read counts. Base-level distribution of reads in mRNA-seq is known to be non-uniform. Can the authors rule out the possibility that such biases could significantly affect the apparent pausing scores? Such biases, if they exist, will also show up in the Mantel-Haenszel test as a strong deviation between disome- and mRNA-seq read distributions across codons. Can the authors explain/discuss the advantages/disadvantages of using mRNA-seq read counts instead of codon frequencies?

-Point (1b). Methods, page 17, states that "mRNA reads were used as the background to control for codon frequency in the gene; the 16th-18th nts of a 28-nt mRNA-seq read was considered as the A-site." Does this mean that really only 28mers were used in mRNA-seq? Since global uniquely mappable RNA-seq reads per library are only ca. 1.5 million (Table S3), the proportion of 28mers will be very small, creating the risk that coverage is low and the statistics are underpowered (significant stochastic effects). Generally, better documentation of methods that allow retracing the analytical pipeline would be helpful.

-(Point 1c): Also related to the statistical issues, it is essential that p-values are adjusted for multiple testing (Figure 4A: 21 amino acids, Figure 5A: 8000 amino-acid 3mers, Figure S5: 61^3 codon 3-mers) using appropriate tools.

-(Point 1d): In several cases, there is no mention of the N="number of events considered", e.g. Figures 4E and others. This should be added.

(2)	In the analyses where an association of disome sites with specific amino acids, motifs etc. is shown, it appears to be problematic that these are the "observed" values. What is lacking is the "expected" values (i.e. frequency), and the calculated enrichment (overrepresentation). For example, having few disome observations within alpha-helices on Pro (Figure 4D), Pro-Pro (Figure 4G), or Lys-Lys-Lys (Figure 5C) is no miracle - these amino acid motifs are anyway very much depleted from alpha-helices. Hence, the "observed" values are little relevant unless expressed as a disome-related enrichment (=normalised to the abundance of the amino acids within these areas).

In Figure 4G, for example, Pro-Pro is virtually absent in alpha-helices, but not so in gaps. But is this really an effect of the disomes?

Could it be more appropriate, for example, to compare Pro-Pro in gap vs. other "non-gap" areas (yet that are not alpha-helices either)?

(3)	The notion of "evolutionary conservation" in the examples that are shown in Figures 4C and 5D is somewhat misleading, as it is the amino acids that are conserved, but we do not know if the feature of disome accumulation is also conserved. Figure 5D, for example, shows the fusion of ubiquitin and a ribosomal protein. Both are anyway probably among the most highly conserved proteins in the organism, so then of course an individual motif (in this case the KKRKKK) is also expected to follow the trend of the rest of the protein and should be very conserved as well. This is, however, not necessarily a disome effect, but could simply have other reasons. One suggestion would be to take these sequence-conserved sites in their yeast data and look whether disome occurrence is conserved as well, using the human cell and zebrafish data from the recent Han et al. Cell Reports paper (PMID 32375038). Are there other, analogous examples that the authors could also compare cross-species?

(4)	Also on evolutionary conservation, Figure 4E: It is shown that more collisions occur on prolines that are conserved and in gaps vs. prolines that are not conserved but also in gaps. However, how does the analysis control for general conservation of the genes? Are the genes with conserved vs. non-conserved sites matched in terms of general conservation and translation efficiency, which both are expected to have a significant influence on disome quantities (see e.g. Figure S7B: more frequent ribosome collision observed in genes with higher monosome density)?

Suggestion: Could the authors look at amino acids other than Pro, in the same analysis as Figure 4E? According to the authors' model, the difference in pausing between conserved and non-conserved amino acids should be rather specific for Proline and not show generally on other residues.

(5)	I have some problems fully grasping the interpretation of the oligo-Lys enrichment in the exit tunnel (Figure 5A and S5), and the validation by Luciferase assays (Figure 5B).

First, the analytical pipeline how the pausing score is calculated for the larger motifs (20mer amino acid regions) is not really explained and thus difficult to understand. What was counted and used for the odds ratios? How is RNA-seq data integrated/used? This is much too vague.

Second, once the synthesized peptide is in the exit tunnel, the identity of the codon should not matter, but only the amino acid residue (Lys). The actual decoding event on which pausing occurs (and for which codon usage could be relevant) is downstream of the oligo-lysine tract when oligo-lysine is in the tunnel. Therefore, the statement on page 8, lines 51-54: "Ribosome collisions were strongly associated with positively charged 3-mers (e.g., RKR) when in exit tunnels; by contrast, such pausing signals were obscured in monosome-seq (Figure 5A). Triple-lysine had the strongest collision signal (Figure 5A), which was mainly contributed by the tract of the optimal codon (AAG) instead of the rare one (AAA, Figure S5)." is difficult to interpret. What is the model of the authors that would explain codon usage effects for the amino acids that are already in the exit tunnel?

Third, for the Luciferase assays, the authors use the codon usage that shows the strongest effect according to the exit tunnel pausing score, i.e. oligo(AAG). In order to discriminate whether codon usage really has an effect, or whether it is the Lysine per se, the authors should use alternative codon usages in the reporters. For example, they could compare the KKK combinations from Figure S5, and correlate the luciferase assay data with the pausing score observed on the endogenous sites. This would be a very instructive experiment.

Moreover, the authors explain (page 9, lines 7-8) that by placing the tested sites directly after the initiation codon "translational elongation replaces initiation as the rate-limiting step in protein synthesis (Chu et al., EMBO J 2014)." How about trying this same assay, but inserting the different amino acid motifs further downstream in the CDS (= AFTER Luciferase)? Would this not be a good control for the specificity of the observed effect?

Minor points:

(6)	In Figure 3C, it would be good to show a scatter plot for all codons between monosome-seq and disome-seq, as this would give an idea of whether disome footprints mainly show an amplified version of monosome footprint accumulation or have an additional, unique signature.

Similarly, it would be good to show the scatter plot/correlations for Figure S4A (Disome, comparison of 2 replicates), in order to be able to evaluate the reproducibility of the enrichment scores.

(7)	Figure 3D: In addition to the high accumulation of disomes at the STOP codon, there is an "inverse ramp" from the 5' to 3' end of the CDS. Is this real, or an artefact coming from the length normalization of the CDS? For example, if short vs. long coding sequences (which are known to have, in tendency, higher vs. lower translation efficiencies) have different disome accumulation, this could create this kind of effect as well. Could this for example come from longer queues of >2 ribosomes at the STOP codons of short coding sequence genes? As it is now, the graph is difficult to interpret. A possibility how to look at this better could be to generate metagene plots as a heatmap for each mRNA, sorted by CDS length, as was done by Preiss and colleagues in their TCP-seq Nature paper ( https://www.nature.com/articles/nature18647/gures/6 ).

(8)	It would be helpful to have more information on the 58mers vs. 61mers (and other sizes, possibly) - currently the analysis of the different footprint sizes is quite superficial.

For example, although it says on page 5, line 14-15, that "[..] the most abundant footprints in disome-seq were 58 and 59-nt, with the 58-nt peak being in-frame", this seems to be somewhat variable. Figure S2B indeed shows that the 58-59mers are the most abundant footprints in the 3-AT experiment, yet Figure S2D suggests that in the experiment in rich medium, it is rather the 61mers.

In Figure S11A the authors show the 61mers on STOP codons, but where are they otherwise in the transcriptome? Could the authors explore this a bit more?

(9)	Figure 6C, loss of Rack1 in structure. The authors are comparing their structure with a different structure from a different lab. Is this sufficiently comparable/specific? How can one be sure that the loss of Rack1, and other differences, are not caused by other changes in conditions related to structure determination? Would this warrant a discussion?

(10)	Figure 5B: y-axis labels are not properly aligned.

**Reviewer 2**

**Are you able to assess all statistics in the manuscript, including the appropriateness of statistical tests used?**

Yes, and I have assessed the statistics in my report.

**Comments to author:**

Translation elongation of a ribosome across an mRNA is a critical step of gene expression, and tools such as ribo-seq exist to quantify it. Slowdowns or stalls during elongation can cause problems for gene expression, leading to collisions between ribosomes and a loss of gene expression. To analyze such collisions, the authors undertake a method to isolate and analyze the population of ribosomes that exist in this collided two ribosome (disome) state. The manuscript reports on the authors' efforts to characterize the mRNA footprints covered by disomes, the 3D structure of collided disomes, and the associated complement of nascent peptides and chaperones.

While the techniques and observations will likely be of broad interest, esp. to the translation community, a few decisions during the execution of the experiments preclude a straightforward interpretation of the results as presented by the authors. In particular, the decision to include cycloheximide in the experiment has led to an obvious distortion of the captured disome population, manifesting in differences in their cryo-EM structure relative to published studies, as well as differences in the captured RNA footprints. While the authors take these differences as evidence "cells use regulated ribosome collisions to ensure protein homeostasis," this is not clear. Such effects should not prevent the authors' observations from being shared, but care should be taken in the interpretation and presentation of the data so as to prevent the community from also misinterpreting the data. There is historical precedence underscoring the importance of taking a more nuanced view of the data: failure to articulate several artifacts of the initial ribo-seq technique led to wasted effort by many labs over many years attempting to use and interpret the technique, eventually giving rise to modified versions of the protocol (by Lareau, Green) that yielded more robust and biologically meaningful results.

Specific major and minor points are detailed below.

Major points:

The authors include cycloheximide in their lysates, and then report a structure of two ribosomes (disome) in the cycloheximide-bound configuration (fig 6). They note this is different from prior structures (from Inada, Hegde labs). However, when both Inada and Hegde groups harvested disomes, they omitted cycloheximide from their lysates, which would be expected to allow for the stalling ribosome to exist in an alternate conformation. The structures of both Inada and Hegde groups are amenable to a more straightforward biological interpretation given the A-site-based stalls used to generate them. It is not surprising that the structure reported in the current manuscript differs, and it cannot be discounted that such differences are cycloheximide-induced artifacts or merely intermediates of RQC. To be clear, we do not deny the existence of the cycloheximide-bound structure, but the authors' interpretation of such a structure as an indication of regulatory potential of disomes is not justified.

The authors cite small differences in density up/downstream of disome sites as evidence that RQC has a small effect (fig S7C) and that most disomes resume translation, neither of which is not justified. One cannot infer the dynamics of a system from a static observation. While certainly possible, the data is also consistent with stalling itself being an inefficient and slow process, with most ribosomes transiting a stall site without inducing a disome configuration. This would yield the same result as that shown in fig S7C: slightly more ribosomes upstream relative to downstream.

A ribosome stalled at a stop codon is known to protect a 21nt footprint (the work of Colin Wu, Rachel Green), and stall the ribosome in a non-hybrid state (Ramakrishnan, Hegde structures). The authors' cycloheximide-stalled ribosomes exist in the rotated A/P-hybrid state (fig 6) and are also abundant at stop codons (fig 3). It is unclear how to reconcile fig 6 and fig 3: wouldn't a disome stalled at a stop codon result in a leading ribosome in a non-hybrid state? Wouldn't a disome stalled at a stop codon protect a smaller footprint (28+2+21nt=51) instead of the 60nt observed?

Throughout the manuscript the authors equate more disome-seq reads with more stalling. This is problematic. A recent controversy in the E. coli ribo-seq literature started with a group claiming that to detect stalled ribosomes, for it later to be shown/argued that such complexes are the result of sample preparation and unphysiological (Mohammad et al elife 2019). More reads at a location does not guarantee that such reads are not a technical artifact. In the absence of data demonstrating an interpretable outcome of such disomes, it remains a possibility that some of the sequence features outlined in fig 4 are a technical artifact. The manuscript should reflect that fact. (See also minor point below on fig 5b.)

In fig 7, it is not clear that chaperones are enriched in the disome fraction. Given the sizable difference in ribosomes between the monosome and disomes, a protein having the same number of molecules in the monosome and disome fractions would appear to be enriched (off-diagonal) in the analysis shown in fig 7b. It is possible that chaperones are in fact de-enriched in the disome fraction (relative to ribosomes), the opposite conclusion from that reached by the authors. To validate their conclusion that chaperones are enriched, the authors would need to show immunoblotting of the fractions shown in (A) (or a comparable experiment).

Minor points:

In several figures in the paper the authors puzzlingly include an interpretation of the figure in the figure itself. For example, fig 3D is titled "ribosome release is slow." While this title is intended to help the reader, in reality it presents one interpretation of the data which could be interpreted several other ways. Inclusion of these statements in the figure itself is problematic. Please remove the statements/titles in the figure from figs 1a, 1b, 2b, 2d, 3d, 4d, 4e, 5b, 5c, 5d.

In fig 1c, 2a, it is unclear whether the monosome/disome were separated via sucrose gradient prior to running a footprint gel. Was this the case? Please clarify.

The experiments done in fig 1 are exactly the same as those performed in (Guydosh & Green, Cell 2014) (ref 21). The text should reflect that. Please cite in the section "Translational pauses generate disomes from collisions of ribosomes".

It is not clear that the positional information between disome-seq and ribo-seq are comparable. Discrepancies between read densities at individual sites is cited as evidence these techniques capture different populations of ribosomes (fig 2). Such populations would differ in the 5'end of the cloned RNA fragment. As this is a site of considerable ligation bias (which the authors state), it is important to rule out the observed differences between disome and monosome-seq are not simply the result of 5'ligation biases.

The reads in the disome and monosome sequencing libraries appear to be taken at face value, without an attempt to correct for differences in raw material, sequencing depth, or mRNA expression. The raw mRNA in disomes is likely only a fraction that of monosomes (fig 1b, S1). There are also differences in sequencing depth ("uniquely mapped reads" in table S1). It's unclear the extent to which these effects factor into the numbers shown in fig 2b, 2c; a mere read count on transcripts in the absence of such corrections is not very biologically meaningful.

To study the effects of the tripeptides identified in the disome-seq reads (fig 5a), the authors create a reporter (fig 5b). However, the run of amino acids is proximal to the start codon, and would not be sufficiently far into the CDS to allow a disome to form. It would be a good idea to point this out in the text to prevent readers from being misled.

The conclusion of fig 5b ("polylysine decelerates translation elongation") is not justified. The sequence identified as most significant in fig 5a (KKK) was not tested, and the closest (KK, KKKK) have no or possibly mild effects. The larger effects seen at larger K lengths could be due to other known effects of this amino acid on degradation of the nascent peptide.

Both fig 4d, 5c show the distribution of disome-seq reads about the end of an alpha helix. What is this relative to? If the data is intended to show an enrichment, one must show a background expectation.

The interpretation of fig 7 is not justified ("ribosome pauses or collisions recruit chaperones"). Barring normalization issues (see major point, above), the data is also consistent with chaperones inducing stalling and collisions. Association between two entities (in this case, chaperones, disomes), cannot be taken as a causal relationship, nor a directional one.

It may be useful to examine the prevalence of mass spec peptides upstream v downstream of disomes, akin to what was done in fig S9B, but more generally and with respect to disome-seq reads. This analysis would strengthen the idea that the disome footprints and mass spec peptides are derived from similar complexes.

While the statistics in the manuscript was generally good, there were a few issues. Fig 4b uses pearson correlation, but the data does not fit a gaussian, so the test should be spearman correlation. Fig 4g uses a paired t-test, which again assumes the data fits a gaussian which it does not. While the p-values for these tests will change, the overall conclusions of the figure don't, so this is a minor point.

Fig 5b: the y-axis labels on the top graph appear to be misaligned.

There's a typo on page 10 line 45; fig S6 is referenced rather than fig S8.

**Authors Response**

**Point-by-point responses to the reviewers’ comments:**

Reviewer 1

Comments to author:

Zhao et al. develop and use a variant of ribosome profiling, termed disome-seq, to sequence mRNA fragments protected by two stacked ribosomes (disomes) in yeast. Such stacked ribosomes occur as a result of ribosomal collisions, when a downstream elongating ribosome is slow decoding, leading to the next, upstream ribosome queuing closely behind it. The authors characterize the ribosomal collision sites and show that they are (1) associated with stop codons, (2) with the decoding of specific amino acids and (3) with structural and polypeptide motifs that can interact with the exit tunnel (through charge) and that form alpha-helices, which suggests a role for translational pausing in co-translational protein folding.

Moreover, unique structural information of the disome, using cryo-EM, is provided, which plausibly shows that the disomes transiently formed during translation elongation are fundamentally different from hard stalls of di-ribosomes that are resolved by the RQC pathway. Finally, mass spectrometry data shows that disomes associate with specific chaperones which likely aid in co-translational protein folding.

Of note, this work nicely complements two recently reported studies in human cell lines and zebrafish (Cell reports 2020) and in mice (on biorxiv) that have reported on a similar disome footprint sequencing approach to characterise ribosomal pause sites. The three manuscripts form a valuable trio of complementary research addressing how temporary elongation slow-down represents a so-far poorly studied, yet integral part of protein biosynthesis. Generally, this study is well done and appropriate for publication in Genome Biology. Specific issues to address before publication are given below.

*Response:*

*We thank Reviewer #1 for the overall positive evaluation and constructive comments. We have revised the manuscript accordingly.*

Main points:

(1)	An important issue is the statistical framework used in the study. A few key points (1a to 1d) that this Reviewer deems important to clarify are listed in the following. Of note, as the study is based on overall comparably few usable disome reads (see Table S2) from biological duplicate datasets, the issue of statistical power and stochastic distortions is pertinent. As a general remark, a more detailed description of the analytical and computational methods is required in order to be able to evaluate the study better.

*Response:*

*We have performed additional statistical analyses and have improved the clarity in describing the analytical and computational methods. See below for detailed explanations.*

-Point (1a): For Figure 3B, in the calculation of the odds ratio as a pausing score, the control (denominator) was based on the mRNA-seq read counts. Base-level distribution of reads in mRNA-seq is known to be non-uniform. Can the authors rule out the possibility that such biases could significantly affect the apparent pausing scores? Such biases, if they exist, will also show up in the Mantel-Haenszel test as a strong deviation between disome- and mRNA-seq read distributions across codons. Can the authors explain/discuss the advantages/disadvantages of using mRNA-seq read counts instead of codon frequencies?

*Response:*

*We did not make it clear that the mRNA-seq performed in this study was similar to ribo-seq except that mRNA was sheared into <50 nucleotide fragments using high-salt buffer (rather than RNase I digestion as in ribo-seq). Such mRNA-seq was used to control for any bias generated during the preparation of short RNA sequencing libraries. It is similar to the appendant mRNA-seq libraries in Ingolia et al. Science 2009.*

*We agree with reviewer #1 that using codon frequencies as the background in the Mantel-Haenszel test is another strategy. In the revised manuscript, we also provided the A-site and P-site pausing scores using codon frequencies as the background (Figure S10, pasted below). It turns out that the pausing scores calculated using the mRNA-seq data as the background are highly correlated with those calculated using codon frequencies as the background. We discussed these results in the revised manuscript (in the section of “Caveats” Page 14).*

*(Figure S10)*

*“A third caveat is related to the background used in the Mantel-Haenszel test for calculating the pausing scores. We performed appendant mRNA-seq experiments that mRNA was sheared into <50-nt fragments using a high-salt buffer, instead of the RNase I digestion as in the monosome-seq and disome-seq experiments. The purpose of using such mRNA-seq data as the background in our analyses (Figs. 3-5) was to control for any potential ligation bias, amplification bias, or sequencing bias in the high-throughput sequencing. Nevertheless, we realized that a more direct approach was to use codon or amino-acid frequencies in each gene as the background (Figure S10a). We recalculated the A-site and P-site pausing scores using this approach, and found them virtually unchanged (Figures S10b, c), indicating that the reported pausing scores in this study are computationally robust. ”*

-Point (1b). Methods, page 17, states that "mRNA reads were used as the background to control for codon frequency in the gene; the 16th-18th nts of a 28-nt mRNA-seq read was considered as the A-site." Does this mean that really only 28mers were used in mRNA-seq? Since global uniquely mappable RNA-seq reads per library are only ca. 1.5 million (Table S3), the proportion of 28mers will be very small, creating the risk that coverage is low and the statistics are underpowered (significant stochastic effects). Generally, better documentation of methods that allow retracing the analytical pipeline would be helpful.

*Response:*

*Yes, only the 28 and 29-nt reads were used in the mRNA-seq data.*

*We did not make it clear that the mRNA-seq performed in this study was not the ordinary mRNA-seq that mRNA is fragmented into 300–400 nucleotides (and sequenced for 150 base pairs from both ends). The mRNA-seq performed in this study was specifically for short RNA fragments; in this mRNA-seq library, ~30% of the uniquely mapped reads are 28 and 29-nt reads (see Figure S3b for the length distribution of the mRNA-seq data for 3-AT treated cells). The description of the experimental procedure has been added to the revised manuscript.*

*(the second paragraph on page 25) “To control for technical bias during library preparation, appendant mRNA-seq was performed [16]. Specifically, total RNA was extracted using hot phenol, and 75 µg extracted RNA was applied to mRNA purification using the Dynabeads™ mRNA purification kit (Life Technologies, 61006). For fragmentation, 11.1 µl 10×fragmentation reagent (Thermo Fisher Scientific, AM8740) and 100 µl ddH2O were added to the purified mRNA. Fragmentation proceeded for 30 minutes at 70°C, and was aborted when 11.1 µl 10×stop solution was added. Fragmented RNA was precipitated by isopropanol overnight and was separated on a 17% (w/v) 7 M urea denaturing polyacrylamide gel. RNA fragments with the length ~28 nts were extracted from gel.”*

*(Page 26 last paragraph) “the ‘A-site’ of a 28-29 nt mRNA-seq read was defined as if it was a 28-29 nt monosome footprint.”*

-(Point 1c): Also related to the statistical issues, it is essential that p-values are adjusted for multiple testing (Figure 4A: 21 amino acids, Figure 5A: 8000 amino-acid 3mers, Figure S5: 61^3 codon 3-mers) using appropriate tools.

*Response:*

*Following this suggestion, Q-values (false discovery rates) were calculated using an R package, “qvalue”, and were presented in Figs. 4a, 5b, and 5d of the revised manuscript. Fig. 5b is pasted below as an example. Note that the difference between the P-value and the Q value was not quite visible in Fig. 4a (due to a relatively small number of tests for 20 amino acids).*

*FIg 5b*

-(Point 1d): In several cases, there is no mention of the N="number of events considered", e.g. Figures 4E and others. This should be added.

*Response:*

*The numbers of events considered (those relevant to the degrees of freedom in a statistical test) have been added to all applicable figures in the revised manuscript.*

(2)	In the analyses where an association of disome sites with specific amino acids, motifs etc. is shown, it appears to be problematic that these are the "observed" values. What is lacking is the "expected" values (i.e. frequency), and the calculated enrichment (overrepresentation). For example, having few disome observations within alpha-helices on Pro (Figure 4D), Pro-Pro (Figure 4G), or Lys-Lys-Lys (Figure 5C) is no miracle - these amino acid motifs are anyway very much depleted from alpha-helices. Hence, the "observed" values are little relevant unless expressed as a disome-related enrichment (=normalised to the abundance of the amino acids within these areas).

In Figure 4G, for example, Pro-Pro is virtually absent in alpha-helices, but not so in gaps. But is this really an effect of the disomes?

Could it be more appropriate, for example, to compare Pro-Pro in gap vs. other "non-gap" areas (yet that are not alpha-helices either)?

*Response:*

*We did not intend to show enrichment in Figs. 4d and 5c (in the initial version of the manuscript). Instead, we wanted to show that more ribosome collisions existed in gaps, where proline tends to locate. We agree that this point is likely trivial and probably also misleading in the initial presentation (in that readers may think of “enrichment”). We therefore have removed these panels (Fig. 4d, Fig. 4g, and Fig. 5c) in the revised manuscript and illustrate this point is in a new panel (Fig. 4c).*

*(Fig. 4c)*

*“We estimated the number of occurrences of each amino acid in gaps or α-helices in the yeast genome; the ratio between them indicates the propensity of an amino acid to appear in the gap. We found that this ratio could be predicted from the P-site pausing score of the amino acid in disome-seq (P = 7.8×10-4, N = 20, linear regression, Fig. 4c) but not in monosome-seq (P = 0.29, N = 20, Fig. 4c). Furthermore, the propensity to terminate an α-helical conformation of an amino acid (measured by ΔΔG) [40, 41] was also predictable from the P-site pausing score in disome-seq (P = 0.01, N = 20, linear regression, Fig. 4d) but not in monosome-seq (P = 0.38, Fig. 4d), suggesting a sequence-mediated coupling between ribosome collisions and the intrinsically disordered region.”*

(3)	The notion of "evolutionary conservation" in the examples that are shown in Figures 4C and 5D is somewhat misleading, as it is the amino acids that are conserved, but we do not know if the feature of disome accumulation is also conserved. Figure 5D, for example, shows the fusion of ubiquitin and a ribosomal protein. Both are anyway probably among the most highly conserved proteins in the organism, so then of course an individual motif (in this case the KKRKKK) is also expected to follow the trend of the rest of the protein and should be very conserved as well. This is, however, not necessarily a disome effect, but could simply have other reasons. One suggestion would be to take these sequence-conserved sites in their yeast data and look whether disome occurrence is conserved as well, using the human cell and zebrafish data from the recent Han et al. Cell Reports paper (PMID 32375038). Are there other, analogous examples that the authors could also compare cross-species?

*Response:*

*We agree with Reviewer #1 that a comparison between our disome-seq data and the disome-seq data in a second species would provide useful information about the conservation of ribosome collisions (in addition to the conservation of collision signals).*

*To this end, we present two new results with respect to the evolutionary conservation of ribosome collisions in the revised manuscript. First, we show an overall comparison between our yeast disome-seq data and the disome-seq data for human cells reported in Han et al. (2020). We found that the positions of ribosome collisions are highly conserved between these two species. Two panels (Fig. 2g-h) have been added to the revised manuscript.*

*(Figs. 2g, h)*

*“To determine if ribosomes collisions are likely functional and thereby are conserved over evolution, we retrieved the disome-seq data generated for human cells in a recent study [37]. We projected disome footprints detected in human cells to the orthologous position of the yeast genome based on the A-site amino acid of the pausing ribosome. For each ribosome collision in yeast, we estimated its distance (d) to the closest counterpart in human cells (Fig. 2g). d was equal to zero in 19.4% of yeast ribosome collisions (Fig. 2h), indicating that these yeast ribosome collisions occur at the same site in the human genome. To assess the statistical significance, we assigned ribosome collisions to random positions in human cells 10000 times, keeping the number of ribosome collisions in each gene unchanged. The fraction of conserved ribosome collisions (d = 0) in every randomized sample was smaller than what we observed in the genomes (P < 0.0001, the permutation test), indicating that the positions of ribosome collisions are evolutionarily conserved over the 1300-million-year evolution between yeast and humans [47]”*

*Second, we show an example of a conserved ribosome collision (as well as the conserved pausing sequence) in Fig. 5e. We removed the original Fig. 4c during revision.*

*(Fig. 5e)*

*“Polylysines often appear in the gap regions because the repellence among the positively charged residues prevents the formation of an α-helix [39]. As an example, polylysine-induced ribosome collision occurs in the linker between the two domains of RPS31p, the fusion protein of ubiquitin and ribosomal protein (Fig. 5e), raising the possibility that a translational pause benefits the cell by providing sufficient time for the co-translational folding of the upstream domain. Consistently, despite being in an intrinsically disordered region, these positively charged amino acids (KKRKKK) are conserved across eukaryotes (Fig. 5e). To test if the ribosome collision is also conserved, we retrieved the disome-seq data for human cells reported in a recent study [37]. The ribosome collision, with these positively charged nascent peptides in the exit tunnel, were detected in both human and yeast cells (Fig. 5e), albeit these two species have diverged since 1300 million year ago [44], suggesting a function of this inter-domain ribosome collision.”*

(4)	Also on evolutionary conservation, Figure 4E: It is shown that more collisions occur on prolines that are conserved and in gaps vs. prolines that are not conserved but also in gaps. However, how does the analysis control for general conservation of the genes? Are the genes with conserved vs. non-conserved sites matched in terms of general conservation and translation efficiency, which both are expected to have a significant influence on disome quantities (see e.g. Figure S7B: more frequent ribosome collision observed in genes with higher monosome density)?

Suggestion: Could the authors look at amino acids other than Pro, in the same analysis as Figure 4E? According to the authors' model, the difference in pausing between conserved and non-conserved amino acids should be rather specific for Proline and not show generally on other residues.

*Response:*

*We performed the analyses suggested but did not observe a strong signal unique for proline. Considering that additional data in support of evolutionary conservation are provided in the revised manuscript (Fig. 2g-h, Fig. 5e), we decided to remove this result (the original Fig. 4e) from the revised manuscript. We thank Reviewer #1 for this insightful comment.*

(5)	I have some problems fully grasping the interpretation of the oligo-Lys enrichment in the exit tunnel (Figure 5A and S5), and the validation by Luciferase assays (Figure 5B).

First, the analytical pipeline how the pausing score is calculated for the larger motifs (20mer amino acid regions) is not really explained and thus difficult to understand. What was counted and used for the odds ratios? How is RNA-seq data integrated/used? This is much too vague.

*Response:*

*We expanded the description of the analytical pipeline in the section“Ribosome collisions tend to occur when the nascent polylysine chain is in the exit tunnel” (Page 9) during revision. We also added a panel to illustrate the analytical pipeline in Fig. 5a.*

*(Fig. 5a)*

*“To test this hypothesis with disome-seq data, we calculated the exit-tunnel pausing score from the enrichment of each of the (203 =) 8000 amino-acid 3-mers in the 20-amino-acid region upstream of the P-site of the leading ribosome. Taking triple-lysine for example (Fig. 5a), a window of three amino acids slid one amino acid each step in this 21-amino-acid region, counting the number of triple-lysine or the total number of all other 3-mers for each pausing site; these numbers were added together for all pausing sites of a gene. The odds ratio was estimated for each gene with the corresponding numbers in the appendant mRNA-seq data as the background. The exit-tunnel pausing score was defined as the common odds ratio across the genome aggregated by the Mantel-Haenszel test”*

Second, once the synthesized peptide is in the exit tunnel, the identity of the codon should not matter, but only the amino acid residue (Lys). The actual decoding event on which pausing occurs (and for which codon usage could be relevant) is downstream of the oligo-lysine tract when oligo-lysine is in the tunnel. Therefore, the statement on page 8, lines 51-54: "Ribosome collisions were strongly associated with positively charged 3-mers (e.g., RKR) when in exit tunnels; by contrast, such pausing signals were obscured in monosome-seq (Figure 5A). Triple-lysine had the strongest collision signal (Figure 5A), which was mainly contributed by the tract of the optimal codon (AAG) instead of the rare one (AAA, Figure S5)." is difficult to interpret. What is the model of the authors that would explain codon usage effects for the amino acids that are already in the exit tunnel?

*Response-1:*

*We fully agreed with Reviewer #1 that the codon identity should not matter. The reason that we mentioned codon optimality (AAG vs. AAA) in the initial submission was that it had been suggested that the reading-through of stop codons could result in the synthesis of polylysines, which further activates the RQC pathway (Chandrasekaran et al., Nat Struct Mol Biol, 2019). We have streamlined these sentences in the revised manuscript.*

*“It has been suggested that the reading-through of stop codons could result in the translation of poly(A) tails into polylysine, which activates the RQC pathway [42]. As a result, A-tracts are often used to induce RQC in heterologous reporter systems [10]. Since that AAA is the non-optimal codon of lysine [43], it is unclear if its poor codon optimality also participates in promoting translational pausing in these experiments”*

*Response-2:*

*We also clarified during revision that the usage of synonymous codons of lysine did not participate in ribosome collisions (Page 10, paragraph 1). See also our response to the next comment.*

*“…lysine residues, instead of the non-optimal AAA codons, are accountable for the ribosome collisions in A-tracts.”*

Third, for the Luciferase assays, the authors use the codon usage that shows the strongest effect according to the exit tunnel pausing score, i.e. oligo(AAG). In order to discriminate whether codon usage really has an effect, or whether it is the Lysine per se, the authors should use alternative codon usages in the reporters. For example, they could compare the KKK combinations from Figure S5, and correlate the luciferase assay data with the pausing score observed on the endogenous sites. This would be a very instructive experiment.

*Response:*

*We did not make it clear. We agreed that synonymous codon should not affect translational pauses induced by the positively charged peptides in exit tunnels. We detected the strongest signal for AAGAAGAAG because it is the most abundant sequence encoding triple-lysine. We have clarified this point in the revised manuscript (Page 10, paragraph 2).*

*(Fig. 5d)*

*“This observation is not unexpected because it is the identity of amino acids rather than the identity of synonymous codons that should matter once the nascent peptide is in the exit tunnel (Figs. 5a, b). Consistently, when we separately estimated the pausing scores for the eight synonymous codon combinations encoding triple-lysine (Fig. 5d), endogenous ribosome collisions induced by triple-lysine were mainly contributed by triple-AAG (Fig. 5d), the most abundant codon combination for triple-lysine in the yeast transcriptome (Fig. 5d)”*

Moreover, the authors explain (page 9, lines 7-8) that by placing the tested sites directly after the initiation codon "translational elongation replaces initiation as the rate-limiting step in protein synthesis (Chu et al., EMBO J 2014)." How about trying this same assay, but inserting the different amino acid motifs further downstream in the CDS (= AFTER Luciferase)? Would this not be a good control for the specificity of the observed effect?

*Response:*

*We thank Reviewer #1 for the advice on the design of the dual-luciferase reporter. Reviewer #2 also had concerns about the location of the inserted sequences in this assay because ribosome collisions are unlikely to occur in the 5′-regions of open reading frames. Therefore, we redesigned our dual-luciferase reporter assay. We decided to insert the sequence variants between two luciferases to avoid such effect at the 5′-end of an open reading frame, as well as to avoid the interference from the termination-induced ribosome collisions at the 3′-end of an open reading frame. We did not detect significant differences among eight synonymous variants encoding for triple-lysine, indicating that it was lysine per se, instead of synonymous codons, that resulted in the translational pauses. A new panel has replaced the original design in the revised manuscript (Fig. 5c).*

*(Fig. 5c)*

*“To experimentally determine if combinations of synonymous codons for triple-lysine have different efficacies to induce translational pauses, we constructed a dual-luciferase reporter system by inserting individual synonymous variants of triple-lysine between the Renilla and firefly-luciferases (Fig. 5c). A reduction in the firefly/Renilla luciferase-activity ratio can report translational pausing induced by the inserted sequence — translational elongation replaces initiation as the rate-limiting step in protein synthesis of the downstream luciferase. We did not detect significant differences in the firefly/Renilla ratio among the eight synonymous codon combinations of triple-lysine (P = 0.57, analysis of variance, Fig. 5c). In contrast, the firefly/Renilla ratio was significantly reduced when triple-lysine was inserted (P = 8×10-4, the two-tailed t-test, Fig. 5c), suggesting that lysine residues, instead of the non-optimal AAA codons, are accountable for the ribosome collisions in A-tracts.”*

Minor points:

(6)	In Figure 3C, it would be good to show a scatter plot for all codons between monosome-seq and disome-seq, as this would give an idea of whether disome footprints mainly show an amplified version of monosome footprint accumulation or have an additional, unique signature.

Similarly, it would be good to show the scatter plot/correlations for Figure S4A (Disome, comparison of 2 replicates), in order to be able to evaluate the reproducibility of the enrichment scores.

*Response:*

*Thanks for the suggestion. We have updated Fig. 3c (now Fig. 3d) and Figure S4a accordingly.*

*(Fig. 3d)*

*(Figure S4a)*

*We also modified/added some figures using the same presentation in Fig. 3c, Fig. 4b, Figure S4b, Figure S10, and Figure S13c, e, f. For example, Fig. 3c (see below) was added during revision to show that the pausing score defined in this study can be used to identify the ribosome collisions induced by the two histidine codons under the 3-AT treatment. It serves as a positive control.*

*(Fig. 3c)*

(7)	Figure 3D: In addition to the high accumulation of disomes at the STOP codon, there is an "inverse ramp" from the 5' to 3' end of the CDS. Is this real, or an artefact coming from the length normalization of the CDS? For example, if short vs. long coding sequences (which are known to have, in tendency, higher vs. lower translation efficiencies) have different disome accumulation, this could create this kind of effect as well. Could this for example come from longer queues of >2 ribosomes at the STOP codons of short coding sequence genes? As it is now, the graph is difficult to interpret. A possibility how to look at this better could be to generate metagene plots as a heatmap for each mRNA, sorted by CDS length, as was done by Preiss and colleagues in their TCP-seq Nature paper (https://www.nature.com/articles/nature18647/gures/6 ).

*Response:*

*We thank Reviewer #1 for pointing out the phenomenon of such “inverse ramp” and for providing a potential explanation (the ribosome queue at the stop codons). We agree with this explanation and also provide another explanation. Discussion on this phenomenon has been added to the revised manuscript (in the section “The absence of translational ‘ramp’” Pages 19-20). A new supplementary figure (Figure S14) is also added. Figure S14c showed that the “inverse ramp” was indeed steeper in shorter genes.*

*(Figure S14)*

*“Instead, we observed more ribosome collisions downstream of a transcript (Fig. 3e), an “inverse ramp” that in our view can be partly explained by two mutually non-exclusive mechanisms. First, ribosomes tend not to collide in the upstream region of an mRNA due to a steric effect. The small subunit occupies ~37 nts of mRNA during subunit joining, with 22 nts downstream of the start codon [64]. This conformation requires that the 5′-end of the leading ribosome at least 24-nt downstream of the start codon; when translation elongation starts, the start codon is at the P-site of the ribosome, indicating that such ribosomes occupy 13 nts downstream of start codons (Figure S14a). Therefore, such a steric effect sets a minimum 11-nt distance between two ribosomes at translation initiation, reducing the possibility of ribosome collisions in the 5′-region of coding sequences. For example, even if the leading ribosome stays still, it requires the trailing ribosome three moves for a collision (Figure S14a). Consistent with this model, disomes were lost entirely in the first nine nts of the open reading frame of any gene (Figure S14b). The collisions propensity increases gradually along the coding sequence, reaching a plateau ~50 nts after translation starts, as indicated by the meta-gene analysis of the disome-seq data (Figure S14b).*

*A second explanation is that translation termination is relatively slow compared to elongation, resulting in ribosomal “traffic jam” upstream of stop codons; abundant disomes (Fig. 3e), and even some trisomes were formed close to stop codons (Figure S12c). It can be reasonably generalized that the elevated ribosome density due to slow termination can result in more frequent ribosome collisions in a region longer than the ~90 nts that are occupied by trisomes.*

*Note that both steric effects aforementioned, ribosomes lining up equidistantly downstream of start codons and queueing upstream of stop codons for translation termination and recycling, should have greater impacts on shorter genes, for two reasons. First, ribosomal flux is generally higher in shorter genes [32], amplifying both steric effects. Second, the same affected genomic region (e.g., a 90-nt region occupied by trisomes) will take up a larger fraction of the coding sequence in short genes. Consistently, the inverse ramp appears to be steeper among genes with shorter coding sequences (Figure S14c).”*

(8)	It would be helpful to have more information on the 58mers vs. 61mers (and other sizes, possibly) - currently the analysis of the different footprint sizes is quite superficial.

For example, although it says on page 5, line 14-15, that "[..] the most abundant footprints in disome-seq were 58 and 59-nt, with the 58-nt peak being in-frame", this seems to be somewhat variable. Figure S2B indeed shows that the 58-59mers are the most abundant footprints in the 3-AT experiment, yet Figure S2D suggests that in the experiment in rich medium, it is rather the 61mers.

In Figure S11A the authors show the 61mers on STOP codons, but where are they otherwise in the transcriptome? Could the authors explore this a bit more?

*Response:*

*Thanks for these insightful comments. We discuss in detail the length of disome footprints in the revised manuscript (in the sections “The 61-nt disome footprints” and “The 53-nt disome footprints”). We modified/added two supplementary figures (Figures S12-13) in the revised manuscript.*

*Quick answers to these questions. The genomic distributions of 58-nt and 61-nt disome footprints are very alike; they are enriched at the stop codon (similar to the 61-nt footprints in Han et al. Cell Rep 2020). The 53-nt disome footprints are less abundant; they distributed more evenly along an mRNA (similar to the 52 and 54-nt footprints in Han et al.). The 53-nt disome footprints became more abundant relative to the 61-nt disome footprints upon 3-AT treatment, likely due to longer decoding time (the A-site of the leading ribosome is open). The 58-nt disome footprints also became more abundant relative to the 61-nt disome footprints upon 3-AT treatment, likely due to a longer time for the trailing ribosome to make another move. Please read the pasted supplementary figures and discussion below for detailed information.*

*(Figure S12, discussed on Pages 15-16)*

*“Although not highlighted in our results, note that we did conduct additional analyses to examine the length distribution of disome footprints. In addition to the 58-nt disome footprints whose conformation was determined in Fig. 1e as two 28-nt monosome footprints spaced by two nts, the 5′-end of the 61-nt fragments also exhibited an apparent 3-nt periodicity (Figs. 1d, 2b), suggesting the presence of a second disome conformation. The disomes accumulated at the stop codons (Figure S12a) indicate that the 61-nt disome footprints were composed of two 28-nt monosome footprints spaced by five nts. The fate of such 61-nt disomes is likely determined by which of the two events occurs first. If the leading ribosome resumes translation first, the ribosome collision is resolved; alternatively, if the trailing ribosome moves first, probably because the leading ribosome has paused for a long time, a 58-nt disome is generated.*

*The 61-nt disome footprints were more abundant than the 58-nt disome footprints in cells growing in the rich medium (Fig. 2b, Figure S13a). The abundance of the 61-nt disome footprints was reduced when cells are treated with 3-AT (Fig. 1d, Figure S13a), a phenomenon that was also observed in a recent study [56]. We speculate that the translational pauses at the histidine codons induced by the 3-AT treatment last longer than the time frame of an average translational pause for yeast cells growing in the rich medium, thereby providing additional time for one more move of the trailing ribosome toward the leading ribosome.*

*To determine if the distinction between the 58-nt and the 61-nt disome footprints can provide additional information for the strength of a ribosome collision, we separately estimated the pausing scores from the 58-nt or the 61-nt disome footprints. They were highly correlated (for A-site, ρ = 0.92, P < 2.2×10-16, N = 64; for P-site, ρ = 0.88, P = 3×10-7, N = 20, Spearman’s correlations, Figure S12b), indicating the absence of length-specific collision signals between the 58-nt and the 61-nt disome footprints. To improve the statistical power, we combined the 58 and 61-nt footprints for the analyses in Figs. 3-5. A recent investigation on mouse liver also reported two lengths of disome footprints, 59-60 nts and 62-63 nts; similar to our observations, nearly identical pausing sequences were reported for the disome footprints of these two lengths [57].”*

*(Figure S13, discussed on Pages 16-18)*

*“In addition to the 58-nt and the 61-nt disome footprints, we observed the 53-nt disome footprints, which showed an apparent 3-nt periodicity (Figs. 1d, 2b) and accounted for 10.9% of disome footprints for yeast cells growing in the rich medium (Figure S13a). This indicates the existence of a third disome conformation. When treated with 3-AT, the 5′-end of most 53-nt disome footprints was 45-nt upstream of the histidine codons (Figure S13b), the same position as in the 58-nt disome footprints (Fig. 1e). It indicates that the positions of the A-site of the leading ribosome relative to the 5′-end is identical between the 53-nt and the 58-nt footprints. This observation echoes the two lengths of monosome footprints reported in previous studies, ~21 nts and ~28 nts [51, 53]; these two footprints share the same 5′-end but show different 3′-end. The 28-nt footprints represent the ribosomes whose A-sites are occupied (in the rotated state with hybrid A/P and P/E tRNAs or the non-rotated state with A/A and P/P tRNAs); the length of footprints reduces to ~21 nts when the A-site of a ribosome is open (in the non-rotated state with P/P and E/E tRNAs) [53].*

*In alignment with the two lengths of monosome footprints, we speculate that the (28+2+23=) 53-nt disome footprints represent a disome conformation that the A-site of the leading ribosome is open, whereas the (28+2+28=) 58-nt disome footprints represent a conformation that the A-site of the leading ribosome is occupied. If true, we predict that the abundance of the 53-nt disome footprints will increase when cells are treated with 3-AT; it is because 3-AT induces ribosome collisions mainly through an elongated time frame for decoding histidine codons, the very state that the A-site of the leading ribosome is open. The proportions of the 53-nt footprints indeed increased from 10.9% for cells growing in the rich medium to 17.9% for cells treated with 3-AT (Figs. 1d, 2b, Figure S13a), in support of our interpretation of the 53-nt disome footprints.*

*If the 53-nt disome footprints represent a disome conformation that the A-site of the leading ribosome is open, we further predict the existence of disome structure that the leading ribosome is in the non-rotated state with P/P and E/E tRNAs (presumably protecting 23-nt mRNA fragment) and the trailing ribosome is in the rotated state with hybrid A/P and P/E tRNAs (protecting 28-nt mRNA fragment). Indeed, we observed that such conformation in (4908/35918=) 13.7% of the disomes collected from yeast cells growing in the rich medium (Figure S11d). Nevertheless, the specific contacts between the two 40S subunits observed in di-ribosomes [9, 46] remained absent in these disomes (Figure S11e).*

*For cells growing in the rich medium, the stop codons were less representative in the A-site of leading ribosomes in the 53-nt disome footprints (Figures S13c, d), echoing the 52-nt and 54-nt disome footprints recently reported in human cells [37]. In the study, the 52-nt and 54-nt footprints were protected by the disomes that the A-site of the leading ribosome was open and tended to locate in coding sequences compared to the stop codon. On the other hand, the 58 and 61-nt disome footprints detected in our study are similar to the ~61-nt footprints reported in human cells; they showed a stronger tendency to locate at stop codons [37]. The reduced enrichment of the 53-nt disome footprints at stop codons (Fig. 3e, Figures S13c, d) suggests that stop codons are rapidly occupied by release factors in the A-site of ribosomes; the translation is slow at stop codons probably because of the prolonged subsequent steps disassembling the post-termination ribosomal complexes [58].*

*If the 53-nt and the 58-nt/61-nt footprints are protected by disomes that the A-site of the leading ribosome is open and occupied, respectively, we reasoned that the 58-nt/61-nt disome footprints should perform better than the 53-nt ones in detecting the translational pauses associated with peptide bond formation and translocation. It is because these processes slow down translation elongation only if the A-site of a ribosome is occupied. Indeed, when calculated from the 53-nt disome footprints, the P-site pausing score of proline, the amino acid that is well known as a poor acceptor for peptide bond formation [44, 45], was no longer significantly >1; such pausing signal was observed for the 58-nt disome footprints (Fig. 4a, Figure S13e).*

*We further reasoned that the 53-nt disome footprints should perform better than the 58-nt ones in detecting the effect of codon optimality, because codon optimality causes translational pauses only if the A-site of a ribosome is open [53]. However, we did not observed more ribosome collisions associated with non-optimal codons than optimal codons using either the 53-nt disome footprints (y-axis in Figure S13c, P = 0.47, N = 61, Mann-Whitney U test) or the 58-nt/61-nt disome footprints (x-axis in Figure S13c, P = 0.85, N = 61, Mann-Whitney U test). In fact, the A-site pausing score for the 61 amino-acid coding codons was highly correlated between the 53-nt and the 58-nt/61-nt disome footprints (Figure S13c, ρ = 0.56, P = 3.5×10-6, N = 61, Spearman’s correlation). Our observation is consistent with Han et al., who reported in human cells that synonymous codon usage/tRNA supply was poorly associated with the propensity for ribosome collisions [37].*

*Synonymous codons are known to be recognized at variable rates [18, 19, 53], which further regulate mRNA stability [30, 59]. The impact of codon optimality on ribosome collisions was not detected in disome-seq. One explanation is that decoding is a relatively rapid step in the work cycle of translation elongation [60]. Therefore, its potential in inducing variable ribosome collisions is masked by variation in the duration of other steps such as peptide bond formation. Consistently, when the decoding time became dominant in the work cycle of translation elongation as the yeast cells were treated with 3-AT, the non-optimal codon of histidine, CAT, did induce more ribosome collisions than the optimal codon, CAC (Fig. 1e, Figures S13b, f).”*

(9)	Figure 6C, loss of Rack1 in structure. The authors are comparing their structure with a different structure from a different lab. Is this sufficiently comparable/specific? How can one be sure that the loss of Rack1, and other differences, are not caused by other changes in conditions related to structure determination? Would this warrant a discussion?

*Response:*

*RACK1 exists in both leading and trailing ribosomes of our disome structures. It is the interaction (observed in the RQC-triggering di-ribosomes) between two RACK1 that is absent, likely due to a different orientation of the two 80S ribosomes in the disome. We followed the standard image processing procedures, which were not likely to introduce bias during the structural determination. Therefore, we believe that the structural differences between disomes and di-ribosomes are computational robust.*

*More importantly, in the revision, we have followed the suggestion of Reviewer #2 to repeat the structural determination using disomes obtained in the absence of cycloheximide, as what has been done for the structural determination of di-ribosomes in previous papers. The resulting disome structure is essentially the same as the one obtained with cycloheximide in the lysate—both the leading and the trailing 80S ribosomes were in the rotated state (hybrid A/P and P/E tRNAs). Fig. 6 has been updated with the structure of this cycloheximide-omitted disome.*

*We also discussed the effect of experimental procedures on structural determination (Page 15, Paragraph 3).*

*“Similarly, it remains unclear how the chemicals in the lysis buffer could have affected the structural determination of disomes by cryo-EM. In addition to the disomes collected with cycloheximide omitted from the lysate (Fig. 6, Figure S6), we also determined the structure of the disomes extracted with cycloheximide added to the lysis buffer (as in the disome-seq experiment). These disomes held the two structural features reported in Fig. 6: a markedly different interface from the di-ribosome and a rotated state for the leading ribosome (Figure S11a-c). Nevertheless, the structural determinations of disomes under various experimental conditions are recommended in the future for a more comprehensive understanding of ribosome collisions.”*

(10)	Figure 5B: y-axis labels are not properly aligned.

*Response:*

*This panel has been replaced by the results of a new dual-luciferase experiment. We have checked the labeling for all figures in the revised manuscript.*

**Reviewer 2**

**Comments to author:**

Translation elongation of a ribosome across an mRNA is a critical step of gene expression, and tools such as ribo-seq exist to quantify it. Slowdowns or stalls during elongation can cause problems for gene expression, leading to collisions between ribosomes and a loss of gene expression. To analyze such collisions, the authors undertake a method to isolate and analyze the population of ribosomes that exist in this collided two ribosome (disome) state. The manuscript reports on the authors' efforts to characterize the mRNA footprints covered by disomes, the 3D structure of collided disomes, and the associated complement of nascent peptides and chaperones.

While the techniques and observations will likely be of broad interest, esp. to the translation community, a few decisions during the execution of the experiments preclude a straightforward interpretation of the results as presented by the authors. In particular, the decision to include cycloheximide in the experiment has led to an obvious distortion of the captured disome population, manifesting in differences in their cryo-EM structure relative to published studies, as well as differences in the captured RNA footprints. While the authors take these differences as evidence "cells use regulated ribosome collisions to ensure protein homeostasis," this is not clear. Such effects should not prevent the authors' observations from being shared, but care should be taken in the interpretation and presentation of the data so as to prevent the community from also misinterpreting the data. There is historical precedence underscoring the importance of taking a more nuanced view of the data: failure to articulate several artifacts of the initial ribo-seq technique led to wasted effort by many labs over many years attempting to use and interpret the technique, eventually giving rise to modified versions of the protocol (by Lareau, Green) that yielded more robust and biologically meaningful results.

*Response:*

*We thank Reviewer #2 for kindly reminding us of the misinterpretations of the ribo-seq data that have taken place in the past ten years or so. We fully agree with Reviewer #2 that due caution is required in interpreting the data presented in this study. We have revised our manuscript accordingly. We performed additional cryo-EM experiments that cycloheximide is omitted, and we discussed alternative interpretations of the data.*

Specific major and minor points are detailed below.

Major points:

The authors include cycloheximide in their lysates, and then report a structure of two ribosomes (disome) in the cycloheximide-bound configuration (fig 6). They note this is different from prior structures (from Inada, Hegde labs). However, when both Inada and Hegde groups harvested disomes, they omitted cycloheximide from their lysates, which would be expected to allow for the stalling ribosome to exist in an alternate conformation. The structures of both Inada and Hegde groups are amenable to a more straightforward biological interpretation given the A-site-based stalls used to generate them. It is not surprising that the structure reported in the current manuscript differs, and it cannot be discounted that such differences are cycloheximide-induced artifacts or merely intermediates of RQC. To be clear, we do not deny the existence of the cycloheximide-bound structure, but the authors' interpretation of such a structure as an indication of regulatory potential of disomes is not justified.

*Response-1:*

*We thank Reviewer #2 for raising the possibility that cycloheximide could have impacted the conformation of disome structure. To solve this problem, we performed one additional cryo-EM experiment using disomes collected in the absence of cycloheximide (neither in the culturing media nor in the lysis buffer). Fig. 6 and Figures S6 have been updated with the new cryo-EM results.*

*(updated Fig. 6 is pasted here)*

*It turned out that disomes collected with cycloheximide omitted from the lysate held the same structural features as we initially reported for disomes collected from the cycloheximide-containing lysate. 1) the interface between two ribosomes in our disomes was markedly different from that in the previously reported di-ribosomes; 2) a majority (86%) of the leading 80S ribosomes in disomes are in the rotated conformation with hybrid A/P and P/E tRNAs. These observations suggest that the differences between disome and di-ribosome structure are not artifacts induced by cycloheximide.*

*(Pages 11-12)*

*“To test if the disomes collected from endogenous genes in fast-proliferating cells are structurally identical to the RQC-inducing di-ribosomes [9, 46], we performed cryo-EM analyses of disomes collected after RNase I digestion. Following the previous protocols for the structural determination of di-ribosomes or tri-ribosomes [9, 15, 46], we omitted the elongation inhibitor cycloheximide in the lysis buffer. Disomes showed a structure of two ribosomes linked by a bent mRNA, and therefore, two 40S subunits facing towards each other (Fig. 6a). Furthermore, the 5′-trailing ribosomes were detected in the rotated state that harbors hybrid A/P and P/E tRNAs (Fig. 6a), indicating that they cannot further translocate, likely due to the road-blocking leading ribosomes. These observations are in general consistent with the structure of di-ribosomes induced by hard stalls in cell-free systems [9, 15, 46].*

*However, two distinct features indicate the essential difference between disomes and di-ribosomes. First, the relative orientation between the two ribosomes in the disome was different from that in the RQC-triggering di-ribosome (Fig. 6b). It was reported that the two ribosomes in the di-ribosome form a large interface with highly specific contacts between two 40S subunits (Figure S6a), which was considered to be a feature recognizable by the RQC initiation factors such as Hel2p [9, 11, 46, 47]. However, in the disome, the interface between the two ribosomes was relatively flexible; the contacts between the two ribosomes were much weaker or nearly absent in our structure of the disome (Fig. 6c, Figures S6b-h). For instance, a tight interaction was reported between the two RACK1 (Asc1p) — the protein involved in the initiation of RQC — from the two ribosomes in the di-ribosome [9]. However, the interaction was completely lost in the disome (Figs. 6d, e), due to the different orientation of the two 40S subunits (Fig. 6b). These observations suggest that the disome may lack specific structural features required for recruiting RQC factors, such as Hel2p.*

*Second, the leading ribosome of the majority (31010/35918 = 86.3%) of disomes was detected in the rotated state (Fig. 6a), in sharp contrast to the non-rotated state (with P/P and E/E tRNAs) reported for the leading ribosome in di-ribosomes. The non-rotated state has been previously reported as a feature of stalled 80S ribosomes before collisions [9], and therefore, the rotated state of the leading ribosome indicates that these disomes are likely induced by transient pauses. Collectively, the results of cryo-EM analyses indicate that the widespread disomes observed in vivo (Fig. 2c) are structurally different from di-ribosomes that are recognized and resolved by the RQC pathway.”*

*Response-2:*

*At any rate, we fully agree with Reviewer #2 that we should interpret the data with care. We added a section “Caveats” in the Discussion of the revised manuscript, including sentences discussing how cycloheximide (and other treatments) may affect the interpretations of the ribosome structures. (Page 15, Paragraph 3)*

*“Similarly, it remains unclear how the chemicals in the lysis buffer could have affected the structural determination of disomes by cryo-EM. In addition to the disomes collected with cycloheximide omitted from the lysate (Fig. 6, Figure S6), we also determined the structure of the disomes extracted with cycloheximide added to the lysis buffer (as in the disome-seq experiment). These disomes held the two structural features we reported in Fig. 6: a markedly different interface from the di-ribosome and a rotated state for the leading ribosome (Figure S11a-c). Nevertheless, the structural determinations of disomes under various experimental conditions are recommended in the future for a more comprehensive understanding of ribosome collisions.*

*Response-3:*

*With these revisions, we have determined the structure of endogenous ribosome collisions in fast proliferating wild-type cells, with cycloheximide omitted from the lysate. We believe that our disome structure can serve as a good complement to the di-ribosome structures reported in previous studies (Juszkiewicz et al. Mol Cell 2018, Ikeuchi et al. EMBO J 2019). These di-ribosomes were often induced by strong stalling signals (e.g., eRF1-AAQ or CGACCGCGACCG) that are uncommon in the wild-type cells.*

*Response-4:*

*We cannot rule out the possibility that Reviewer #2 raised—the disomes reported in this study are possible intermediates toward triggering the RQC pathway. Actually we think that they are. We discussed this possibility in the revised manuscript (Fig. 7e, Page 22).*

*(Fig. 7e)*

*“There are at least three possible fates for these ribosome collisions observed in this study. Firstly, some ribosome collisions are likely transient, and the translation of both ribosomes can be resumed (Fig. 7e, top path); this could be the mechanism that keeps the ribosome density largely constant over a transcript (Fig. 3e, top panel, Figure S14c) in spite of widespread collided ribosomes (~5.8% at the moment that the disome “snapshot” was taken, Figs. 1b; or ~10% estimated in mouse liver [57]). Second, some ribosome collisions may last for a more extended period; these collisions may promote co-translational protein folding or aid in targeting to correct subcellular localization [57, 70] (Fig. 7e, middle path). Third, if ribosome collisions last for a sufficiently long time, the leading ribosomes shift to the non-rotated stalling state, and a more rigid interface between the collided ribosomes triggers the recognition by the RQC pathway, leading to the degradation of mRNA and nascent peptides [9, 15, 37, 46, 56] (Fig. 7e, bottom path).”*

The authors cite small differences in density up/downstream of disome sites as evidence that RQC has a small effect (fig S7C) and that most disomes resume translation, neither of which is not justified. One cannot infer the dynamics of a system from a static observation. While certainly possible, the data is also consistent with stalling itself being an inefficient and slow process, with most ribosomes transiting a stall site without inducing a disome configuration. This would yield the same result as that shown in fig S7C: slightly more ribosomes upstream relative to downstream.

*Response:*

*In this study, we reasoned that some disomes might be able to resume translation after a transient pause, mainly from seven observations. First, ~6% of 80S ribosomes were trapped in disomes in cells growing in the rich medium (Fig. 1b). Second, ribosome collisions were widespread, on nearly half of translated genes (Fig. 2c). Third, the propensity for ribosome collision on a gene was positively correlated with the ribosome density on the gene (Fig. 2d). Fourth, the ribosome density was very high in some genes (e.g., three ribosomes in a 100-nt mRNA region, Figure S5), as inferred from the data reported in a previous study (Arava et al. 2003 PNAS). Based on these four observations, we speculated that it would be too costly for cells to degrade each mRNA molecule with collided ribosomes, especially when collision occurs more frequently on the mRNA with higher ribosome density. Fifth, the structures of disomes and di-ribosomes were different (Fig. 6). Sixth, there was only a slight reduction in ribosome density downstream of a disome peak (Figure S7c in the initial submission). Seventh, disomes tended to be associated with some specific chaperones (Fig. 7). It is worth noting that the first three phenomena were also observed by recent studies in humans and zebrafish (Han et al. 2020 Cell Rep), or in mice (Arpat et al. 2020 Genome Res).*

*We agree with Reviewer #2 that the evidence showing in Figure S7c (the sixth evidence above) has alternative interpretations, and have removed it during revision.*

*We also agree with reviewer #2 that the fraction of resumable ribosome collisions requires further investigation. We clearly stated this view in the revised manuscript (Page 22, last paragraph).*

*“It is not yet clear the determinants of the collision duration, the mechanisms by which cells can sense how long a ribosome collision has persisted, and the fraction of ribosome collisions destined for each of the three fates.”*

A ribosome stalled at a stop codon is known to protect a 21nt footprint (the work of Colin Wu, Rachel Green), and stall the ribosome in a non-hybrid state (Ramakrishnan, Hegde structures). The authors' cycloheximide-stalled ribosomes exist in the rotated A/P-hybrid state (fig 6) and are also abundant at stop codons (fig 3). It is unclear how to reconcile fig 6 and fig 3: wouldn't a disome stalled at a stop codon result in a leading ribosome in a non-hybrid state? Wouldn't a disome stalled at a stop codon protect a smaller footprint (28+2+21nt=51) instead of the 60nt observed?

*Response:*

*Two types of ribosome pausing/stalling at a stop codon need to be distinguished. Both types can be observed in Wu et al. Mol Cell 2019, so we start from their Figure 1E (see below). Type I was related to the ribosome footprints observed in the wild-type cells (red circle); it protected 28-nt footprints likely because the A-site of ribosomes was occupied by a release factor. Type II was related to the ribosome footprints observed when the release factor eRF1 was depleted (green circle); it protected 21-nt footprints likely because the A-site of ribosomes was not occupied by a release factor.*

*Type I pausing/stalling occurs in the wild-type and therefore, is more relevant to the endogenous ribosome collisions in the wild-type fast proliferating cells, which are the focus of our study.*

*We did not co-culture cells with cycloheximide; we added cycloheximide to the lysis buffer as performed in recent ribo-seq studies, including Wu et al. Mol Cell 2019. We observed the 28-nt monosome footprints at the stop codon (yellow circle below, from Figure S12a of our manuscript) as Wu et al. Mol Cell 2019, and we observed an amplified signal in the disome-seq data. The ribosome collision associated with the type I pausing/stalling—the (28+2+28) = 58-nt or the (28+5+28) = 61-nt footprints—were enriched at the stop codon (blue circle below). Such footprints, in our view, are fundamentally different from the 21-nt footprints (Type II pausing/stalling) observed in the eRF1-depleted cells (Wu et al. Mol Cell 2019) or under the eRF1-AAQ treatment (Juszkiewicz et al. Mol Cell 2018).*

*(Figure S12a)*

*Although cycloheximide was in the lysate for both wild-type and eRF1-depleted cells in Wu et al. Mol Cell 2019, we reason that the type I pausing/stalling at stop codons is probably not caused by cycloheximide, because the pausing/stalling at stop codons in the wild-type cells was also reported using 5PSeq with cycloheximide omitted from the lysate (Pelechano et al. Cell 2015). The slow translation at stop codons in the wild-type cells is probably due to the prolonged subsequent steps disassembling the post-termination ribosomal complexes (Prabhakar et al. Cell Rep 2017). The 80S ribosomes undergo a series of conformational changes during translation termination and recycling, making the conformations of individual intermediates hard to be identified during the 3D classification of cryo-EM.*

*We thank review #2 for pointing to the shorter disome footprints. In our study, we indeed observed shorter disome footprints (~53-nt, also showing 3-nt periodicity) that are likely protected by the disomes in which the A-site of the leading ribosome is open. More importantly, when we performed a cryo-EM experiment with cycloheximide omitted from the lysate, we collected much more disomes particles than our previous cryo-EM experiment. These disome particles allowed us to identify a new group (~14%) of disome particles during the third round of the 3D classification; the leading ribosome of these disomes is in the non-rotated state with P/P and E/E tRNAs. These new data offer us an opportunity to discuss the genomic distribution of the 53-nt disome footprints, together with the potential rotated/non-rotated status of such disomes, in the section “The 53-nt disome footprints” of the revised manuscript (Pages 16-18, Figures S11d, S13a-d pasted below).*

*(Figure S11d)*

*(Figure S13a-d)*

*“In addition to the 58-nt and the 61-nt disome footprints, we observed the 53-nt disome footprints, which showed an apparent 3-nt periodicity (Figs. 1d, 2b) and accounted for 10.9% of disome footprints for yeast cells growing in the rich medium (Figure S13a). This indicates the existence of a third disome conformation. When treated with 3-AT, the 5′-end of most 53-nt disome footprints was 45-nt upstream of the histidine codons (Figure S13b), the same position as in the 58-nt disome footprints (Fig. 1e). It indicates that the positions of the A-site of the leading ribosome relative to the 5′-end is identical between the 53-nt and the 58-nt footprints. This observation echoes the two lengths of monosome footprints reported in previous studies, ~21 nts and ~28 nts [51, 53]; these two footprints share the same 5′-end but show different 3′-end. The 28-nt footprints represent the ribosomes whose A-sites are occupied (in the rotated state with hybrid A/P and P/E tRNAs or the non-rotated state with A/A and P/P tRNAs); the length of footprints reduces to ~21 nts when the A-site of a ribosome is open (in the non-rotated state with P/P and E/E tRNAs) [53].*

*In alignment with the two lengths of monosome footprints, we speculate that the (28+2+23=) 53-nt disome footprints represent a disome conformation that the A-site of the leading ribosome is open, whereas the (28+2+28=) 58-nt disome footprints represent a conformation that the A-site of the leading ribosome is occupied. If true, we predict that the abundance of the 53-nt disome footprints will increase when cells are treated with 3-AT; it is because 3-AT induces ribosome collisions mainly through an elongated time frame for decoding histidine codons, the very state that the A-site of the leading ribosome is open. The proportions of the 53-nt footprints indeed increased from 10.9% for cells growing in the rich medium to 17.9% for cells treated with 3-AT (Figs. 1d, 2b, Figure S13a), in support of our interpretation of the 53-nt disome footprints.*

*If the 53-nt disome footprints represent a disome conformation that the A-site of the leading ribosome is open, we further predict the existence of disome structure that the leading ribosome is in the non-rotated state with P/P and E/E tRNAs (presumably protecting 23-nt mRNA fragment) and the trailing ribosome is in the rotated state with hybrid A/P and P/E tRNAs (protecting 28-nt mRNA fragment). Indeed, we observed that such conformation in (4908/35918=) 13.7% of the disomes collected from yeast cells growing in the rich medium (Figure S11d). Nevertheless, the specific contacts between the two 40S subunits observed in di-ribosomes [9, 46] remained absent in these disomes (Figure S11e).*

*For cells growing in the rich medium, the stop codons were less representative in the A-site of leading ribosomes in the 53-nt disome footprints (Figures S13c, d), echoing the 52-nt and 54-nt disome footprints recently reported in human cells [37]. In the study, the 52-nt and 54-nt footprints were protected by the disomes that the A-site of the leading ribosome was open and tended to locate in coding sequences compared to the stop codon. On the other hand, the 58 and 61-nt disome footprints detected in our study are similar to the ~61-nt footprints reported in human cells; they showed a stronger tendency to locate at stop codons [37]. The reduced enrichment of the 53-nt disome footprints at stop codons (Fig. 3e, Figures S13c, d) suggests that stop codons are rapidly occupied by release factors in the A-site of ribosomes; the translation termination is probably slow in the prolonged subsequent steps disassembling the post-termination ribosomal complexes [58].*

Throughout the manuscript the authors equate more disome-seq reads with more stalling. This is problematic. A recent controversy in the E. coli ribo-seq literature started with a group claiming that to detect stalled ribosomes, for it later to be shown/argued that such complexes are the result of sample preparation and unphysiological (Mohammad et al elife 2019). More reads at a location does not guarantee that such reads are not a technical artifact. In the absence of data demonstrating an interpretable outcome of such disomes, it remains a possibility that some of the sequence features outlined in fig 4 are a technical artifact. The manuscript should reflect that fact. (See also minor point below on fig 5b.)

*Response:*

*We fully agree with Reviewer #2 that controversies had taken place in the interpretation of ribo-seq data in various species, including E. coli. With this in mind, when we found that polylysines induced more ribosome collisions from the disome-seq data, we validated with dual-luciferase reporters (Figure 5, see our detailed response below). It is worth discussing the distinction between the abundance of disome-seq reads and translational pausing/stalling. A paragraph has been added to Discussion (in the section of Caveats, Page 15, Paragraph 2).*

*“Last, it is worth noting that the ribo-seq protocol has “evolved” since its debut in 2009 [16]. In the initial protocol, yeast cells were co-cultured with cycloheximide, a step that was later shown distorting the location of ribosome footprints within genes [18, 19, 52]. Furthermore, the addition of cycloheximide, as well as other antibiotics, in the lysis buffer will stabilize ribosomes at various conformations that protect mRNA fragments of different sizes [51, 53]. The size selection for ribosome footprints has also been shown to have unexpected effects on the identification of translational pausing sites [54]. We avoided co-culturing yeast cells with cycloheximide but added cycloheximide in the lysis buffer to stabilize ribosomes, following recently updated ribo-seq protocols [18, 19, 53, 55]. Nevertheless, it is reasonable to assume that although disome-seq represents some advance in understanding ribosome collisions, it inevitably reflects only part of the whole story, due to technical limitations of the experimental procedures.”*

In fig 7, it is not clear that chaperones are enriched in the disome fraction. Given the sizable difference in ribosomes between the monosome and disomes, a protein having the same number of molecules in the monosome and disome fractions would appear to be enriched (off-diagonal) in the analysis shown in fig 7b. It is possible that chaperones are in fact de-enriched in the disome fraction (relative to ribosomes), the opposite conclusion from that reached by the authors. To validate their conclusion that chaperones are enriched, the authors would need to show immunoblotting of the fractions shown in (A) (or a comparable experiment).

*Response-1:*

*We did not make it clear in Fig. 7b. An equal amount of total protein extracted from the monosome and the disome fractions were mixed and were applied for MS/MS. Therefore, the protein intensity reported in Fig. 7 has been normalized for “per ribosome”; proteins on the diagonal have the same abundance per ribosome. That is why most constitutive ribosome components are on the diagonal of Fig. 7b. Chaperones were mostly above the diagonal in Fig. 7b, indicating that they are enriched (per ribosome) in disomes. We have modified Fig. 7a and edited the text to improve clarity.*

*(Figs. 7a, b, see edited legends below)*

*a The schematic of detecting the disome-enriched proteins through SILAC. Two biological replicates were performed with reciprocal labeling (the left panel). Because equal amount of total protein extracted from the monosome fraction and disome fraction were mixed, proteins having the same abundance per ribosome between monosomes and disomes should be located on the diagonal (the right panel). Proteins locating above or below the diagonal should be those enriched in the disomes or the monosomes, respectively.*

*b The protein intensities per ribosome in disomes were plotted against those in monosomes. Chaperones (green) are enriched in the disome, whereas most constitutive ribosomal proteins (pink) show the same abundance per ribosome between the monosome and the disome.*

*(Page 15 paragraph 1) “Most proteins have similar abundance per ribosome in disomes and in monosomes (e.g., two copies in a disome and one copy in a monosome) and therefore, were on the diagonal in the scatter plot showing protein intensities in disomes vs. in monosomes; many were constitutive ribosome components (Fig. 7b and Figure S7a). Proteins above the diagonal line were the disome-enriched proteins (Figs. 7a, b, Figure S7a, and Tables S4, S5). Proteins involved in the RQC pathway such as Hel2p, Slh1p, Cue3p, and Rqt4p [9, 46, 48] were not observed significantly enriched in disomes (Tables S4, S5).”*

*(Methods, Page 32) “150 μg light-labeled monosome protein was mixed with 150 μg heavy-labeled disome protein; 150 μg heavy-labeled monosome protein was mixed with 150 μg light-labeled disome protein as a replicate.”*

*Response-2:*

*We agree with Review #2 that it would be useful to validate the mass spectrometry results using immunoblotting. We successfully tagged 4×FLAG for five disome-enriched chaperones, Ssa1p, Ssb1p, Ssb2p, Hsc82p, and Hsp82p, and performed immunoblotting to detect their abundance in the monosome and the disome fractions. The enrichment in the disome fraction (per ribosome) was validated with immunoblotting for each of the five chaperones (Fig. 7d, added during revision).*

*Fig 7d*

Minor points:

In several figures in the paper the authors puzzlingly include an interpretation of the figure in the figure itself. For example, fig 3D is titled "ribosome release is slow." While this title is intended to help the reader, in reality it presents one interpretation of the data which could be interpreted several other ways. Inclusion of these statements in the figure itself is problematic. Please remove the statements/titles in the figure from figs 1a, 1b, 2b, 2d, 3d, 4d, 4e, 5b, 5c, 5d.

*Response:*

*Corrected.*

In fig 1c, 2a, it is unclear whether the monosome/disome were separated via sucrose gradient prior to running a footprint gel. Was this the case? Please clarify.

*Response:*

*We clarified in the revised manuscript that we omitted the sucrose-gradient step when constructing the disome-seq and monosome-seq libraries (Page 24, last paragraph).*

*“Our pilot experiments, as well as previous studies, have shown that sucrose gradient centrifugation is not necessary [55, 71-73] as long as the digestion with RNase I is complete (Figure S1); rather, this step consumes a large amount of ribosome (especially disome) samples. The following-up computational analyses can help to tell if the RNA fragments being collected are largely protected by ribosomes: whether the fragments are restricted to the coding sequences, whether the fragments are enriched in certain lengths, and whether a 3-nt periodicity exists (Figures S3c-f). Therefore, RNA was directly extracted from the solution for RNase digestion with hot phenol and was separated on a 17% (w/v) 7 M urea denaturing polyacrylamide gel in a 0.5×Tris-borate-EDTA (TBE) electrophoresis buffer. RNA fragments with the length of approximate 20-30 nts or 50-80 nts were extracted by gel crushing and further incubated with an RNA gel extraction buffer (300 mM NaOAc pH 5.2, 10 mM Tris-HCl pH 8.0, 1 mM EDTA pH 8.0) overnight.”*

The experiments done in fig 1 are exactly the same as those performed in (Guydosh & Green, Cell 2014) (ref 21). The text should reflect that. Please cite in the section "Translational pauses generate disomes from collisions of ribosomes".

*Response:*

*The work of Guydosh and Green (Cell 2014) has been duly cited in this section.*

It is not clear that the positional information between disome-seq and ribo-seq are comparable. Discrepancies between read densities at individual sites is cited as evidence these techniques capture different populations of ribosomes (fig 2). Such populations would differ in the 5'end of the cloned RNA fragment. As this is a site of considerable ligation bias (which the authors state), it is important to rule out the observed differences between disome and monosome-seq are not simply the result of 5'ligation biases.

*Response:*

*We thank Reviewer #2 for this insightful comment. We added three random nucleotides to the 3′-end of the 5′-adaptor to reduce the ligation bias in preparing the sequencing libraries. During revision, we also discussed the impact of ligation bias on disome-seq (in the section of Caveats, Page 14). Figure S9 has been added to the revised manuscript.*

*(Figure S9)*

*“The second confounding factor is ligation bias. RNA fragments with various 5′-nucleotide compositions may have variable ligation efficiency with a fixed 5′-adaptor sequence during the preparation of the high-throughput sequencing libraries [50, 51]. To reduce such ligation bias, we had added three random nucleotides to the 3′-end of the 5′-adaptor during library preparation. To assess how much ligation bias is remained, we calculated the frequency for each of the sixty-four 3-mers in the −15th to −13th nt region upstream of the A-site of monosome footprints, as well as in the −45th to −43th nt region upstream of the A-sites of the leading ribosomes detected in disome-seq. Both were positively correlated with the genome-wide tri-nucleotide composition in the coding sequences (Figure S9a-c), indicating that ligation bias has been largely eliminated from our monosome-seq and disome-seq libraries.*

*Nevertheless, we realized that any ligation bias not entirely eliminated could have led to discrepancies in the translational pauses detected by disome-seq and monosome-seq. It is because a translational pause generates different 5′-end for monosome and disome footprints (Figure S9a). To assess such possibility, we estimated the 3-mer frequencies in the −15th to −13th nt region upstream of the A-sites (for disomes, the A-site of the leading ribosome) for translational pauses detected in disome-seq or monosome-seq, respectively. Such 3-mers are the 5′-end of the 28-nt monosome footprints but are the internal sequences of disome footprints. Although monosome-seq and disome-seq identify largely non-overlapping pausing sites (Fig. 2e), the 3-mer frequency in the −15th to −13th nt region upstream of the A-sites were positively correlated between the two experiments (ρ = 0.95, P < 2.2×10-16, N = 64, Spearman’s correlation, Figure S9d). This observation indicates that ligation bias cannot be a major explanation for the discrepancy in the locations of translational pauses identified by disome-seq or monosome-seq.”*

The reads in the disome and monosome sequencing libraries appear to be taken at face value, without an attempt to correct for differences in raw material, sequencing depth, or mRNA expression. The raw mRNA in disomes is likely only a fraction that of monosomes (fig 1b, S1). There are also differences in sequencing depth ("uniquely mapped reads" in table S1). It's unclear the extent to which these effects factor into the numbers shown in fig 2b, 2c; a mere read count on transcripts in the absence of such corrections is not very biologically meaningful.

*Response:*

*We thank Reviewer #2 for this insightful comment. We have corrected it in the revised manuscript. We estimated the area ratio between the monosome peak and the disome peak in Figure 1b (right panel). This ratio (16.3) was used for the down-sampling of disome-seq reads. Specifically, 589461 uniquely mapped monosome reads (28 or 29 nts) were used to define “translated proteins”. We downsampled (589461/16.3/2 =) 18082 disome footprints (with lengths of 58, 59, 61, or 62 nts) and estimated the number of genes with disomes (or the positions of translational pauses for Fig. 2e). The fraction of translated genes with disomes indeed reduced after downsampling. Nevertheless, this fraction remained substantial (46%). These panels have been updated in the revised manuscript (Figs. 2c and 2e).*

*(Figs. 2c, e)*

To study the effects of the tripeptides identified in the disome-seq reads (fig 5a), the authors create a reporter (fig 5b). However, the run of amino acids is proximal to the start codon, and would not be sufficiently far into the CDS to allow a disome to form. It would be a good idea to point this out in the text to prevent readers from being misled.

*Response:*

*We thank Reviewer #2 for pointing out the flaw in our initial design. We have redesigned this reporter system, in which variants encoding triple-lysine was inserted far from the start codon (between Renilla-luciferase and firefly-luciferase). The elongation inhibition by triple-lysine was detected with this new dual-luciferase reporter (Fig. 5c).*

The conclusion of fig 5b ("polylysine decelerates translation elongation") is not justified. The sequence identified as most significant in fig 5a (KKK) was not tested, and the closest (KK, KKKK) have no or possibly mild effects. The larger effects seen at larger K lengths could be due to other known effects of this amino acid on degradation of the nascent peptide.

*Response:*

*The triple-lysine has been tested now using the new dual-luciferase reporter (Fig. 5c, pasted above). A significant reduction in the firefly/Renilla luciferase-activity ratio was observed. We have removed the results of larger lysine lengths in the revised manuscript.*

*(Page 9, Paragraph 1)*

*“To experimentally determine if combinations of synonymous codons for triple-lysine have different efficacies to induce translational pauses, we constructed a dual-luciferase reporter system by inserting individual synonymous variants of triple-lysine between the Renilla and firefly-luciferases (Fig. 5c). A reduction in the firefly/Renilla luciferase-activity ratio can report translational pausing induced by the inserted sequence — translational elongation replaces initiation as the rate-limiting step in protein synthesis of the downstream luciferase. We did not detect significant differences in the firefly/Renilla ratio among the eight synonymous codon combinations of triple-lysine (P = 0.57, analysis of variance, Fig. 5c). In contrast, the firefly/Renilla ratio was significantly reduced when triple-lysine was inserted (P = 8×10-4, the two-tailed t-test, Fig. 5c), suggesting that lysine residues, instead of the non-optimal AAA codons, are accountable for the ribosome collisions in A-tracts.”*

Both fig 4d, 5c show the distribution of disome-seq reads about the end of an alpha helix. What is this relative to? If the data is intended to show an enrichment, one must show a background expectation.

*Response:*

*We did not make it clear. We did not intend to show enrichment in these two figures. Reviewer #1 thought that it was not important to show this result if no enrichment was observed. We removed these two panels during revision.*

The interpretation of fig 7 is not justified ("ribosome pauses or collisions recruit chaperones"). Barring normalization issues (see major point, above), the data is also consistent with chaperones inducing stalling and collisions. Association between two entities (in this case, chaperones, disomes), cannot be taken as a causal relationship, nor a directional one.

*Response:*

*Thanks for the suggestion. We have removed the statements on “recruitment” in the entire manuscript.*

It may be useful to examine the prevalence of mass spec peptides upstream v downstream of disomes, akin to what was done in fig S9B, but more generally and with respect to disome-seq reads. This analysis would strengthen the idea that the disome footprints and mass spec peptides are derived from similar complexes.

*Response:*

*We thank Reviewer #2 for this suggestion. This comparison may not work because the nascent peptide’s intrinsic bias in mass spectrometry will confound the results of this test. Briefly, since we collected only ribosome-associated proteins, the nascent peptides were extracted. Because translation was not completed for these proteins, the peptides in the first half of the coding sequences tend to be identified (e.g., Figure S7B). Such bias will confound the comparison suggested by Reviewer #2.*

*Nevertheless, we agree with Reviewer #2 that additional analysis would help confirm that the disome footprints and mass spectrometry peptides are derived from similar ribosomal complexes. To this end, we estimated the intensity of a nascent peptide in the disome fraction (by mass spectrometry) and the abundance of disome footprints in its encoding gene (by disome-seq). We found them positively correlated (Figure S7c below, added to the revised manuscript).*

*(Figure S7c)*

*“Second, the protein abundance of these metabolic enzymes in the disome fraction was positively correlated with the abundance of disome footprints on their encoding gene (ρ = 0.57, P = 2.5×10−3, N = 26, Spearman’s correlation, Figure S7c). By contrast, we did not detect such correlation for ribosomal proteins (ρ = 0.16, P = 0.08, N = 129, Spearman’s correlation, Figure S7c); this is not unexpected since most ribosomal proteins were likely captured by MS/MS as ribosome components instead of as nascent peptides.”*

While the statistics in the manuscript was generally good, there were a few issues. Fig 4b uses pearson correlation, but the data does not fit a gaussian, so the test should be spearman correlation. Fig 4g uses a paired t-test, which again assumes the data fits a gaussian which it does not. While the p-values for these tests will change, the overall conclusions of the figure don't, so this is a minor point.

*Response:*

*Following the suggestion, we used the paired Mann-Whitney U test to access statistical significance in Figure 4g (P < 2.2×10-16). However, we have removed this panel following the suggestion of Reviewer #1.*

*For Figure 4b, we agree that Pearson’s correlation is impropriated, but we also think that Spearman’s correlation will lose the quantitative information. We therefore performed a linear regression to test if the propensity to terminate an α-helical conformation can be predicted from the P-site pausing score. We realize that linear regression requires the residuals to follow the normal distribution, which we specifically tested with Kolmogorov–Smirnov test (the P values of the Kolmogorov–Smirnov tests have been added to the figure legends in the revised manuscript).*

*For Fig. 4c*

*“The P-value was given by linear regression. The dashed line represents the linear regression line (if statistically significant). The residuals of both linear models followed the normal distribution (P = 0.63 and P = 0.80, the Kolmogorov-Smirnov tests).”*

*For Fig. 4d*

*“The P-value was given by linear regression. The dashed line represents the linear regression line (if statistically significant). The residuals of both linear models followed the normal distribution (P = 0.25 and P = 0.89, the Kolmogorov-Smirnov tests).”*

Fig 5b: the y-axis labels on the top graph appear to be misaligned.

*Response:*

*This panel has been replaced by the results of a new dual-luciferase experiment. We have checked the labeling for all figures in the revised manuscript.*

There's a typo on page 10 line 45; fig S6 is referenced rather than fig S8.

*Response:*

*Corrected.*

**Second round of review**

**Reviewer 1**

The authors have addressed all my comments in a satisfactory fashion.

**Reviewer 2**

I enjoyed reading the updated manuscript. The authors have adequately address my concerns, and in so doing, raised some exciting new questions. I am in favor of publication.
